# Detecting selection using extended haplotype homozygosity (EHH)-based statistics in unphased or unpolarized data

**DOI:** 10.1371/journal.pone.0262024

**Published:** 2022-01-18

**Authors:** Alexander Klassmann, Mathieu Gautier

**Affiliations:** 1 Institute for Genetics, University of Cologne, Cologne, Germany; 2 CBGP, Univ Montpellier, CIRAD, INRAE, IRD, Institut Agro, Montpellier, France; Government College University Faisalabad, PAKISTAN

## Abstract

Analysis of population genetic data often includes a search for genomic regions with signs of recent positive selection. One of such approaches involves the concept of *extended haplotype homozygosity (EHH)* and its associated statistics. These statistics typically require phased haplotypes, and some of them necessitate polarized variants. Here, we unify and extend previously proposed modifications to loosen these requirements. We compare the modified versions with the original ones by measuring the false discovery rate in simulated whole-genome scans and by quantifying the overlap of inferred candidate regions in empirical data. We find that phasing information is indispensable for accurate estimation of within-population statistics (for all but very large samples) and of cross-population statistics for small samples. Ancestry information, in contrast, is of lesser importance for both types of statistic. Our publicly available R package rehh incorporates the modified statistics presented here.

## 1 Introduction

The ease with which genomic sequences can be obtained contrasts sharply with the challenge of discerning their functional elements. Finding molecular signatures of recent selection can help to prioritize regions for further investigation. The search for selection is often performed by statistical tests refuting the null hypothesis of neutral evolution. Here we focus on the classic case of detecting recent strong positive selection in the form of a hard selective sweep, i.e., a single new advantageous variant replacing—on its way to fixation—all or most of previous variants [1]. Differential selection across populations can be detected by means of a conceptually simple statistic such as *F*_st_ [2] (which compares variant frequencies between populations) but may be corroborated by more sophisticated approaches, including those presented here, which exploit other characteristics of the selection signal. In contrast, the detection of selection within a single population has proven more challenging with various methods intended to capture a sign of a reduction in genetic variation [3, 4]. Measures of the average sample homozygosity and length of “runs of homozygosity” in individuals can be regarded, in our opinion, as pre-stages of the site frequency spectrum (SFS)-based and extended haplotype homozygosity (EHH)-based statistics presented here, respectively. We should remind the reader that the former simply shows the number of variants sharing the same sample frequency in a specific genomic region and, in contrast to the latter, disregards any association or linkage between them [4]. Hands-on overviews are provided by the authors of [5] (bioinformatic tools and workflow), [6] (methods and formulas), and [7] (a detailed collection of “recipes”). In the text below, we confine ourselves to three approaches that have been widely employed for more than a decade [8]:

- Tajima’s D [9], Fay & Wu’s H [10], and related metrics [11] compare the observed SFS of a genomic region with its expectation under neutrality. They are designed for regions short enough to ignore recombination. Although easy to apply and fast to compute, they are highly vulnerable to confounding effects of demography and population structure. They are implemented in various software packages such as dnasp [12] and R PopGenome [13].
- SweepFinder [14, 15] and SweeD [16] are two implementations of the same method. They take into account the frequency spectrum around specific chromosomal positions and calculate the composite likelihood ratio of a fitted sweep model (assuming gradual erosion of the signal of selection with increasing genetic distance) to a position-independent null spectrum. The latter is either taken from the empirical genome-wide “background” or derived from an explicit demographic model.
- Sabeti et al. [17] have introduced the concept of *EHH* on top of which Voight et al. [18] have built a statistic called iHS with subsequent variations [19, 20]. The statistic measures the decay (of linkage around a specific site) due to both recombination and mutations. iHS was first implemented in an eponymous program by the authors themselves [18]. Subsequent improvements have been implemented in Selscan [21], hapbin [22], and the R package rehh [23, 24].

In our view, there are two major differences of EHH-based techniques from SFS-based approaches (see our S1 Text for a short review of the latter):

- Fay & Wu’s H and SweepFinder/SweeD are constructed to detect completed selective sweeps, whereas EHH-based statistics are focused on ongoing selective sweeps. At least in humans, completed selective sweeps seem to be rare [25], and prime examples of selection, such as variants influencing the expression of the LCT gene (discussed below), are still far from fixation [26].
- Tajima’s D and similar quantities refer to genomic intervals, and although SweepFinder/SweeD compute scores for exact genomic positions, these are not directly associated with any particular polymorphism. In contrast, EHH-based statistics are tied to specific sites.

SFS methods, except original Tajima’s D, exploit the situation where alleles are polarized, i.e., the ancestral vs. derived state of each allele is known. Polarization is typically achieved using an outgroup: if a homologous site is monomorphic in the outgroup and coincides with one of the alleles in the investigated population, then that variant is called ancestral. Nonetheless, an outgroup species needs to be chosen properly: if on the one hand, the outgroup is phylogenetically too distant, then the probability of multiple mutations is high; if on the other hand, the outgroup is too close, then the probability of shared polymorphisms is high. Both scenarios lead to mis-specified ancestry status [27, 28]. Furthermore, a reference genome of that species has to be available. Even so, the genomes of the outgroup and of the focal species may not completely overlap, thereby leaving unpolarized chunks. For example, although considerable effort has been made to infer the “ancestral sequence” of present-day humans, ∼4% of the single-nucleotide polymorphisms (SNPs) found by the 1000 Genomes Project cannot be polarized (see below).

In addition to polarization, the calculation of EHH as described by [17] requires genotype data to be phased, i.e., it is known for di- or polyploid individuals which variant of a heterozygous locus belongs to which chromosome. Although obtaining phased haplotypes experimentally is expensive, computational methods for inferring them probabilistically often yield satisfactory results [29]. Nevertheless, two studies with the same basic approach indicate that phasing can be omitted in case of diploid individuals: [30] for a within-population test and [20] for a cross-population test. Both research groups assessed statistical power by simulations, yet they did not directly compare phased and unphased estimators; the latter group merely reported a coefficient of correlation *r*^2^ of 65–73% between the two estimators in terms of empirical data.

The aim of this article is to assess the robustness of EHH-based statistics against a loss of information about the phase or variant ancestry status. We first recapitulate and unify the definition of the three statistics we want to investigate. Then, we describe how the statistics can be adapted to account for unphased and/or unpolarized data. For the within-population test, we compared the false discovery rate (FDR) between original and modified statistics in simulated whole-genome scans and collated them with the above-mentioned frequency spectrum–based methods; we limited our simulations to a single evolutionary scenario that we deem, despite its simplicity, sufficient to provide a qualitative picture. For all three statistics, we calculated the overlap of candidate regions found by means of original and modified versions on empirical data. Along the way, we aimed at giving potential users an intuitive feel for the various statistics involved.

## 2 Materials and methods

### 2.1 Definitions of statistics iHS, XP-EHH, and Rsb

At the beginning, we want to clarify that the word *homozygosity* as part of the term EHH refers to the probability that two randomly chosen chromosomes from a population are identical (at a certain locus or region).

Let *s* denote a site of interest within a chromosome. We call *s* the focal marker (whereas Wang et al. [30] use the term primary locus) and designate variants at that marker as core alleles. Suppose *n*_*a*_ means the number of sequences with core allele *a*, and *n*_*s*_ = ∑_*a*_ *n*_*a*_ represents the total number of sequences. If there are no missing data at the focal marker, then *n*_*s*_ equals (haploid) sample size *n*. All chromosomes sharing a core allele are by definition homozygous at the focal marker. EHH measures the decay of this homozygosity with increasing distance to the marker and is calculated independently in each direction (upstream/downstream) from the marker. To be precise, suppose *t* is another marker on the same chromosome, and let us consider the region between *s* and *t*. Any two (or more) chromosomes identical in that region constitute a shared haplotype. Let *K*_*s*,*t*_ denote the number of all distinct shared haplotypes in the sample, and $K_{s,t}^{a}$ the subset with allele *a* at focal marker *s*. *n*_*k*_ refers to the number of sequences sharing haplotype *k*. Quantity *EHH*^*a*^ as defined by ref. [17] is calculated for chromosomes carrying core allele *a* as

$$\begin{array}{c} {\text{EHH}}_{s,t}^{a}=\frac{1}{n_{a}\left(n_{a}-1\right)}\sum_{k=1}^{K_{s,t}^{a}}n_{k}\left(n_{k}-1\right)\;. \end{array}$$ (1)

To summarize $EHH_{s,t}^{a}$ as a single number assignable to allele *a* at site *s*, Voight et al. [18] have opted for the integration of EHH and named the resulting quantity integrated haplotype homozygosity (iHH):

$$\begin{array}{c} {\text{iHH}}^{a}\left(s\right)=\int EHH_{s,t}^{a}\;dt\;. \end{array}$$ (2)

The integration is performed numerically and stopped when *EHH*, monotonically decreasing with increasing distance to the focal marker, reaches a lower threshold or cutoff, usually set to 0.05.

Note that although *iHS* has historically been defined in this two-step way, it is equivalent but conceptually simpler to regard it as the average of lengths *l*_*ij*_(*s*) of shared haplotypes among all pairs of chromosomes *i* and *j* carrying core allele *a*:

$$\begin{array}{c} {\text{iHH}}^{a}\left(s\right)=\frac{1}{n_{a}\left(n_{a}-1\right)}\sum_{i\neq j}^{n_{a}}l_{ij}\left(s\right)\;. \end{array}$$ (3)

Given *iHH* for ancestral (A) and derived (D) alleles of a focal marker, Voight et al. [18] have favored a log-ratio for their comparison, yielding the (as yet unstandardized) integrated haplotype homozygosity score (iHS)

$$\begin{array}{c} \text{uniHS}\left(s\right)=\text{ln}(\frac{{\text{iHH}}^{A}\left(s\right)}{{\text{iHH}}^{D}\left(s\right)})\;. \end{array}$$ (4)

Finally, this statistic is standardized:

$$\begin{array}{c} \text{iHS}\left(s\right)=\frac{\text{uniHS}\left(s\right)-\text{mean}\left(\text{uniHS}|p_{s}\right)}{\text{sd}(\text{uniHS}|p_{s})}\;. \end{array}$$ (5)

Because the expected values under neutrality of uniHS strongly depend on derived allele frequency *p*_*s*_ at focal marker *s*, the standardization is ideally performed separately for all markers with the same frequency. In practice, the standardization is carried out across small frequency bins. Voight et al. [18] state that iHS approximately follows a standard normal distribution.

To detect selection using iHS, both alleles of a site must be present in enough sequences for obtaining a reliable estimate of their respective *EHH*^*a*^. Typically, a minor allele frequency (MAF) of at least 5% is required, which excludes variants near fixation.

To overcome this limitation, Sabeti et al. [19] and Tang et al. [20] have independently modified the above statistic to compare two populations instead of two alleles. Although Sabeti et al. [19] have kept the term EHH, we follow Tang et al. [20] in distinguishing site-specific EHH by means of EHHS:

$$\begin{array}{c} {\text{EHHS}}_{s,t}=\frac{1}{n_{s}\left(n_{s}-1\right)}\sum_{k=1}^{K_{s,t}}n_{k}\left(n_{k}-1\right)\;. \end{array}$$ (6)

Keep in mind that *EHHS*_*s*,*s*_ is an estimate of the focal marker’s homozygosity. Subsequent statistics are built analogously to Eqs (2)–(5). Sabeti et al. [19] first integrated this statistic to calculate integrated EHHS (iES)

$$\begin{array}{c} \text{iES}\left(s\right)=\int EHHS_{s,t}\;dt\;, \end{array}$$ (7)

which is then compared between two populations to obtain as yet unstandardized XP-EHH

$$\begin{array}{c} \text{unXP-EHH}\left(s\right)=\text{ln}(\frac{{\text{iES}}_{\text{pop1}}\left(s\right)}{{\text{iES}}_{\text{pop2}}\left(s\right)})\;, \end{array}$$ (8)

which in turn is standardized, yielding

$$\begin{array}{c} \text{XP-EHH}\left(s\right)=\frac{\text{unXP-EHH}(s)-\text{mean}(\text{unXP-EHH})}{\text{sd}(\text{unXP-EHH})}\;. \end{array}$$ (9)

The approach from ref. [20] differs in so far as *EHHS*_*s*,*t*_ is normalized to its value at marker *t* = *s*. Thus, we refer to the integral as an integrated normalized EHHS score:

$$\begin{array}{c} \text{inES}\left(s\right)=\frac{1}{EHHS_{s,s}}\int EHHS_{s,t}\;dt=\frac{iES(s)}{EHHS_{s,s}} \end{array}$$ (10)

to obtain first the (unstandardized) ratio between populations (Rsb). Note that for the sake of uniformity, our notation differs slightly from that given in ref. [20], where (12) is referred to as ln(Rsb), and the log-nontransformed value is used only for plotting.

$$\begin{array}{c} \text{unRsb}\left(s\right)=\text{ln}(\frac{{\text{inES}}_{\text{pop1}}\left(s\right)}{{\text{inES}}_{\text{pop2}}\left(s\right)})\;, \end{array}$$ (11)

and, finally, we standardize by the median instead of the mean,

$$\begin{array}{c} \text{Rsb}\left(s\right)=\frac{\text{unRsb}(s)-\text{median}(\text{unRsb})}{\text{sd}(\text{unRsb})}\;. \end{array}$$ (12)

It should be noted that for standardization of cross-population statistics XP-EHH and Rsb, no binning with respect to core allele frequencies is undertaken and hence no variant polarization is presupposed.

### 2.2 Modifications for unphased sequences

The probability that two sequences of a population are identical can be estimated not only by a pairwise comparison of all sequences in a sample (as formulated above) but also via the proportion of homozygous diploid individuals, under the assumption of the Hardy–Weinberg equilibrium. The latter does not require phase information, and the authors of [20, 30] have used the idea to estimate *EHH* (under a different name) and *EHHS*, respectively: the crucial difference from Eqs (1) and (6), respectively, is that only the two chromosomes of each individual are compared. Statistics *EHH* and *EHHS* are then estimated as above via the proportion of shared haplotypes among all sequence comparisons. Let *I*_*s*,*t*_ denote the number of individuals homozygous in the region between *s* and *t*, and suppose $I_{s,t}^{a}$ represents those among them that carry core allele *a*. At marker *t*, quantities *EHH*^*a*^ and *EHHS* are respectively estimated as

$$\begin{array}{c} {\text{EHH}}_{s,t}^{a}=\frac{I_{s,t}^{a}}{I_{s,s}^{a}} \end{array}$$ (13)

$$\begin{array}{c} {\text{EHHS}}_{s,t}=\frac{I_{s,t}}{I_{s,s}}\;. \end{array}$$ (14)

**Fig 1:**
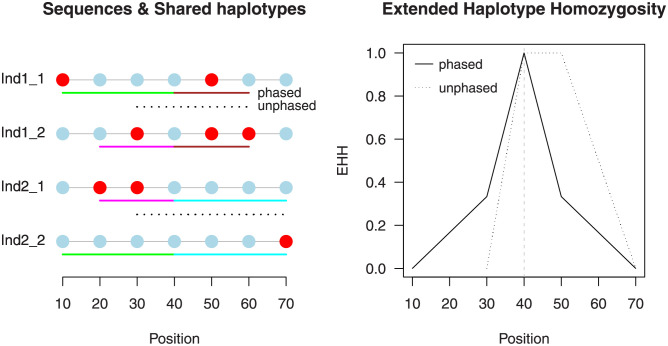
An example of the calculation of *EHH* using the estimator for phased (Eq (1)) and unphased sequences (Eq (13)). The left-hand panel depicts the variants seen in four aligned sequences belonging to two diploid individuals. At the central marker (position 40), taken here as focal, all sequences share the same allele. Next to the sequences, the range of shared extended haplotypes around the focal marker is indicated. The boundaries of shared haplotypes are determined by the position of the marker that introduces a difference between the hitherto identical sequences. Without phase information, only the two sequences of each individual can be compared, and the resulting shared haplotypes are visualized by dashed lines. For instance, the two sequences of individual 1 become different at the first marker to the left of the focal marker, and consequently their shared haplotype ends at position 30. In contrast, when variants are phased, all sequences can be compared with each other. The panel depicts for each sequence its longest shared haplotype, indicated by a solid line, with the constituent sequences in the same color. The remaining shared haplotypes end at position 30 and 50, respectively. The right-hand panel shows the *EHH* values calculated at each marker position as the proportion of sequences sharing a haplotype among all comparisons. Note that the *EHH* curve is typically defined as linearly interpolating between consecutive markers (as depicted), although for completely sequenced data, a stepwise constant function would be more appropriate. With the latter definition, the integral over the *EHH* curve, *iHH*, becomes identical to the average length of shared haplotypes: $\frac{30+40}{2}=35$ and $\frac{180}{6}=30$ for unphased and phased sequences, respectively.

Fig 1 illustrates the original and modified way to estimate *EHH* (and *iHH*). All subsequent steps to obtain iHS, XP-EHH, and Rsb remain the same as above. Because EHHS calculated via Eq (14) is normalized (giving 1.0 at the focal marker), for unphased data, XP-EHH is essentially identical to Rsb; they differ only in the use of the median and mean, respectively, at the standardization step.

It must be pointed out that only the chromosomes of individuals homozygous at that focal marker can share a haplotype. The resulting set of mutual chromosome comparisons is hence a (typically much smaller and possibly even empty) subset of those made by the original approach.

**Fig 2:**
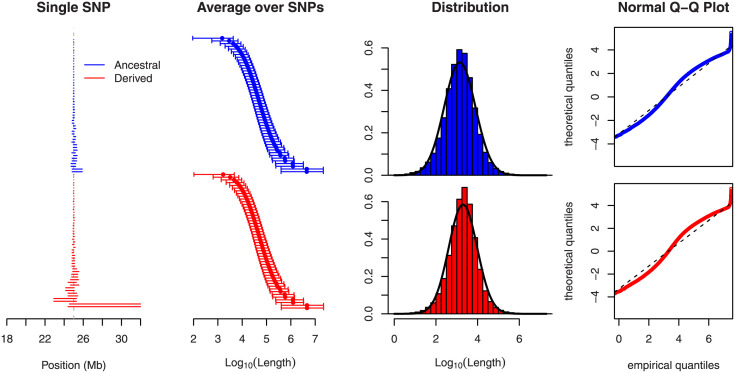
The length of shared haplotypes. A region of 50 Mb was simulated in a neutrally evolving population with a sample size of *n* = 100. We considered only SNPs where both core alleles have a sample frequency of 50%, and we assumed that the phase is known. As in the middle panel of Fig 1, the lines in the left panel symbolize the range of the longest shared extended haplotypes, here for the most central SNP in the first simulation, ordered along the y-axis by their length. The extreme length of a single shared haplotype stands out. The middle left panel indicates that this is not an exceptional feature: here, shared haplotype lengths (restricted to those to the “right” of the focal marker) are averaged across SNPs from 100 independent simulation runs, restricted to those less than 5 Mb away from the center in order to minimize boundary effects. The ends of the bars represent 5% and 95% quantiles. For the same SNPs, the middle right panel presents length distributions of all pairwise shared haplotypes ($\frac{50\cdot 49}{2}$ per SNP and allele). The distributions are overlaid with a fitted Gaussian curve. The right panel shows Q–Q plots of the distributions. Note that the largest lengths are actually capped, because in 11 simulation runs, shared haplotypes reached the chromosomal boundary.

Fig 2 shows why this state of affairs entails a major problem: the length of shared haplotypes is distributed very unevenly among the chromosomes of a sample, and even in the absence of selection, a few shared haplotypes of extreme length can occur. In small samples, these can easily give rise to “outlier” values of the final statistics, thereby confounding the signal arising from selection. In an attempt to reduce this statistical noise, we imposed the following restrictions:

- only focal markers with at least 10 homozygous sequences (five individuals) are considered: sample-wise for XP-EHH and Rsb and independently for each core allele in case of iHS (the latter on top of the original requirement of a MAF of at least 0.05),
- the cutoff that stops integration of EHH/EHHS is increased from its original value of 0.05 to 0.10,
- another integration cutoff is added, leading to stoppage when fewer than four chromosomes (two individuals) remain homozygous (for the original statistics, this condition follows from the preceding two).

### 2.3 Modifications for unpolarized variants

There is only one step where information about allele ancestry status is exploited, namely, the standardization of *uniHS* in Eq (5), depending on the frequency of the derived core allele. To avoid arbitrary assignment of ancestry status, we replaced the ancestral and derived allele in Eq (4) by a major (most frequent) and minor (second most frequent) allele, respectively.

$$\begin{array}{c} \text{uniHS}\left(s\right)=\text{ln}(\frac{{\text{iHH}}^{MAJ}\left(s\right)}{{\text{iHH}}^{MIN}\left(s\right)})\;. \end{array}$$ (15)

For unpolarized variants, the frequency dependence of *EHH* under neutrality cannot be accounted for by a binning with respect to MAF because such a binning would group derived alleles of frequency *p*_*s*_ together with those of frequency *p*_1−*s*_, whose respective expected values differ increasingly with decreasing MAF. Hence, for lack of a better solution, we suggest that standardization be performed without considering allele frequencies:

$$\begin{array}{c} \text{iHS}\left(s\right)=\frac{\text{uniHS}(s)-\text{mean}(\text{uniHS})}{\text{sd}(\text{uniHS})}\;. \end{array}$$ (16)

### 2.4 Delineation of regions under selection

Ref. [18] shows that stand-alone markers with extreme values of iHS are less indicative of selective sweeps than a cluster of high values (see Fig 2, S2 Text). In effect, those authors identified intervals that are candidates for selection by requiring that half of markers have values above the 99th genome-wide percentile. We followed this approach with a modification: we adapted the threshold value to obtain a fixed number of candidate regions. We used overlapping sliding windows of width 250 kb with an offset of 50 kb, and overlapping candidate windows were merged. For empirical data, we decided that the number of markers in any window had to exceed the (arbitrary) value of 150 to exclude regions with few genotyped markers; if the phase was ignored, then this number was halved for iHS, corresponding to a similar decrease for the markers for which a score could be obtained.

To facilitate the comparison, we applied sliding windows of the same size and overlap to the values of the frequency spectrum–based tests, although here, stand-alone markers had to exceed a given threshold. Because the values of Tajima’s D and Fay & Wu’s H are calculated for intervals, we designated the interval centers as the corresponding positions.

### 2.5 Whole-genome scans in simulated data

We performed coalescent simulations using msms [31]. We assumed an effective population size of *N*_*e*_ ≈ 10, 000 for humans. In some simulation studies, both a population-scaled mutation rate and recombination rate have been set to *θ* = *ρ* = 0.001 per base per generation [32, 33], and we followed this approach for simplicity, although we should acknowledge that depending on the estimation method, for humans, rates of half that size can be inferred for both quantities [34–37].

For our simulated regions, we set population-scaled rates *θ* and *ρ* both to 50, 000; thus, they corresponded to a physical length of 50 Mb in humans. This large size proved necessary to reduce boundary effects, because as displayed in Fig 2, shared haplotypes can span several megabase pairs even under neutrality. We ignored the fact that recombination events in reality occur within hot spots [38] because msms cannot handle varying recombination rates, whereas other tools that can (e.g., msHOT [39]), are not able to simulate selection. We could, nonetheless, replicate our results under neutrality by means of msprime [40]. To investigate distributional properties under neutrality, for iHS, we simulated chromosomes evolving in a single constant-size population, and for XP-EHH/Rsb, in two neutrally evolving populations that split symmetrically from an ancestral population 4*N*_*e*_ ⋅ 0.05 generations ago (∼50,000 years in humans), without subsequent migration.

To analyze selection signals, we created a “genome” consisting of 100 independently simulated samples of chromosomes, each experiencing a single ongoing selective sweep located at its center while otherwise evolving neutrally. The selected allele was designated as fully dominant with a population-scaled selection coefficient of 2*N*_*e*_ *s* = 500—having reached at sampling time a population frequency of 50%, 70%, or 90%—or at a population-scaled time of 0.01 (corresponding roughly to 10000 years in humans) after fixation. The simulated (haploid) sample size was *n* = 400, from which we took subsamples down to sample size *n* = 50. For the calculation of statistical power, we created a neutrally evolving “genome” of 20 independent chromosomes with parameters otherwise identical to those above.

To these genomes, we applied the original or modified iHS statistics. For the estimator with unphased data, we tried two cutoffs: the standard one of *EHH* = 0.05 and a more stringent cutoff of *EHH* = 0.10. Furthermore, we computationally reconstructed phase information from randomized genotypes using fastPHASE [41] with subsequent application of the original statistics. Additionally, we computed values for Tajima’s D [9] and Fay & Wu’s H [10] as well as the composite likelihood score as implemented by SweepFinder [15] and SweeD [16]. The latter was calculated with and without allowance for variant ancestry status.

To evaluate the performance of the various approaches, we estimated the FDR and statistical power. A delineated candidate region was considered a “true positive” when it overlapped with a true selected site. Consequently, the FDR measures the proportion of mislocated regions among regions deemed significant. For each statistic and sample size, the significance threshold was adjusted so as to call exactly 100 candidate regions. With these settings, the lower the FDR, the more optimal is the test. The FDR is zero when each of the 100 simulated selected sites is identified by means of a distinct candidate region. If, on the contrary, candidate regions are assigned to random places within the genome, then the probability of a “true positive” equals combined length of all candidate regions divided by genome length; in this case, the expected FDR is 1.0 minus this probability. Note that the number but not the length of delineated candidate regions is fixed because a region may comprise several merged windows. For the computation of statistical power, we adjusted a threshold such that approximately 1% of the neutral genome was (falsely) designated as selected. The thresholds were calculated for each statistic and each sample size independently and then applied to the genome undergoing selection.

See S1 Protocol regarding software and technical details.

### 2.6 Whole-genome scans in empirical data

We used data from ref. [42], where researchers called variants on reads (from the 1000 Genomes Project [43]) realigned to human reference genome assembly GRCh38. The data comprise only autosomes and contain fully phased biallelic SNPs with imputed missing values. The ancestral alleles, inferred from an alignment of 12 primates, were obtained from ENSEMBL release 91 [44]. Almost 91% of the 73 million SNPs are covered by ancestral states classified as “high confidence” and another 6% as “low confidence”; using both, we were able to polarize 95.8% of SNPs. We calculated the statistics for samples of European origin (CEU and GBR), Asian origin (CHB and JPT), and African origin (YRI; see Table 1). Additionally, we combined the samples of two closely related populations (see S5 Table of ref. [43]), namely the two European samples mentioned and Chinese samples CHB and CHS, respectively.

**Table 1:** The population samples of the 1000 Genomes Project used in this study.

Sample	Population	# Individuals
CEU	Central Europeans in Utah (CEPH individuals)	99
CHB	Han Chinese in Beijing, China	106
CHS	Han Chinese South, China	105
GBR	British from England and Scotland	100
JPT	Japanese in Tokyo, Japan	105
YRI	Yoruba in Ibadan, Nigeria	107

We assessed the robustness of the statistics vis-à-vis a loss of phase or ancestry information by means of the number of overlapping candidate regions.

## 3 Results

### 3.1 General properties of the statistics under neutrality

#### 3.1.1 Dependence on core allele frequencies

Under neutrality, we examined the dependence of the three original statistics on the frequency of derived allele *p*_*s*_ at focal marker *s*. For *uniHS*, this was already reported in ref. [18] (see their Fig 4). By recalculating uniHS using subsamples containing an equal number of the two core alleles, we confirmed that uniHS indeed depends on population frequency of the derived allele and is not an artifact of its sample frequency (Fig 1 of S2 Text, left and middle panel).

**Fig 3:**
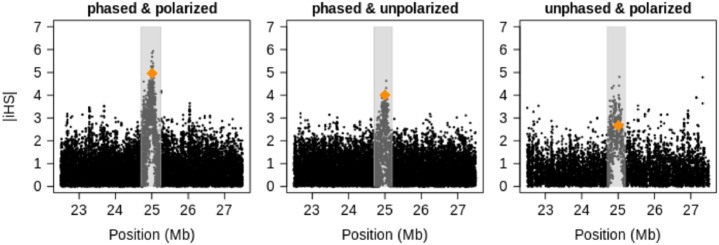
iHS values of a single simulation “run” (arbitrarily chosen as the first of the 100 runs) around a site containing a selected variant of population frequency 70% at a sample of size *n* = 200. The value for the site with the selected variant is highlighted in dark orange, and identified regions that are candidates for selection are marked in gray. See also Figs 8–15 of S2 Text.

Cross-population statistics XP-EHH and Rsb are defined symmetrically with respect to the compared populations, and as a consequence, the expected values have to be zero for markers with the same derived allele frequency from populations of identical demography. This does not hold when derived frequencies differ between populations (Figs 2 and 3 of S2 Text): an observation not made by the authors of [19, 20] and consequently not taken into account at the standardization step. Fortunately, the effect is smaller than that for the uniHS statistics, making correction less necessary. Furthermore, frequency-dependent standardization in the vein of iHS would require two-dimensional bins, and contrary to iHS, the implicit assumption that each bin is dominated by neutral variants does not hold because large frequency differences are indicative of differential selection. Consequently, in the absence of a better solution, we continue to utilize these statistics as is. Note, however, that any such hypothetical bin-wise standardization would make *XP-EHH* and *Rsb* essentially identical, except for the respective use of the mean and median in Eqs (9) and (12).

#### 3.1.2 Distributions of the statistics

**Fig 4:**
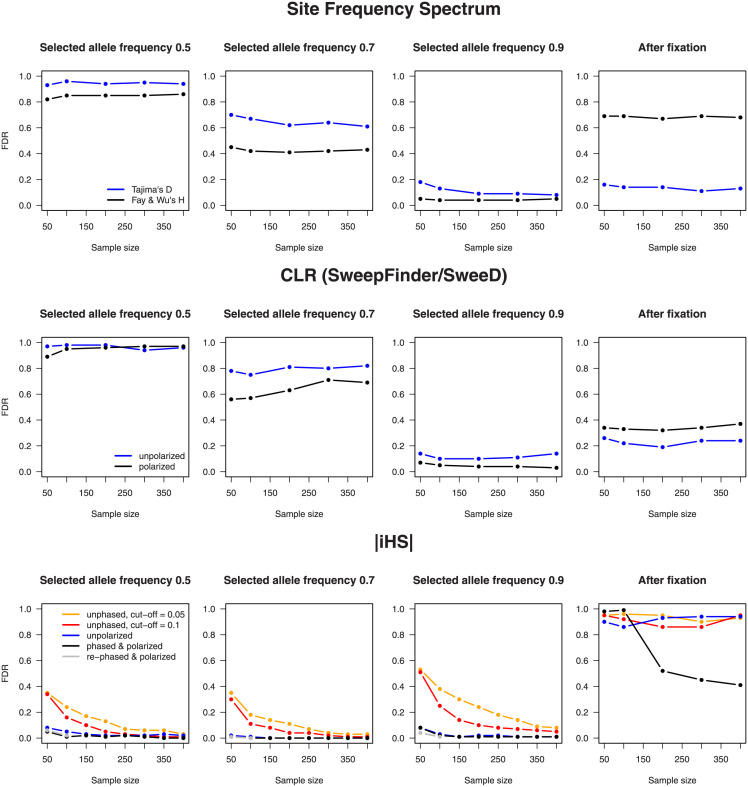
A comparison of the FDR among different statistics, sample sizes, and frequencies of the selected allele. A hundred regions–candidates for selection were delineated in a simulated genome containing 100 sites under selection. The FDR represents the proportion of incorrectly located regions, i.e., regions that do not overlap with any “true” site subject to selection. An ideal test should output an FDR of zero. Rephasing was performed only for sample sizes 50 and 100 with a still segregating selected variant.

Statistics iHS, XP-EHH, and Rsb have been constructed to be approximately standard-normally distributed under neutrality. Our simulations confirmed this principle for the original statistics while the modified ones manifested notable deviations: disregarding ancestry information leads to a skew in iHS values, and using the estimator for unphased variants results in “heavier tails” in all three statistics (Figs 4 and 6 of S2 Text). Both deviations can be easily explained. On the one hand, in a neutral setting, a sample is expected to contain much more variants of low derived frequency than of high frequency. Without frequency-wise normalization, the center of the resulting distribution of ihs will be closer to negative values of the low-frequency variants than to positive values of the high-frequency variants, hence yielding the skew for unpolarized data. On the other hand, the few very long shared haplotypes arising under neutrality are much more likely to give rise to extreme values when averaging by length is restricted to within-individual shared haplotypes, hence producing “heavier tails” for unphased data.

### 3.2 Whole-genome scans in simulated data

#### 3.2.1 A single selective sweep in detail

In Fig 3, we present an example of the iHS values obtained in the vicinity of a strongly selected variant located in the middle of a chromosome that otherwise evolves neutrally. The variant has reached a population frequency of 70%. It is evident that the omission of ancestry status causes a decrease of values around the selected site. Lack of the phase, by contrast, primarily increases statistical “noise” from the neutral part of the chromosome. This can be observed too for unstandardized iHS in the right-hand panel of Fig 1 in S2 Text. The relative lack of low values around the selected site in each case is a more prominent feature of the sweep than the attainment of extreme values is, thus giving us a reason to search for such “clusters.” Further examples, including those of the frequency spectrum–based tests and calculated for different sample sizes, are given in Figs 8–15 in S2 Text. These plots indicate that our requirement of at least 10 sequences per allele in unphased data is of lesser importance when the sample size is large but drastically reduces the number of suitable markers in small samples. Note that the selected variant neither necessarily has the most extreme value nor lies in the exact center of the region containing elevated values.

#### 3.2.2 The FDR and statistical power

**Fig 5:**
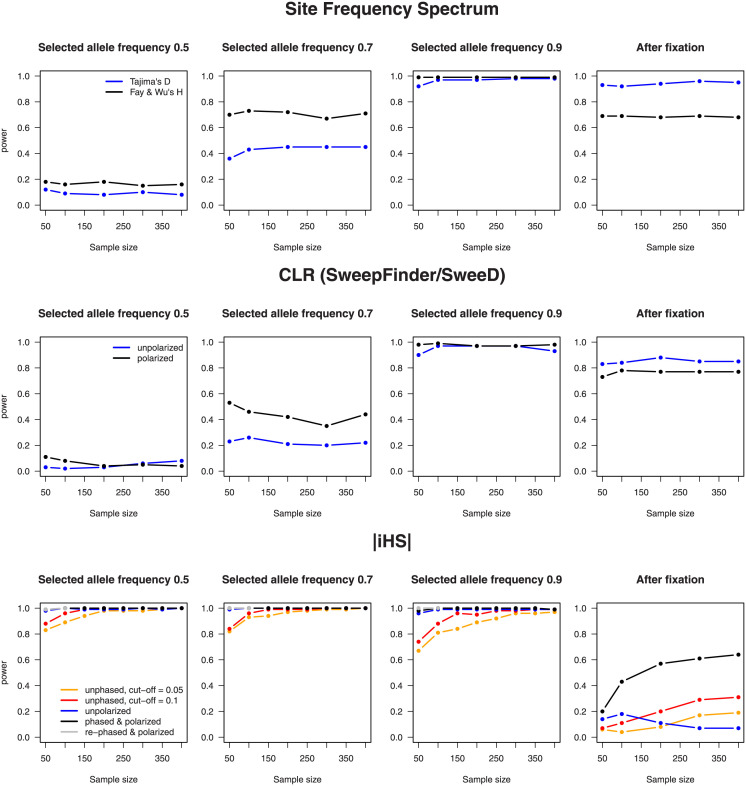
A comparison of statistical power among different statistics, sample sizes, and frequencies of the selected allele. These graphs are similar to those in Fig 4, but here, the proportions of correctly identified selected sites are shown, and thresholds were set such that 1% of a neutrally evolving genome is (falsely) designated as selected.

Figs 4 and 5 summarize the results of our whole-genome scan in simulated data. Fig 4 shows the FDR: the proportion of the 100 delineated candidate regions that did not overlap with one of the 100 true selected sites. Given that only the number of identified candidate regions was fixed a priori, we checked whether for each test and sample size, the average areas covered by the regions were of similar size. Indeed, they constituted ∼500 kb per chromosome, hence ∼1% of its length. Fig 5 presents statistical power: the proportion of true positives for a given significance level. Note that this level was set by adjusting thresholds such that identified candidate regions cover 1% of a neutrally evolving genome. The thresholds chosen for Fig 4 are therefore somewhat lower than those used for Fig 5, but other than that, the results are largely complementary.

First, we can see that ongoing sweeps in early stages can be better recognized by iHS than by frequency spectrum–based tests, whereas the opposite is true after the selected site is fixed. Second, in every case and almost independently of sample size, the lack of polarization yields an increase in the number of “false positives,” with the effect being smaller for iHS than for the other statistics. After fixation, knowledge of ancestry status reduces the FDR for iHS, although in this case, its overall performance remains poor. Third, the lack of the phase drastically increases the FDR for iHS for all but the largest sample sizes, and an increased cutoff offers only partial compensation. Lastly, at least in our high-density simulated data, computational phasing of genotypes is much more effective than applying the modified estimator to unphased sequences; to our surprise, the FDR turned out to be even somewhat lower for the reconstructed phase than for the “true” data. We do not know the reason and can only speculate that fastPHASE does not detect all recombination events, thereby increasing the length of shared haplotypes and hence the signal of selection.

### 3.3 Whole-genome scans in empirical data

#### 3.3.1 Two selective sweeps in detail

**Fig 6:**
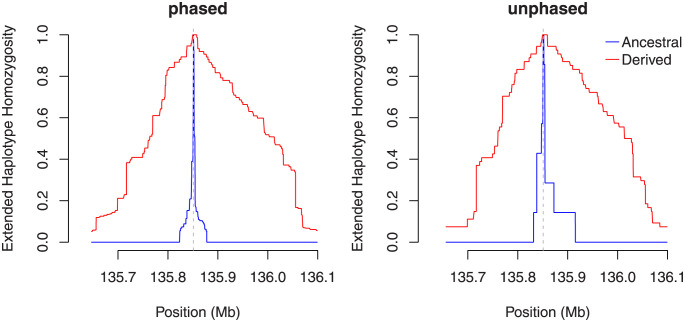
EHH for ancestral and derived alleles of SNP rs4988235 in population CEU from the 1000 Genomes Project. The SNP is located on chromosome 2, approximately 13 kb upstream (in 3′ direction) of the LCT gene.

**Fig 7:**
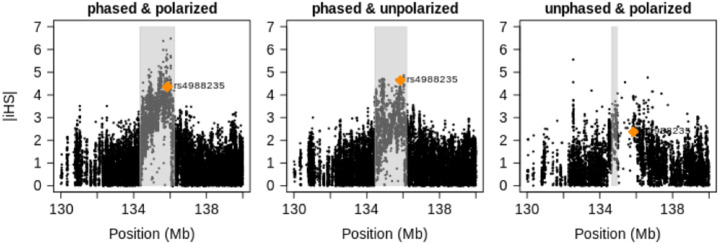
iHS values in a region around the LCT gene in population CEU. The value of the putatively selected site is highlighted in dark orange, and the identified regions–candidates for selection are marked in gray. That the putatively causal site has a more prominent score in unpolarized estimation is entirely accidental in our opinion.

Several variants in the enhancer of human gene LCT give lactase persistence, which enables adults to digest raw milk [45–47]. Although this capability is undisputedly under strong selection, the precise advantage of this trait is still debated [48]. Here we are concerned with SNP rs4988235 whose derived variant attains its highest frequency of 74% in population CEU, while it is virtually absent in all East Asian and nonadmixed African populations documented in the 1000 Genomes Project. Fig 6 depicts EHH around this SNP for its two alleles. Readers can see that EHH extends much farther for the derived variant than for the ancestral one: a sign that the allele has reached its current population frequency faster than under neutrality. The curves for EHH when the estimator for unphased data is employed are more coarse-grained but still quite similar in shape and scale. Fig 7 shows genome-wide standardized iHS values around the LCT gene. As with the simulated data, the omission of polarization leads to a reduction of high values but leaves the overall pattern intact. The omission of phasing instead causes a notable increase of “noise” in the sense that many low values get inflated. Again, the most conspicuous is the massive lack of values in the putative center of the sweep owing to our discarding the sites where the minor allele is present in fewer than 10 sequences (or five individuals). In fact, only seven individuals are homozygous for the ancestral allele of SNP rs4988235. Figs 16–19 of S2 Text indicate that the situation is similar in other candidate regions. Out of curiosity, we computed standard iHS values for additional populations as well (Fig 20 of S2 Text): almost all European populations from the 1000 Genomes Project have a similarly strong signal, while none of the African populations do. Nonetheless, another African population investigated within the HapMap3 project [49] shows a signal similar to that of Europeans [50].

**Fig 8:**
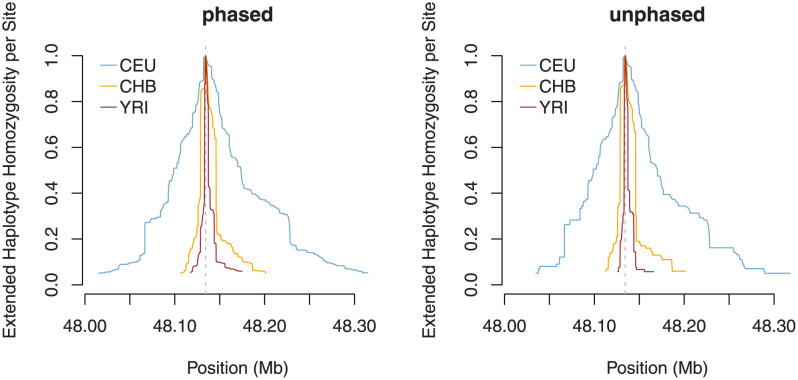
Normalized EHHS around SNP rs1426654 in populations CEU, CHB, and YRI. The SNP is located within gene SLC24A5.

**Fig 9:**
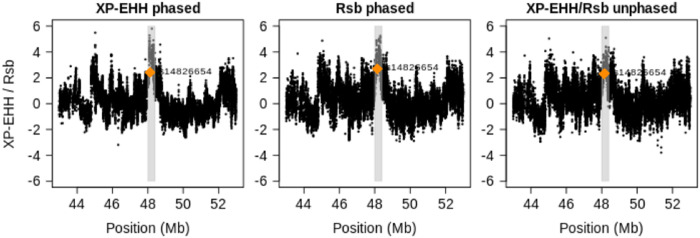
XP-EHH and Rsb values in a region around the SLC24A5 gene for a comparison of populations CEU and YRI. The value of the putatively selected site is highlighted in dark orange, and the identified regions–candidates for selection are marked in gray.

SNP rs1426654 within gene SLC24A5 causes the Ala111Thr polymorphism in the corresponding protein and influences skin pigmentation [51]. The level of pigmentation has to balance the opposing requirements: protection from UV radiation and ensuring sufficient vitamin D production [52]. The derived variant has low frequency in the African populations, is almost fixed in the European populations, and all but absent in the East Asian populations from the 1000 Genomes Project. Because population sample CEU is monomorphic for the derived variant, only cross-population statistics are applicable. Fig 8 shows that EHHS extends much farther in population CEU than in populations CHB and YRI. Again, ignoring phase information, we obtain a coarser but otherwise similar picture. In Fig 9, we compare the original XP-EHH and Rsb statistics with their counterpart for unphased data (where both statistics are essentially identical) around the SLC24A5 gene. The panels look quite similar, suggesting that the statistics are largely equivalent.

#### 3.3.2 Distributions of the statistics in empirical data

The statistics from empirical data have more extreme values, or in other words, their distributions have heavier “tails” than those seen in simulated neutral evolution, especially when the estimators for unphased data are applied (Figs 5 and 7 of S2 Text).

#### 3.3.3 The overlap of the found candidate regions

We are interested in whether the delineated putative regions under selection are robust with respect to the adjustments we made to the original statistics. As discussed in the section, we largely borrowed the settings from ref. [18] but adjusted the threshold value to obtain exactly 20 candidate regions for each statistic. Table 2 presents the number of overlapping regions when *iHS* is used. Readers can see that there is a considerable overlap between the regions called from the original statistics and those where ancestral information is ignored, whereas ignoring phase information yields only a modest overlap, even for large sample sizes. In Table 3, standard statistics Rsb and XP-EHH are compared with each other and with the version for unphased data. Here, the overlap between the modified statistics and the original ones is not much less than that between the two original statistics, except for the comparison of populations CHB and JPT. Because these two populations are rather similar, the signal of differential selection might be too small to detect without phasing.

**Table 2:** The number of overlapping identified regions that are candidates for selection, according to two different estimators of *iHS*. For each estimation of *iHS*, the threshold was adjusted to obtain exactly 20 candidate regions.

	iHS phased polarized/unpolarized	iHS polarized phased/unphased
CEU	10	2
CHB	12	1
JPT	9	2
YRI	14	5
CEU+GBR	11	4
CHB+CHS	12	3

**Table 3:** The number of overlapping identified regions–candidates for differential selection, as determined by two different estimators (phased and unphased) or two statistics (XP-EHH and Rsb). The threshold was adjusted to obtain exactly 20 candidate regions for each combination of an estimator and statistic. Note that unphased XP-EHH and Rsb are by definition almost identical and hence afford almost identical candidate regions.

	Rsb/XP-EHH phased	XP-EHH phased/unphased	Rsb phased/unphased	Rsb/XP-EHH unphased
CEU vs CHB	12	11	11	20
CEU vs JPT	11	9	14	18
CEU vs YRI	11	7	10	20
CHB vs JPT	13	4	3	20
CHB vs YRI	12	6	10	18
JPT vs YRI	11	8	11	20
CEU+GBR vs CHB+CHS	13	12	12	20

The precise chromosomal locations of all ascertained candidate regions as well as strengths of the signals are listed in S2 Text. The computed iHS, XP-EHH, and Rsb values are available on Dryad [53].

## 4 Discussion

While ever more sophisticated methods for detecting selective sweeps are being developed [54–56] and other, more subtle modes of selection [57] are under increasing scrutiny, the relatively simple summary statistics presented here will continue to serve as a first-pass analysis of population genetic data. The aim of our study was to test whether established scores iHS, XP-EHH, and Rsb can be used without the requirement for sequences to be phased and for variants to be polarized. Although the issue of phasing can often be solved computationally and its importance is likely to wane soon because of rapid improvements in sequencing technologies, in the meantime, methods that can deal with unphased data may find their niche. In contrast, the polarization of alleles will always remain imperfect and incomplete, notwithstanding rare cases of available ancient DNA. This is true even more for cases of “reticulate” evolution such as hybridization/admixture, where the very concept of an ancestral allele gets blurred. Accordingly, we expect any method able to handle unpolarized variants to remain a useful complement to methods that cannot.

We compared the different approaches to detection of selective sweeps by the FDR and their statistical power. We would like to emphasize the importance of the former, because typically, in whole-genome scans, only a handful of most extreme “outlier” regions can be investigated in detail further, and it is more important to identify them correctly than to know the overall level of selection as would be described by statistical power. We even want to caution readers that reporting large numbers of putative selective sweeps may inadvertently be suggestive of a precision level that cannot be warranted. The fine-scale plots of our candidate regions in Figs 16–19 of S2 Text should serve as a reminder that their delineation depends on various often overlooked parameters such as the handling of gaps and boundary regions, the clustering of significant scores, and not the least, the thresholds applied, which are notoriously uncertain given that in many cases, null-models can be specified only roughly.

The selection parameters we implemented in the simulations were inspired by the human *LCT* locus, where a single dominant allele is generally believed to have undergone long-term strong selection in Europeans. Dealing with such variants should be an easy task for all the methods we investigated; however, we do not claim that they represent a typical or even major mode of biological evolution. Likewise, we are well aware that nontrivial demographic characteristics can have a decisive impact on the FDR and power of statistical tests of neutrality [33, 58]. On the other hand, we do not expect them to overturn our qualitative claims about relative importance of phase or ancestry information.

Our simulations revealed that SFS-based methods, constructed for the detection of sweeps near completion, are unable to detect ongoing sweeps when the selected variant still has an intermediate frequency. Polarization is more important for these methods than for EHH-based ones, yet, unexpectedly, sample size is not (at least in the range investigated).

Concerning EHH-based statistics, we demonstrated that although omission of ancestry information entails a substantial decrease in peak values, the conspicuous absence of low scores can still be exploited to delineate candidate regions. In contrast, the claims of some authors, [20, 30], that the phase can be ignored without a major loss of information must be regarded as too optimistic. The main reason is that in this case, the estimation of the statistics relies solely on individuals that are homozygous at the respective focal markers. This drawback is less of a problem for EHHS because under Hardy–Weinberg proportions, more than half of individuals in a population can be expected to be homozygous for a given marker. Consequently, in a sample of 100 chromosomes, typically ∼50 chromosomes are suitable for calculating EHHS and the derived XP-EHH and Rsb. This seems enough to obtain substantial similarity with their homologs for phased data as Table 3 shows for empirical data. For iHS, however, EHH has to be estimated for each allele independently, and this approach often renders the estimation for the minor allele unreliable because few sequences can be utilized.

To increase the robustness of estimation in unphased data, we chose 10 as the minimum number of sequences to be available for estimation at the focal site. Nonetheless, the depletion of variants with intermediate frequency is a major hallmark of a selective sweep near completion [9, 10]; hence, for iHS, this seemingly mild condition can entail the exclusion of many markers around the selected site because few individuals will be homozygous for the corresponding minor alleles. This pattern is most obvious at the LCT locus (Fig 7) but seems to be a general phenomenon (Figs 16–19 of S2 Text). Furthermore, we increased the cutoff for EHH/EHHS integration from 0.05 to 0.1 and stopped integration too when only a single homozygous individual (a single shared haplotype) remained. These added restrictions are aimed at preventing a single (or very few) shared haplotype(s) with extreme length to cause high scores only by chance. Nevertheless, as Fig 4 indicates, the improvement is moderate. The authors of both [20, 30] have invented more sophisticated metrics: the former did not integrate EHH but rather adjusted a logistic function describing its decay (actually an increase in $\frac{1}{2}$ (1–EHH)) with increasing distance from the focal marker. The latter research group repeated a whole-genome scan 50 times in a bootstrapped sample to eliminate the most volatile 50% of significant markers. We doubt, however, that any such noise reduction can overcome the general problem of an insufficient number of exploitable sequences.

Therefore, the extremely uneven length of shared haplotypes under neutrality like the one seen in Fig 2 produces difficult-to-handle background noise. Were this length log-normally distributed as suggested by the right-hand panels of the figure, then the remedy would be to replace the arithmetic average in Eq 3 by a geometric one. We briefly probed such a replacement but recognized that the cutoff parameters are more important than the type of averaging. Indeed, the authors of [50] have concluded via coalescent-based reasoning that this problem cannot have an “optimal” solution because the expected length of shared haplotypes is infinite. Accordingly, we do not expect that our *ad hoc* cutoff rules can be substantially improved or even justified by theory.

To summarize, without phasing information, selective sweeps can be located by iHS only in very large samples. Even under the idealized conditions of our simulations, at least 200 sequences are necessary to detect sweeps where the selected variant reaches an intermediate frequency, whereas for sweeps in later stages, the sample size should exceed 400 sequences. Consequently, phasing should be performed whenever possible. The poor overlap of inferred regions when iHS is used with and without the phase in empirical data (Table 2) confirms this conclusion. The required sample sizes may be lower in partially self-crossing species, where more individuals are expected to be homozygous. Some investigators [59] have reasoned that EHH-based statistics “should be robust to any levels of selfing,” yet we want to caution the reader that these statistics presuppose a certain number of detectable recombination events to be meaningful. This is an active field of research on its own [60] and is beyond the scope of our study.

## Supporting information

S1 Text Supporting information on SFS-based methods. (PDF)

S2 Text Supporting figures and tables. (PDF)

S1 Protocol Supporting information on software and technical details. (PDF)
