# Supplementary material for: Detecting selection using extended haplotype homozygosity (EHH)-based statistics in unphased or unpolarized data
Source: PLoS One. 2022 Jan 18;17(1):e0262024. doi: 10.1371/journal.pone.0262024 (PMC8765611; doi:10.1371/journal.pone.0262024)
Supplement: S1 Text — (PDF) [file pone.0262024.s001.pdf]

S1 text. Supporting Information on
Site Frequency Spectrum-based methods

for

Detecting selection using extended haplotype homozygosity (EHH)-based statistics in unphased or unpolarized data

A. Klassmann and M. Gautier

1 | TAJIMA'S D **AND** FAY & WU'S H

The *site frequency spectrum (SFS)* is a way to describe the structure of variation seen in a sample of aligned sequences. We consider here only bi-allelic polymorphisms, which in practice most often represent SNPs. Let n refer to the sample size and S to the number of polymorphisms or *(segregating) sites*. We denote by x_j the number (i.e. the absolute frequency) of sequences carrying the derived allele at site s_j. The spectrum records the number of sites with a derived allele frequency of $i = 1, \ldots, n-1$, respectively: $\xi_i = \#\{s_j | x_j = i\}$.

In Figure 1 we have a sample of 8 sequences containing 11 polymorphic sites. At five sites the derived variant occurs only on a single sequence, at three sites on two sequences, twice on three sequences and once on 4 sequences. A famous result in population genetics, proved in at least three independent ways [1–3], is the expected frequency

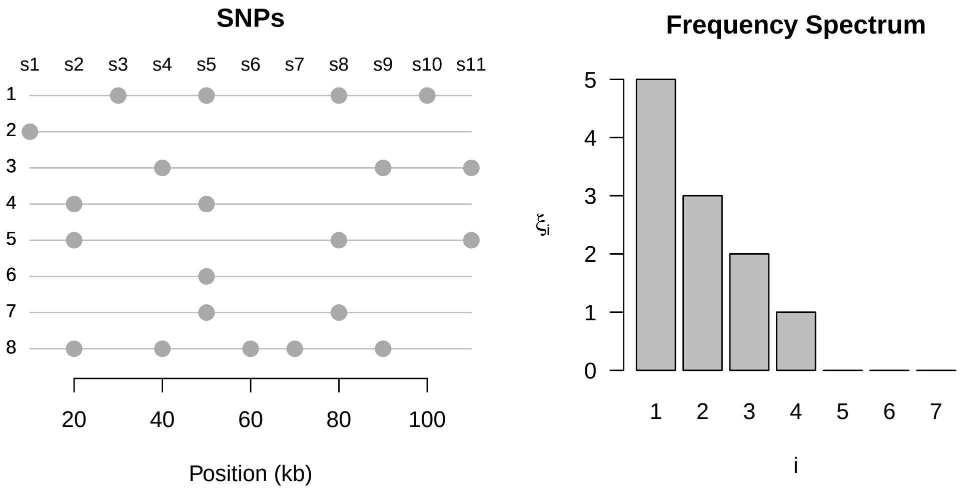

FIG 1 Pattern of variation and associated site frequency spectrum seen in a neutrally evolving genetic region. The lines symbolize sequences and $s_1, \ldots, s_{11}$ represent SNPs. For each polymorphism the derived allele is marked by a point while the ancestral variant is not shown

spectrum for a sample of sequences from a neutrally evolving homogenous population of constant size:

$$E[\xi_i] = \frac{\theta}{i} \ . \tag{1}$$

θ is formally defined as the *population-scaled mutation rate*, but for our purposes it suffices to known that it is a parameter measuring in some way the overall amount of variation in a population. Figure 2 shows the expected site frequency spectrum for the sample of Figure 1.

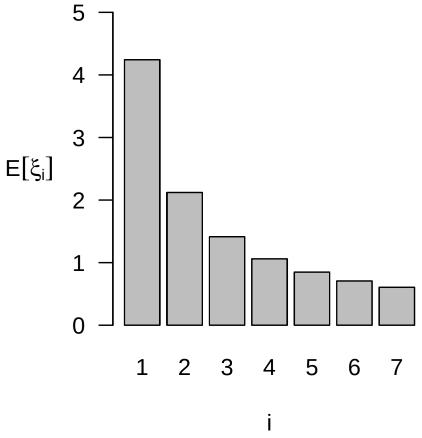

FIG 2 The expected site frequency spectrum under the null hypothesis of neutral evolution for a sample of $n = 8$ sequences and $S = 11$ polymorphic sites (which entails $\theta = 4.2$)

Samples with a frequency spectrum deviating from the expected one indicate a violation of the model assumptions. In principle, standard statistical tests such as χ^2 or *Wald* could be applied to detect such deviations. However, in our context only systematic changes that affect the global shape of the frequency spectrum are of interest; thus the need for more specialized measures.

Equation (1) can be used to estimate the usually unknown population parameter θ from a sample. Indeed, it allows to define $n - 1$ different estimators: $\hat{\theta}_i = \xi_i \cdot i$. For instance, for Figure 1 we have $\hat{\theta}_1 = 5$, $\hat{\theta}_2 = 6$, $\hat{\theta}_3 = 6$, $\hat{\theta}_4 = 4$ and $\hat{\theta}_5 = \hat{\theta}_6 = \hat{\theta}_7 = 0$. Let $\Omega_1, \ldots, \Omega_{n-1}$ be any numbers that fulfill the condition $\sum_{i=1}^{n-1} \Omega_i = 0$. We consider the linear combination of the θ-estimators $\sum_{i=1}^{n-1} \hat{\theta}_i \Omega_i$. Its expected value is zero, because $E[\sum_{i=1}^{n-1} \hat{\theta}_i \Omega_i] = \theta \sum_{i=1}^{n-1} \Omega_i = 0$ and any observed non-zero value indicates a deviation from the neutral spectrum. Although originally developed differently, Tajima's D [4], Fay& Wu's H [5, 6] and further tests [6, 7] adhere to this combination of estimators, normalized by its (estimated) standard deviation [8, 9]:

$$T = \frac{\sum_{i=1}^{n-1} \xi_i i \Omega_i}{\sqrt{\hat{var}[\sum_{i=1}^{n-1} \xi_i i \Omega_i]}} \ . \tag{2}$$

The coefficients $i\Omega_i$ which define and distinguish these tests are depicted in Figure 3. We can infer that Tajima's D yields negative values, if, in comparison to the expected neutral spectrum, more derived variants are observed

with low or high frequency and less with middle frequency. If, instead, the observed variants have predominantly intermediate frequencies, the value gets positive. Fay & Wu's H puts most weight on the highest frequencies and gets negative when these are overrepresented in a sample. For the data set of Figure 1 the tests yield $D \approx -0.08$ and $H \approx 0.99$, respectively. Are these values significantly different from zero? To answer this question we need the distributions under the null hypothesis. Although an analytical approximation for the distribution of Tajima's D has been proposed by [4], in practice distributions for both tests are obtained by simulations (which may incorporate more complex null hypotheses). Figure 4 shows the values calculated for the example of Figure 1 as gray lines within the respective distributions. The value of Tajima's D is obviously not significant while Fay & Wu's H might be marginally so. A high value of the latter statistic, however, is not a signal for *positive* selection as we shall see in the next example.

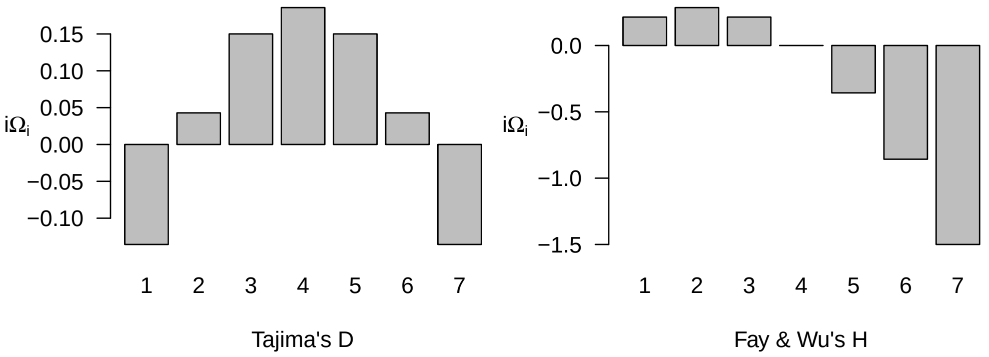

FIG 3 The coefficients $i\Omega_i$ which define the two tests by Equation (2) for sample size $n = 8$

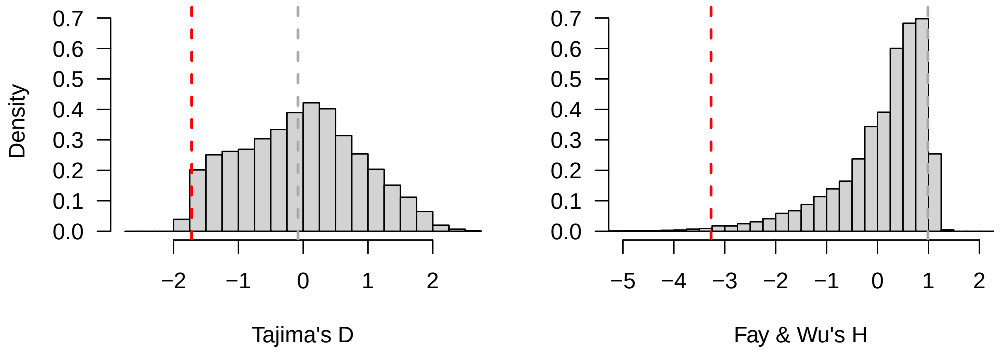

FIG 4 Distributions under neutrality obtained from 10^5 coalescent simulations using ms [10] with sample size $n = 8$ and $\theta = 4.2$. The dashed lines indicate the values obtained for the spectra of Figure 1 (gray) and Figure 5 (red), respectively

We consider here only the widely known simple model of positive selection in which an advantageous new variant replaces existing variation in a *selective sweep* [11]. Figure 5 depicts an almost completed sweep in a region with little to

no recombination. The sweep is assumed to be "instantaneous" in the sense that it proceeds so fast that the probability of new mutations arising during the sweep can be neglected. The hallmarks of the corresponding frequency spectrum are an over-abundance of high-frequency variants and a scarcity of middle frequency variants [5]. The two tests yield $D \approx -1.72$ and $H \approx -3.27$, respectively. Using the simulated null distributions, one can check that both yield a p-value < 0.01 (red lines in Figure 4).

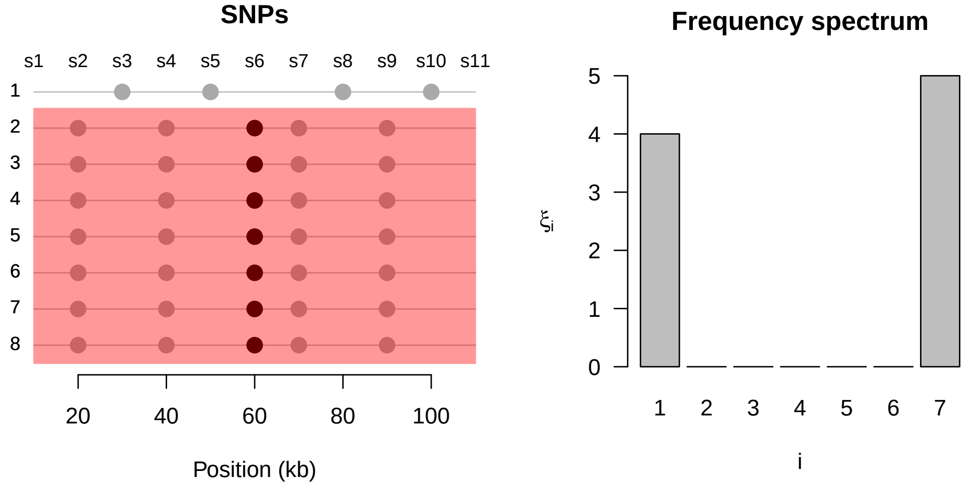

FIG 5 Pattern of variation and site frequency spectrum in a non-recombining region with a nearly completed selective sweep. We suppose that the derived variant of site *s6* in Figure 1 turned out to be strongly advantageous and reached a high population frequency in a short time. Recombination is assumed to be so low that the neutral sites which happened to be on the same sequence "hitch-hiked" along with it. The red area symbolizes the part of the pattern affected by the sweep

The two tests are easy and fast to calculate, but have several caveats: Tajima's D is highly vulnerable to the confounding effect of a growing population [12,13]; indeed, selection causes the growth of the subpopulation containing the advantageous allele and hence yields a similar signal. Even worse, [14] showed that any given frequency spectrum can be approximated by postulating demographic events alone, although these events might not be realistic. Hidden population structure affects both tests [13,15]. Clearly, the pattern in Figure 5 can arise from two genetically distinct subpopulations, too. In fact, the sweep creates a subpopulation that is at least at that locus genetically different from the remainder.

An entirely different problem is posed by recombination. It causes the variance to be over-estimated by the denominator of Equation (2), leading to smaller absolute values of the statistics [9]. Hence, the length of intervals used for the calculation of these tests needs to be small enough to limit the effect of recombination and large enough to contain a sufficient number of sites to yield statistical power.

[16] may serve as an example of a whole-genome scan (performed on human SNP data) using Tajima's D and Fay & Wu's H.

2 | SWEEPFINDER/SWEED

The test statistic calculated by SweepFinder and SweeD can be considered a refinement of Fay & Wu's H. First, [5] proposed a continuous approximation of the neutral frequency spectrum to derive a rough quantification of its distortion by a selective sweep in a region with low but non-zero recombination. Then, [17] constructed a composite likelihood test allowing for the gradual reduction of this distortion with increasing distance from the selected site. Finally, [18] provided a more detailed account for the distortion with help of coalescent theory and replaced the theoretical neutral spectrum by the empirical whole-genome spectrum. They implemented the test in the program SweepFinder. [19] provided an alternative, faster implementation in the program SweeD and added the possibility to specify simple demographic scenarios from which a null-hypothesis spectrum can be calculated analytically. Version 2 of SweepFinder includes the option to allow for background (purifying) selection [20].

The approach compares the likelihood that an observed spectrum can be regarded as neutral with the alternative of conforming to a selective sweep model. The neutral spectrum for a sample of size n is assumed to be uniform across loci with expected site frequencies $p_{i,n}$. By default, the programs take the genome-wide observed frequencies as the null spectrum, but it is possible to specify it explicitly e.g. by normalizing Equation (1):

$$p_{i,n} = \frac{1}{i} \frac{1}{\sum_k^{n-1} \frac{1}{k}} \ . \tag{3}$$

We go now through the derivation of the alternative spectrum. It starts with the assumption that the selected allele has just reached fixation in the population. Furthermore, recombination is uniform enough that the proportion of sequences remaining linked to the selected site decreases exponentially with increasing distance from the selected site. This entails that no neutral site has yet "hitch-hiked" to fixation in the population, although in a sample they might turn out monomorphic. Figure 6 represents such a scenario just before fixation of the selected variant.

To be precise, at a distance d from the selected site, the probability that an arbitrarily chosen sequence from the population has become unlinked ("escaped away") from the sequence with the selected site is given by

$$P_e = 1 - e^{-\alpha d} \ . \tag{4}$$

The coefficient α is assumed to represent a combined measure for recombination rate and (inverse) "strength" of selection [21]. For a sample of n sequences, the probability that k of them have escaped the sweep is obtained by the binomial distribution:

$$P_e(k) = \binom{n}{k} P_e^k (1 - P_e)^{n-k} \ . \tag{5}$$

Now we calculate the expected site frequency spectrum for the sample at a position where k sequences escaped and $n-k$ are still linked to the selected site. We assume that the population evolved conform to the null model before the sweep, and the sweep occurred "instantaneously" just before sampling, shifting the frequency of a possible neutral variant at that position. Figure 7 shows a hypothetical sample genealogy with an observed derived allele frequency of $i = 4$ like at site $s9$ in Figure 6. We assume that $k = 5$ lineages "escaped" the sweep and one lineage, carrying the variant, was expanded to $n - k = 3$, replacing $n - k - 1 = 2$ other lineages. In the depicted genealogy, the variant was present in 3 lineages before the sweep and the corresponding probability is given by the null spectrum $p_{3,8}$. Slightly more general, if we know that $k - 1$ lineages go extinct and the advantageous new mutation is linked to our variant,

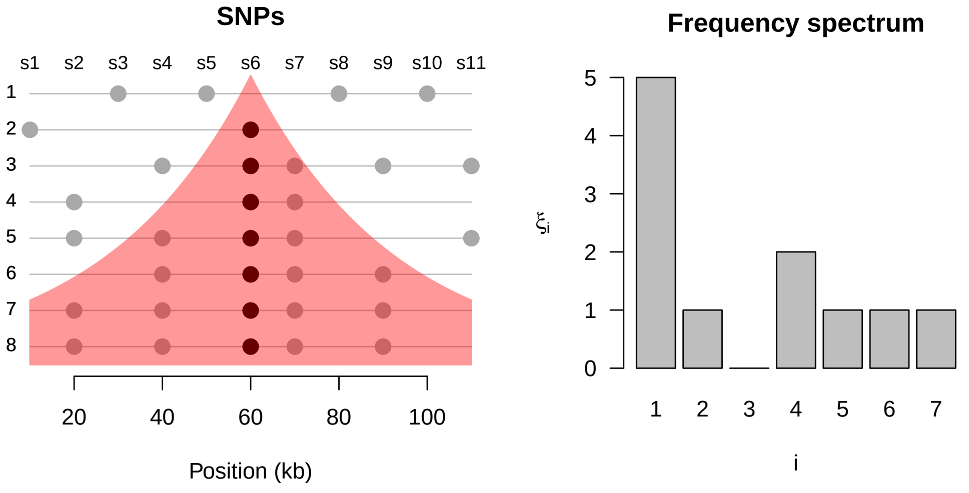

FIG 6 Pattern of variation and site frequency spectrum in a recombining region with a nearly completed selective sweep. As in the previous scenario, we assume that the derived variant of *s*6 caused a selective sweep. In contrast to Figure 5, recombination entails that with increasing distance to the selected site ever fewer neutral sites remain linked and can hitch-hike. The red area symbolizes the part of the pattern affected by the sweep. Its upper boundary is defined by the curve $e^{-\alpha d}$ with $\alpha = 3 \cdot 10^{-5}$ and d the distance to site *s*6. Note that despite the strong pattern seen in the sequences, the frequency spectrum of the whole region is only moderately distorted and hence application of Tajima's D and Fay & Wu's H do not yield significant values ($D \approx -0.24$ and $H \approx -0.60$, respectively)

then the probability of obtaining a frequency of i in the observed sample of n sequences is equal to having a frequency of $i - (n - k - 1)$ in the subsample of $k + 1$ "surviving" lineages. The latter can be computed by the hypergeometric distribution on taking $k + 1$ elements out of n. If the variant has an observed frequency of $i = 4$, this can only occur (given $n - k - 1 = 2$) when it had a frequency of 2, 3 or 4 in the neutral sample prior to the sweep, hence we have the following non-zero terms:

$$p_{2,6} = p_{2,8} \cdot \text{hyper}(2, 8, 2, 6) + p_{3,8} \cdot \text{hyper}(2, 8, 3, 6) + p_{4,8} \cdot \text{hyper}(2, 8, 4, 6)\ . \tag{6}$$

In general, the probability of having j derived variants in a subsample of $k + 1$ from n sequences yields

$$p_{j,k+1} = \sum_{m=j}^{n-1} p_{m,n} \cdot \text{hyper}(j, n, m, k + 1)\ . \tag{7}$$

Note that a subsample can be monomorphic ($p_{0,k+1} \neq 0$ and/or $p_{k+1,k+1} \neq 0$) even if the original sample is not.

In Equation (8) we put things together and define the probabilities $p^*_{i,n}$ of the alternative hypothesis: the first term on the right covers the case where all lineages escaped the sweep and hence the probability of observing i derived variants is given by the expected neutral spectrum $p_{i,n}$. The first term in the sum covers the case where the selected variant arose on a sequence carrying the derived allele. The probability of this happening is $\frac{i-(n-k)+1}{k+1}$, because there must have been $i - (n - k) + 1$ lineages with the derived allele in the subsample of $k + 1$ non-extinct lineages. The

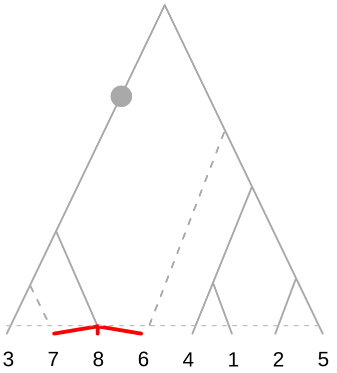

FIG 7 A hypothetical genealogy that is conform to the pattern of variation seen at site $s9$ in Figure 6 with 4 sequences carrying the derived allele. Lineages affected by the sweep (linked to the selected variant) are marked in red. Right before the sweep, the genealogy is assumed to represent a sample from a population evolving conform to the null model. 5 lineages “escaped” the sweep and two lineages (dashed lines) were replaced by the sweep

probability of having $i-(n-k)+1$ sequences with the derived allele in a neutral subsample of size $k+1$ is given by $p_{i-(n-k)+1,k+1}$. The second term in the sum covers the case where the sweep expands a lineage with the ancestral variant. In this case the observed i sequences with the derived variant must belong to the surviving lineages, and hence the probability that the selected variant arose on a lineage with the ancestral variant is $\frac{k+1-i}{k+1}$. The probability of obtaining i derived variants in the neutral subsample of $k+1$ lineages before the sweep is given by $p_{i,k+1}$.

$$p^*_{i,n} = P_e(n)p_{i,n} + \sum_{k=0}^{n-1} P_e(k)\left(p_{i-(n-k)+1,k+1}\frac{i-(n-k)+1}{k+1} + p_{i,k+1}\frac{k+1-i}{k+1}\right) \tag{8}$$

One can show that $\sum_{i=0}^{n} p^*_{i,n} = 1$. Note that for small d, $P_e \approx 0$ and $P_e(0) \approx 1$, meaning that most neutral variants are nearly fixed or extinct and the probabilities to yield monomorphic sites in a sample, $p^*_{0,n}$ as well as $p^*_{n,n}$, become dominant terms. If the null spectrum does not include monomorphic sites (as we assume here), these two cases must be disregarded for the alternative spectrum, too, and the remaining probabilities $p^*_{1,n}, \ldots, p^*_{n-1,n}$ be normalized.

Figure 8 shows the probabilities $p^*_{i,n}$ for three different values of d. Note that at $d = 0$ the selected variant is assumed to be fixed and the probability to observe any polymorphism is zero (to avoid this singularity, SweepFinder replaces d by $\max(d, 0.01)$). The spectrum looks similar to that of Figure 5 in the vicinity of the selected site, yet with increasing distance it approaches the neutral one of Figure 1 (or an otherwise defined null spectrum).

The composite likelihood function of the null hypothesis is defined as the product over all $j = 1, \ldots, S$ sites of the individual likelihoods of the observed number of sequences with the derived variant x_j:

$$CL_0 = \prod_{j=1}^{S} p_{x_j,n} \tag{9}$$

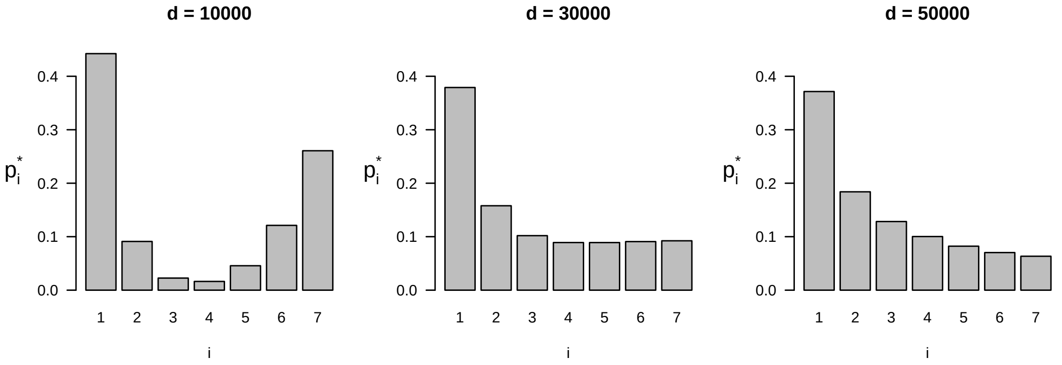

FIG 8 The probabilities under the alternative hypothesis to observe i sequences with the derived allele at a site in distance d from the selected site (for $n = 8$ and $\alpha = 3 \cdot 10^{-5}$)

For any given α the composite likelihood function of the alternative hypothesis is defined by

$$CL_A(\alpha, position) = \prod_{j=1}^{S} p^*_{x_j,n}(\alpha, d(s_j)) \,, \tag{10}$$

where $d(s_j)$ is the distance from site s_j to the tested position.

The reported result is the log ratio of both composite likelihoods, maximised over α

$$CLR(position) = \max_{\alpha} log \left(\frac{CL_A(\alpha, position)}{CL_0} \right) \,. \tag{11}$$

In practice, the CLR is computed in a region around the examined position that is bounded by a cutoff for d beyond which the effects of the sweep are neglected (SweepFinder uses $\alpha d < 12$).

Positive numbers indicate a higher likelihood for the alternative hypothesis of the presence of a selective sweep at that position. Negative numbers would favour the null hypothesis, but both programs replace them by zeros.

In order to calculate the CLR for our example of Figure 6, we first extract the derived allele frequencies and store them in a file `sweep.SF.txt`

```
position x   n   folded
10000    1   8   0
20000    4   8   0
30000    1   8   0
40000    5   8   0
50000    1   8   0
60000    7   8   0
70000    6   8   0
80000    1   8   0
90000    4   8   0
100000   1   8   0
110000   2   8   0
```

By default, both SweepFinder and SweeD use the empirical "whole-genome" site frequency spectrum as null spectrum. For our small example this is not reasonable and we use instead the expected spectrum under neutrality given by Equation (3), written into file `neutralSpec.txt` [SweeD is supposed to be able to calculate spectra analytically, however the corresponding command line parameters were not recognized by the current version (4.0.0)]:

```
0   0
1   0.3857
2   0.1928
3   0.1286
4   0.0964
5   0.0771
6   0.0643
7   0.0551
```

We specify the CLR to be calculated on a grid of 5 positions. Although in our example these coincide with positions of polymorphisms, in practice they typically do not. And they need not, because, as we remind, the alternative hypothesis states that the selected allele at the tested grid point is fixed. The command for SweepFinder yields

```
SweepFinder2 -l 5 sweep.SF.txt neutralSpec.txt sweep.SF.result
```

and produces the file `sweep.SF.result`:

```
location        LR          alpha
10000.000000    0.295646    1.442784e-04
35000.000000    0.155739    1.296432e-04
60000.000000    2.205437    6.665860e-04
85000.000000    0.002981    2.903386e-04
110000.000000   0.001821    2.741641e+02
```

The LR values show that although the alternative hypothesis is favored at each examined position, only at position 60000 is the ratio notably different from zero. Significance can only be assigned from "genome-wide" values, either from simulations or empirical. Note that the fitted parameter α alone provides little information about whether a sweep is likely to have occurred.

The command for SweeD yields

```
SweeD -grid 5 -input sweep.SF.txt -isfs neutralSpec.txt -name sweep
```

and results are stored in a file called `SweeD_Reports.sweep`:

```
Position     Likelihood      Alpha           StartPos     EndPos
10000.0000   2.702000e-01    2.400001e-04    10000.0000   50000.0000
35000.0000   1.556454e-01    1.273136e-04    10000.0000   110000.0000
60000.0000   2.205437e+00    6.858988e-04    50000.0000   70000.0000
85000.0000   2.953187e-03    2.946162e-04    50000.0000   110000.0000
110000.0000  1.819194e-03    2.763840e+02    100000.0000  110000.0000
```

We see that both tools yield very similar results. The start and end positions reported by SweeD are likely to quantify the extent of the sweep as measured by $\frac{1}{\alpha}$, but are not documented.

[22] may serve as a "real-world" example for a whole-genome scan (on human SNP data) using SweepFinder.

3 | TESTS WITHOUT POLARIZATION

What happens, if variants cannot be polarized, i.e. ancestral and derived variants cannot be distinguished? In this case, the site frequency spectrum must be *folded*, i.e. values for i and $n-i$ added together for $i = 1, \ldots, \frac{n}{2}$, respectively. Equation (2) can be modified accordingly [8]. However, Tajima's D remains unchanged as the symmetry seen in Figure 3 may already suggest. For Fay & Wu's H, in contrast, all folded weights Ω_i^{folded} are zero and the test becomes meaningless.

Both SweepFinder and SweeD accept unpolarized variants as input. The result of SweepFinder for our example data suggests that some power is lost:

```
location        LR          alpha
10000.000000    0.856797    1.419107e-04
35000.000000    0.012115    2.448181e-04
60000.000000    0.819152    6.540367e-04
85000.000000    0.035682    2.351220e-04
110000.000000   0.003708    2.320556e+02
```

SweeD at present (version 4.0.0) contains a bug (filed on 27 August 2020) when calculating CLR for folded spectra and we do not reproduce its output here.

References

[1] Kimura M. Theoretical Foundation of Population Genetics at the Molecular Level. Theoretical Population Biology. 1971;2:174–208.

[2] Fu YX. Statistical properties of segregating sites. Theoretical Population Biology. 1995;48:172–197.

[3] Hudson RR. A New Proof of the Expected Frequency Spectrum under the Standard Neutral Model. Plos One. 2015;10(7):e0118087. doi:10.1371/journal.pone.0118087.

[4] Tajima F. Statistical method for testing the neutral mutation hypothesis by DNA polymorphism. Genetics. 1989;123(3):585–95.

[5] Fay JC, Wu CI. Hitchhiking under positive Darwinian selection. Genetics. 2000;155(3):1405–13.

[6] Zeng K, Fu YX, Shi S, Wu CI. Statistical tests for detecting positive selection by utilizing high-frequency variants. Genetics. 2006;174(3):1431–9. doi:10.1534/genetics.106.061432.

[7] Fu YX, Li WH. Statistical tests of neutrality of mutations. Genetics. 1993;133(3):693–709.

[8] Achaz G. Frequency spectrum neutrality tests: one for all and all for one. Genetics. 2009;183(1):249–258. doi:10.1534/genetics.109.104042.

[9] Ferretti L, Perez-Enciso M, Ramos-Onsins SE. Optimal neutrality tests based on the frequency spectrum. Genetics. 2010;186(1):353–65. doi:10.1534/genetics.110.118570.

[10] Hudson RR. Generating samples under a Wright-Fisher neutral model of genetic variation. Bioinformatics. 2002;18(2):337–338.

[11] Stephan W. Selective Sweeps. Genetics. 2019;211(January):5–13. doi:10.1038/ncomms6281.

[12] Tajima F. The effect of change in population size on DNA polymorphism. Genetics. 1989;123(3):597–601.

[13] Simonsen KL, Churchill GA, Aquadro CF. Properties of statistical tests of neutrality for DNA polymorphism data. Genetics. 1995;141(1):413–29.

[14] Myers S, Fefferman C, Patterson N. Can one learn history from the allelic spectrum? Theoretical Population Biology. 2008;73(3):342–8. doi:10.1016/j.tpb.2008.01.001.

[15] Przeworski M. The signature of positive selection at randomly chosen loci. Genetics. 2002;160(3):1179–1189.

[16] Carlson CS, Thomas DJ, Eberle MA, Swanson JE, Livingston RJ, Rieder MJ, et al. Genomic regions exhibiting positive selection identified from dense genotype data. Genome Research. 2005;15:1553–65. doi:10.1101/gr.4326505.

[17] Kim Y, Stephan W. Detecting a local signature of genetic hitchhiking along a recombining chromosome. Genetics. 2002;160(2):765–777.

[18] Nielsen R, Williamson S, Kim Y, Nielsen R, Williamson S, Kim Y, et al. Genomic scans for selective sweeps using SNP data. Genome research. 2005;15:1566–1575. doi:10.1101/gr.4252305.

[19] Pavlidis P, Živković D, Stamatakis A, Alachiotis N. SweeD: likelihood-based detection of selective sweeps in thousands of genomes. Molecular Biology and Evolution. 2013;30(9):2224–34. doi:10.1093/molbev/mst112.

[20] Huber CD, DeGiorgio M, Hellmann I, Nielsen R. Detecting recent selective sweeps while controlling for mutation rate and background selection. Molecular Ecology. 2016;25(1):142–156. doi:10.1111/mec.13351.

[21] Durrett R, Schweinsberg J. Approximating selective sweeps. Theoretical Population Biology. 2004;66(2):129–38. doi:10.1016/j.tpb.2004.04.002.

[22] Williamson SH, Hubisz MJ, Clark AG, Payseur BA, Bustamante CD, Nielsen R. Localizing recent adaptive evolution in the human genome. PLoS Genetics. 2007;.
