# Supplementary material for: Detecting selection using extended haplotype homozygosity (EHH)-based statistics in unphased or unpolarized data
Source: PLoS One. 2022 Jan 18;17(1):e0262024. doi: 10.1371/journal.pone.0262024 (PMC8765611; doi:10.1371/journal.pone.0262024)
Supplement: S2 Text — (PDF) [file pone.0262024.s002.pdf]

S3 text. Supporting Figures and Tables

for

Detecting selection using extended haplotype homozygosity (EHH)-based statistics in unphased or unpolarized data

A. Klassmann and M. Gautier

List of Figures

1 Unstandardized *iHS* in dependence of the derived allele frequency for simulated data 2
2 Unstandardized *XP-EHH* in dependence of the derived allele frequency for simulated data 3
3 Unstandardized *Rsb* in dependence of the derived allele frequency for simulated data 3
4 Distribution and Q–Q plots of *iHS* values for simulated data 4
5 Distribution and Q–Q plots of *iHS* values for population `CEU` 4
6 Distribution and Q–Q plots of *XP-EHH* and *Rsb* values for simulated data 5
7 Distribution and Q-Q plots of *XP-EHH* and *Rsb* values for population `CEU` vs `YRI` 5
8 Statistics around the selected site with frequency 0.5 on simulated chromosome 1 6
9 Statistics around the selected site with frequency 0.7 on simulated chromosome 1 6
10 Statistics around the selected site with frequency 0.9 on simulated chromosome 1 6
11 Statistics around the selected site after fixation on simulated chromosome 1 7
12 Statistics around the selected site with frequency 0.5 on simulated chromosome 2 8
13 Statistics around the selected site with frequency 0.7 on simulated chromosome 2 8
14 Statistics around the selected site with frequency 0.9 on simulated chromosome 2 8
15 Statistics around the selected site after fixation on simulated chromosome 2 9
16 Candidate regions using *iHS* for population `CEU` 10
17 Candidate regions using *iHS* for population `CHB` 11
18 Candidate regions using *iHS* for population `JPT` 12
19 Candidate regions using *iHS* for population `YRI` 13
20 iHS values in LCT region of European and African populations 14

List of Tables

1 Candidate regions for population CEU using *iHS* on phased & polarized data 15
2 Candidate regions for population CEU using *iHS* on phased & unpolarized data 16
3 Candidate regions for population CEU using *iHS* on unphased & polarized data 17
4 Candidate regions for population CHB using *iHS* on phased & polarized data 18
5 Candidate regions for population CHB using *iHS* on phased & unpolarized data 19
6 Candidate regions for population CHB using *iHS* on unphased & polarized data 20
7 Candidate regions for population JPT using *iHS* on phased & polarized data 21
8 Candidate regions for population JPT using *iHS* on phased & unpolarized data 22
9 Candidate regions for population JPT using *iHS* on unphased & polarized data 23
10 Candidate regions for population YRI using *iHS* on phased & polarized data 24
11 Candidate regions for population YRI using *iHS* on phased & unpolarized data 25
12 Candidate regions for population YRI using *iHS* on unphased & polarized data 26
13 Candidate regions for population CEU+GBR using *iHS* on phased & polarized data 27
14 Candidate regions for population CEU+GBR using *iHS* on phased & unpolarized data 28
15 Candidate regions for population CEU+GBR using *iHS* on unphased & polarized data 29
16 Candidate regions for population CHB+CHS using *iHS* on phased & polarized data 30
17 Candidate regions for population CHB+CHS using *iHS* on phased & unpolarized data 31
18 Candidate regions for population CHB+CHS using *iHS* on unphased & polarized data 32
19 Candidate regions for population CEU vs CHB using *XP-EHH* on phased data 33
20 Candidate regions for population CEU vs CHB using *Rsb* on phased data 34
21 Candidate regions for population CEU vs CHB using *XP-EHH*/*Rsb* on unphased data 35
22 Candidate regions for population CEU vs JPT using *XP-EHH* on phased data 36
23 Candidate regions for population CEU vs JPT using *Rsb* on phased data 37
24 Candidate regions for population CEU vs JPT using *XP-EHH*/*Rsb* on unphased data 38
25 Candidate regions for population CEU vs YRI using *XP-EHH* on phased data 39
26 Candidate regions for population CEU vs YRI using *Rsb* on phased data 40
27 Candidate regions for population CEU vs YRI using *XP-EHH*/*Rsb* on unphased data 41
28 Candidate regions for population CHB vs JPT using *XP-EHH* on phased data 42
29 Candidate regions for population CHB vs JPT using *Rsb* on phased data 43
30 Candidate regions for population CHB vs JPT using *XP-EHH*/*Rsb* on unphased data 44
31 Candidate regions for population CHB vs YRI using *XP-EHH* on phased data 45
32 Candidate regions for population CHB vs YRI using *Rsb* on phased data 46
33 Candidate regions for population CHB vs YRI using *XP-EHH*/*Rsb* on unphased data 47
34 Candidate regions for population JPT vs YRI using *XP-EHH* on phased data 48
35 Candidate regions for population JPT vs YRI using *Rsb* on phased data 49
36 Candidate regions for population JPT vs YRI using *XP-EHH*/*Rsb* on unphased data 50
37 Candidate regions for population CEU+GBR vs CHB+CHS using *XP-EHH* on phased data. 51
38 Candidate regions for population CEU+GBR vs CHB+CHS using *Rsb* on phased data. 52
39 Candidate regions for population CEU+GBR vs CHB+CHS using *XP-EHH*/*Rsb* on unphased data. 53

1 | SUPPORTING FIGURES

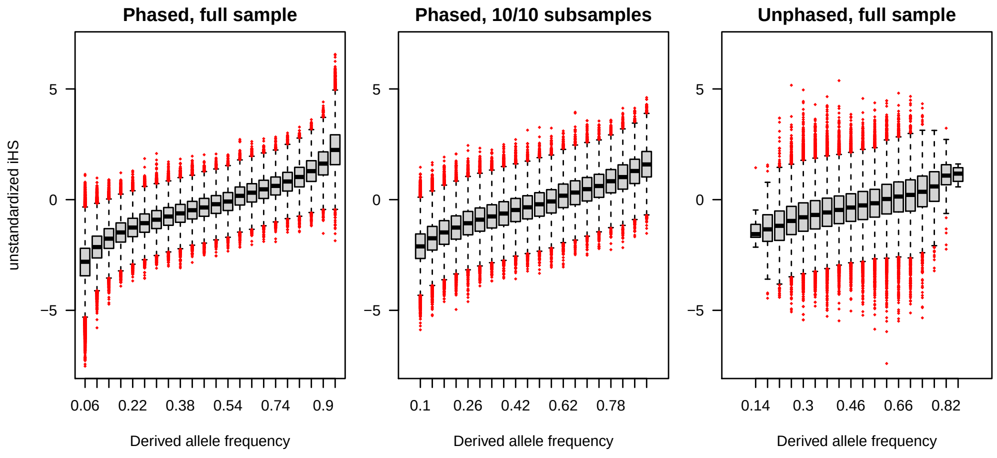

FIG 1 Boxplots of unstandardized *iHS* under neutrality for the derived allele frequencies 0.06, 0.10, ..., 0.90, 0.94 at the focal site. Marked in red are "outlier" values (defined as having a distance of more than 1.5 times the interquartile range from the first or third quartile, respectively). The left panel presents the $uniHS$ values using the original estimator. In the middle panel unstandardized iHS was calculated by taking at each focal marker a random subsample of 10 sequences carrying the ancestral and 10 sequences carrying the derived core allele. The right panel shows values obtained by using the modified estimator for unphased data as described in the main text. Simulated was a neutrally evolving chromosome of length 50 Mb with a sample size of $n = 100$. 20 simulation "runs" were used for the left and middle panel and 40 for the right panel in order to compensate for the additional requirement on focal markers that each allele has to be present on at least 5 homozygous individuals

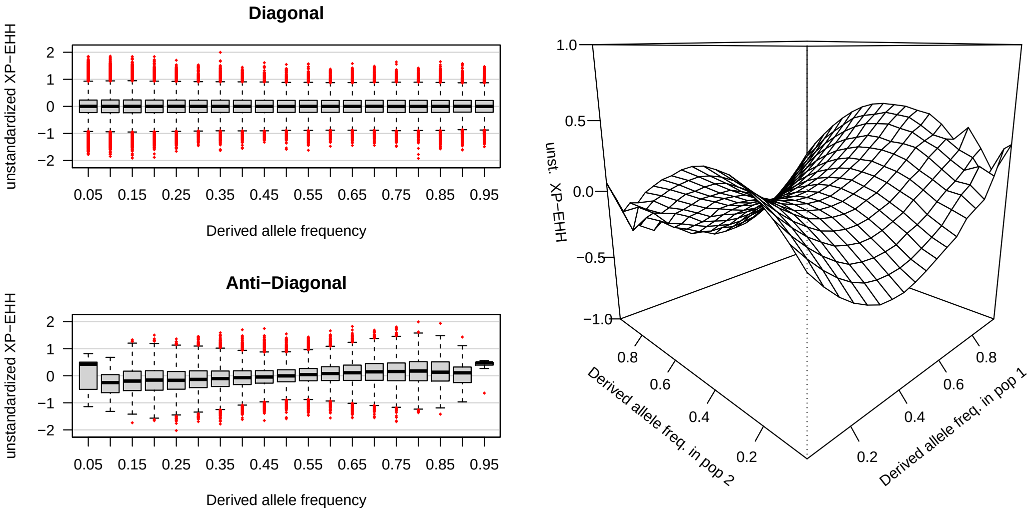

FIG 2 Unstandardized *XP-EHH* in dependence of the derived allele frequency in both populations. Simulated was a chromosome of 50 Mb evolving neutrally in two recently split populations. The sample size was $n = 100$ in each population. Results are averaged over 250 runs. In order to smooth the graph, we grouped neighbouring frequencies together, hence a data point at frequency i represents the average over frequencies $i - 0.01, i, i + 0.01$ for $i = 0.05, \ldots, 0.95$. The left panels show sections of the right panel along the diagonal ($p_s^{pop1} = p_s^{pop2}$) and antidiagonal ($p_s^{pop2} = 1 - p_s^{pop1}$). Marked in red are "outlier" values (defined as having a distance of more than 1.5 times the interquartile range from the first or third quartile, respectively)

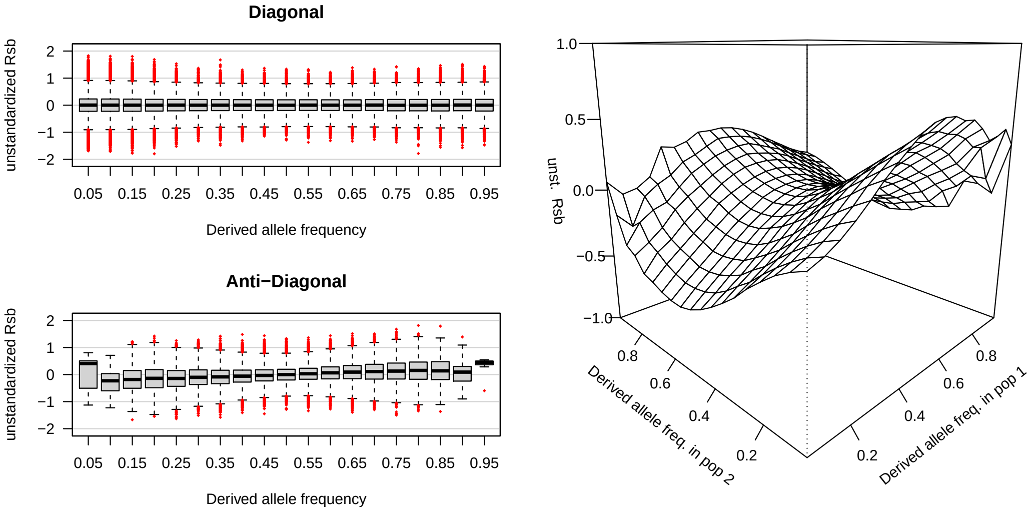

FIG 3 Same as Figure 2, but for unstandardized *Rsb*

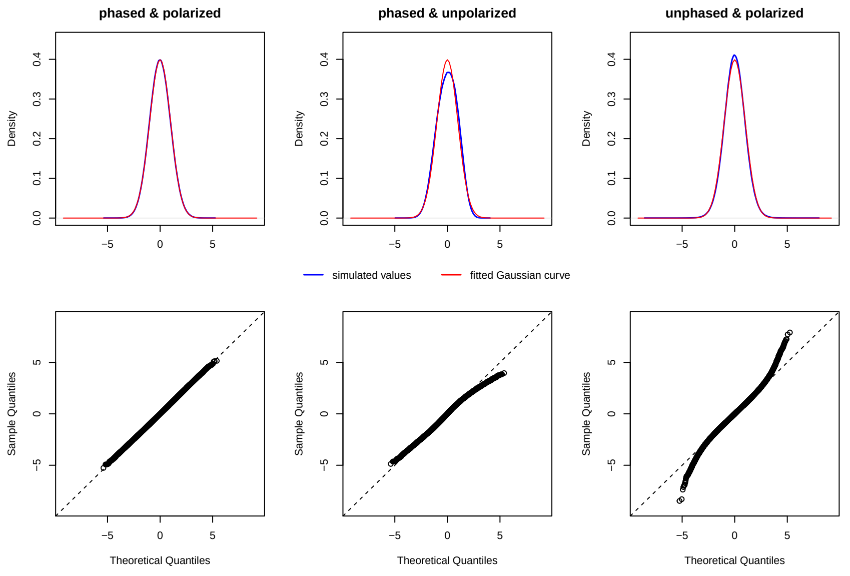

FIG 4 Distribution and Q–Q plots of the (standardized) *iHS* values from 20 simulation "runs" of a neutrally evolving chromosome of 50 Mb length with sample size $n = 200$. The left panel shows the standard statistic, the other two panels the modified versions

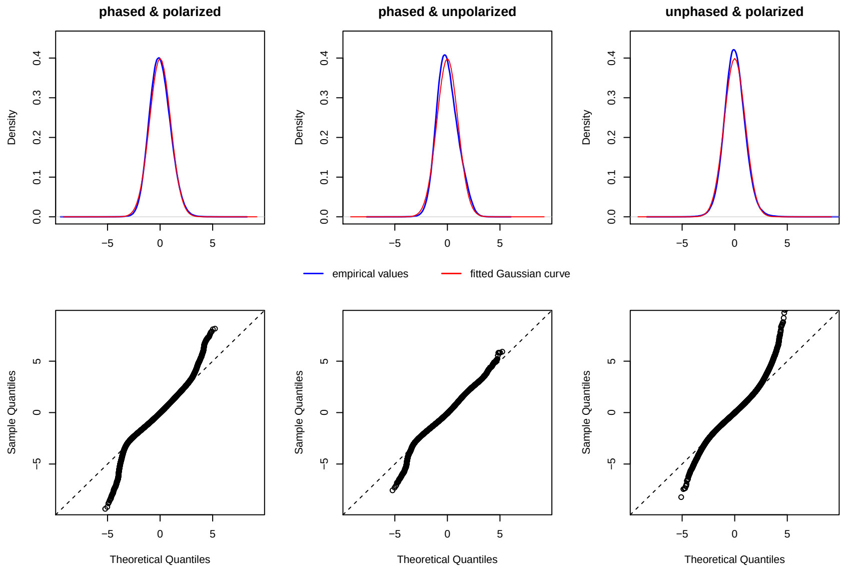

FIG 5 Distribution and Q–Q plots of the (standardized) iHS values in population CEU. Panels analogous to Figure 4

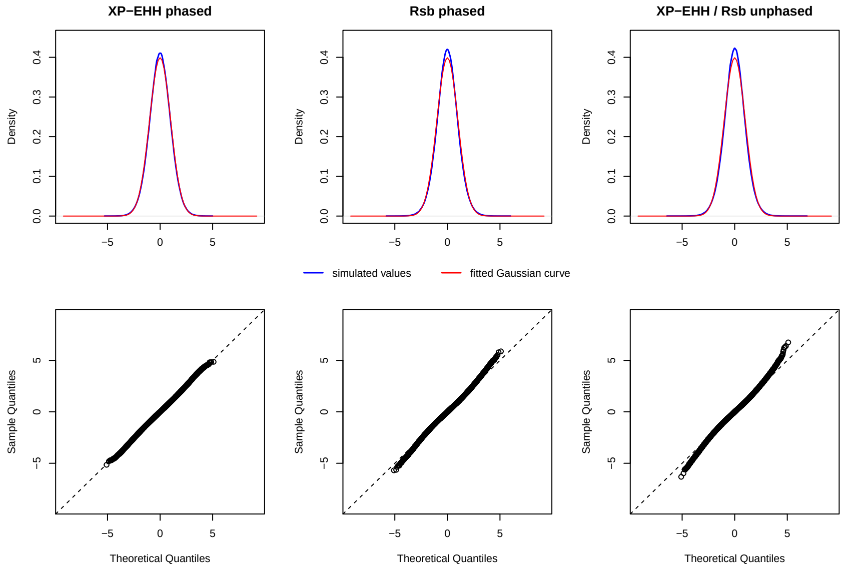

FIG 6 Distribution and Q–Q plots of the (standardized) *XP-EHH* and *Rsb* values from 20 simulation "runs" of a neutrally evolving chromosome of length 50 Mb with a sample size of $n = 200$ in each subpopulation. The two panels to the left show the standard statistics. The right panel shows *Rsb* estimated without phase information; the corresponding distribution of *XP-EHH* is virtually identical

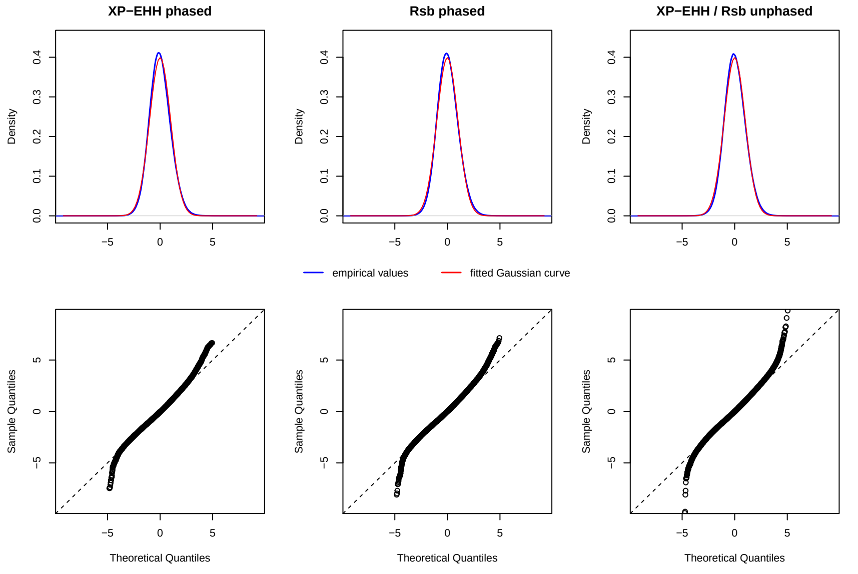

FIG 7 Distribution and Q–Q plots of the (standardized) *XP-EHH* and *Rsb* values for population `CEU` versus population `YRI`. Panels analogous to Figure 6

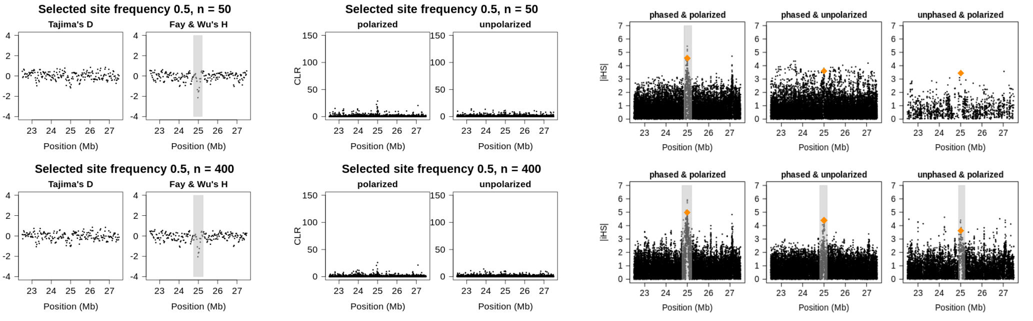

FIG 8 Statistics of the genome-wide scan on simulated data. Shown are the values around the selected site located at 25 Mb from the first simulation "run". The selected variant was required to have reached a population frequency of 50%. At the bottom, the full simulated sample of size $n = 400$ was used, on top a subsample of size $n = 50$. Delineated candidate regions are marked in gray and the *iHS* value of the selected site in dark orange. The latter is missing when the corresponding *iHS* value could not be computed

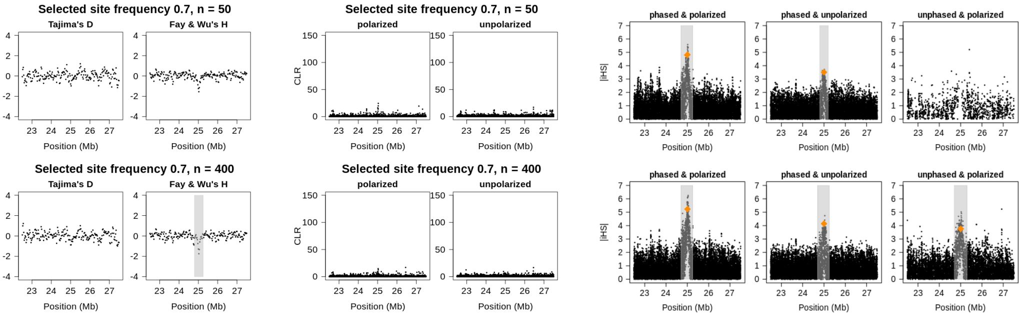

FIG 9 Same as Figure 8, but with the selected variant having reached a population frequency of 70%

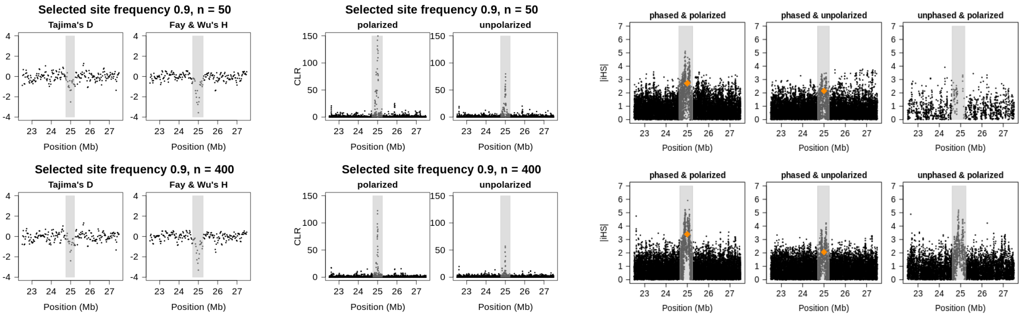

FIG 10 Same as Figure 8, but with the selected variant having reached a population frequency of 90%

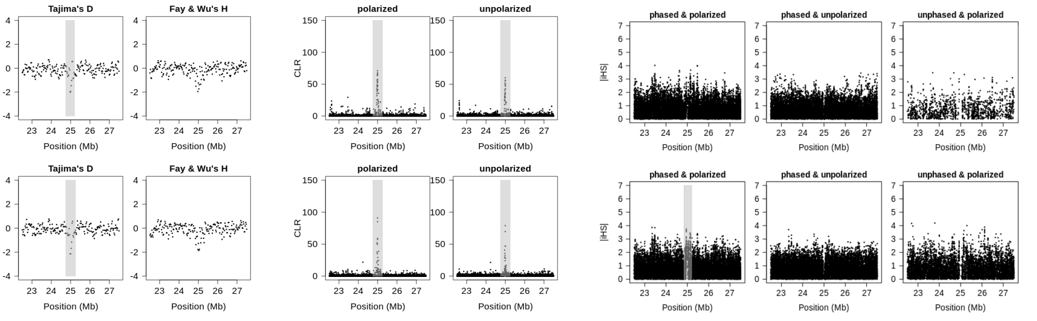

FIG 11 Same as Figure 8, but after fixation of the selected site

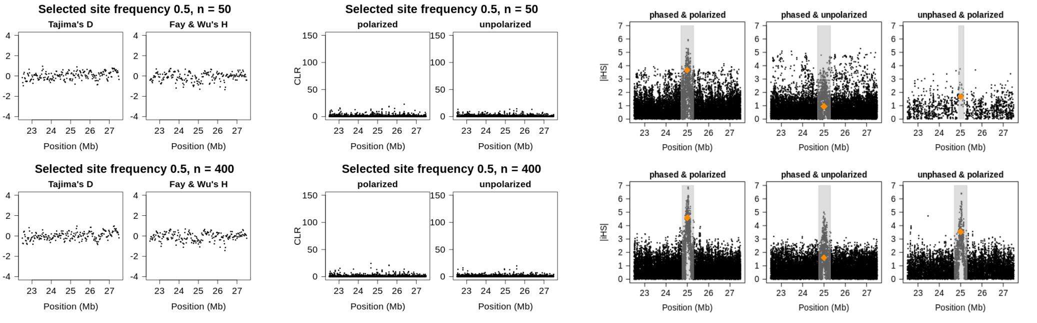

FIG 12 Same as Figure 8, but for the second simulation “run”

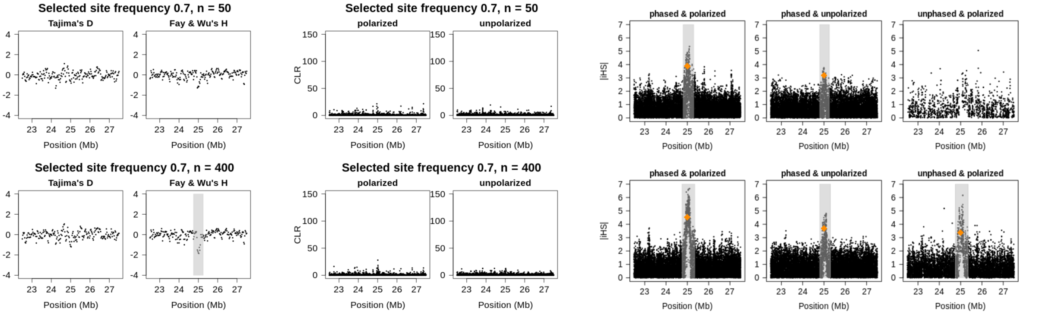

FIG 13 Same as Figure 9, but for the second simulation “run”

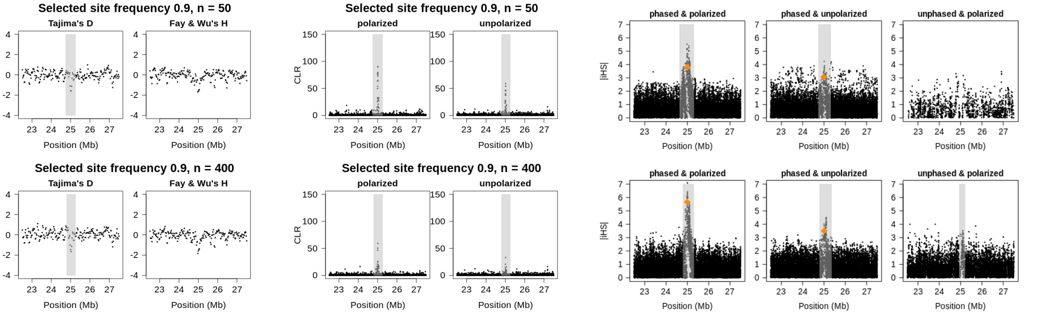

FIG 14 Same as Figure 10, but for the second simulation “run”

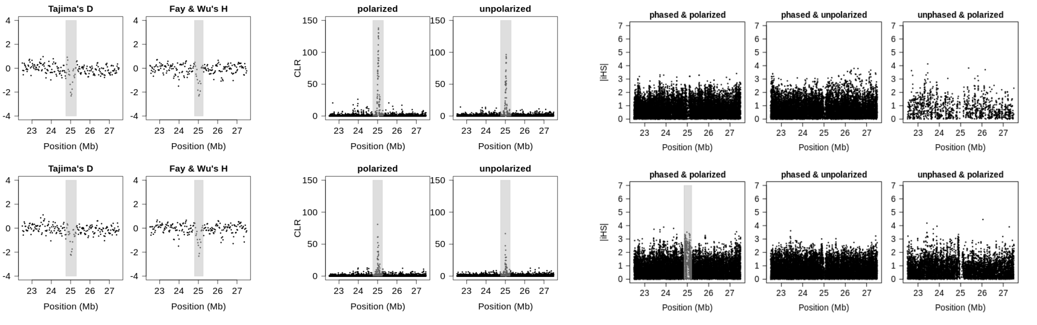

FIG 15 Same as Figure 11, but for the second simulation “run”

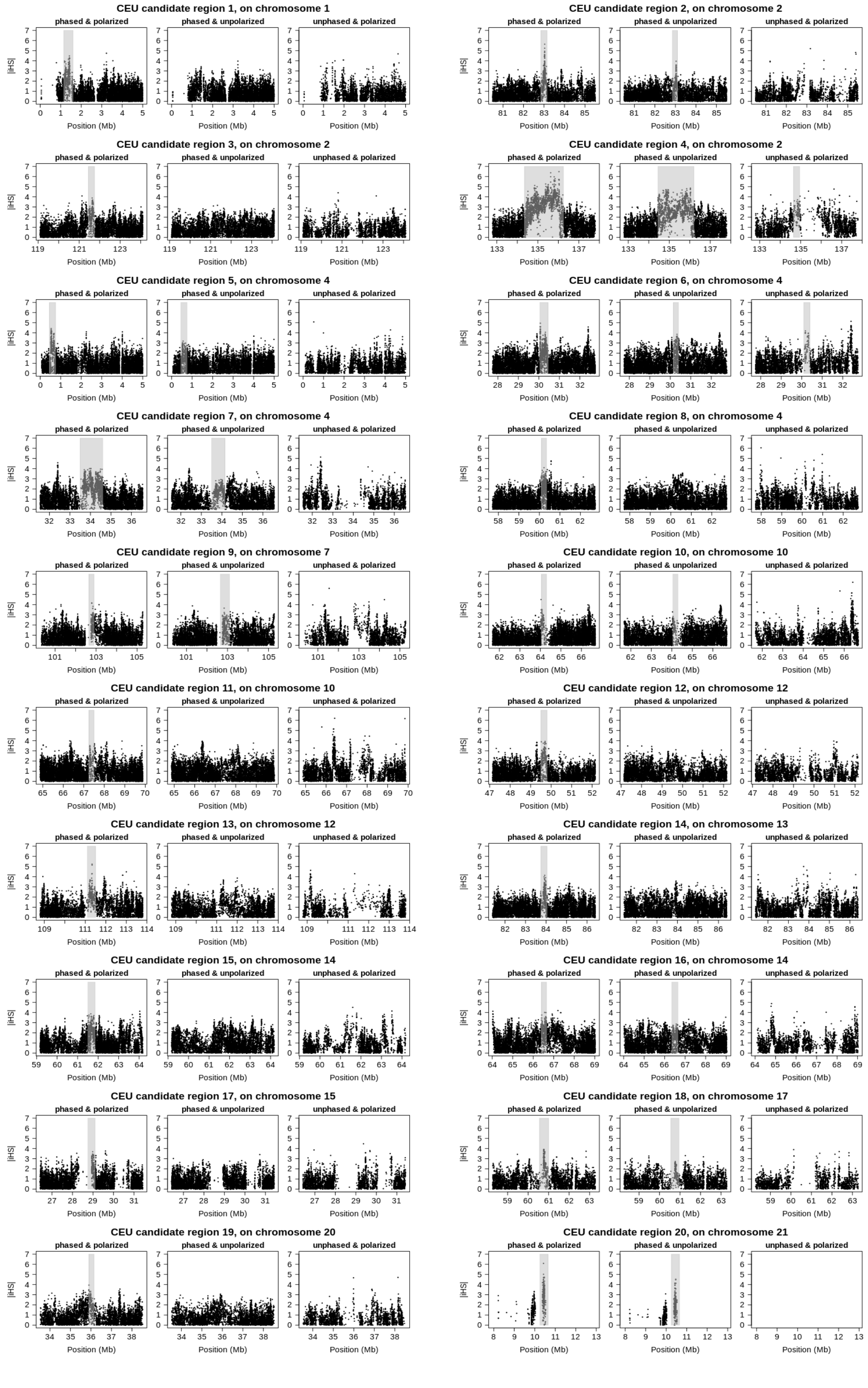

FIG 16 Candidate regions of selection for population `CEU` using *iHS*

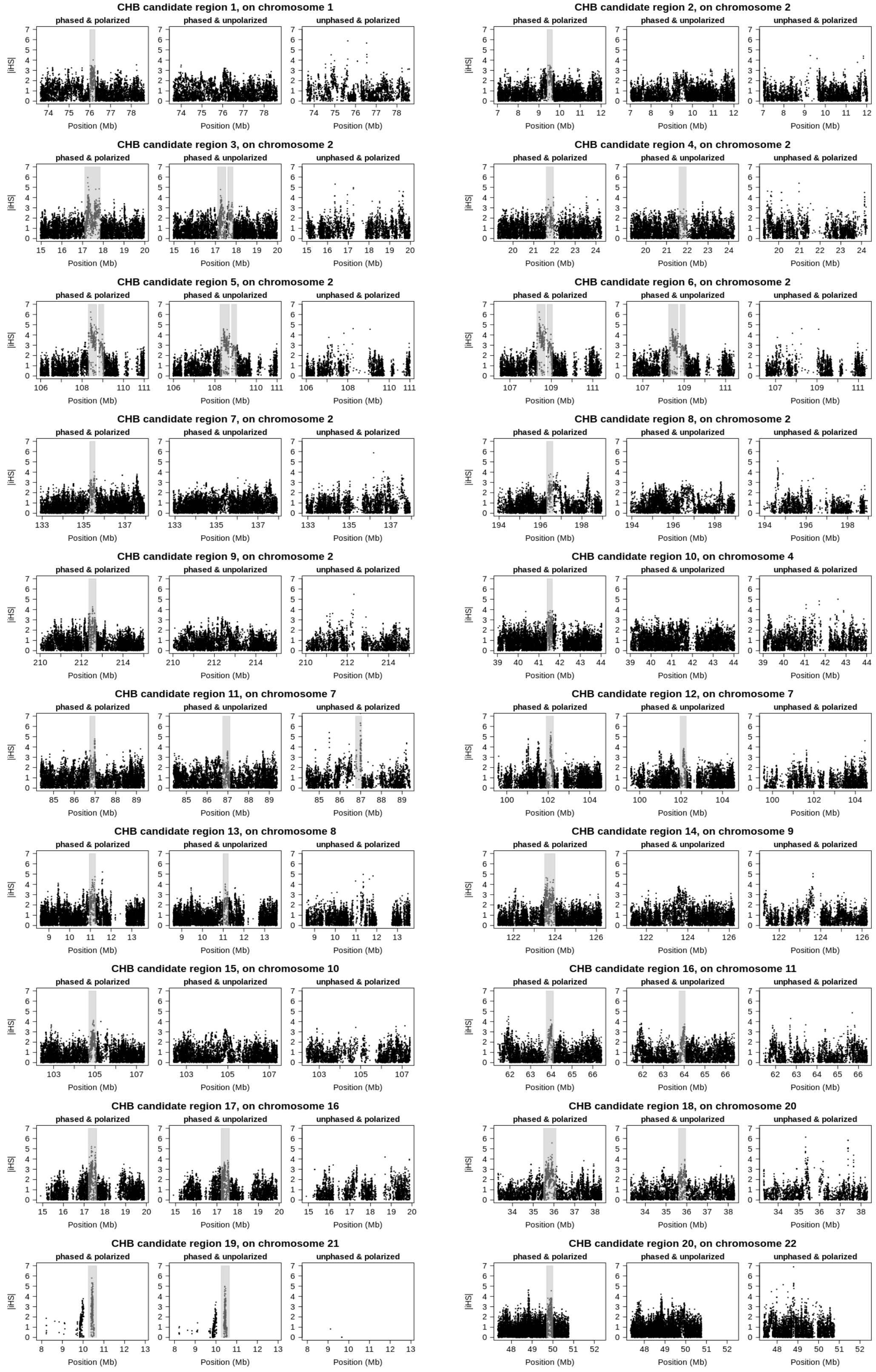

FIG 17 Candidate regions of selection for population `CHB` using *iHS*

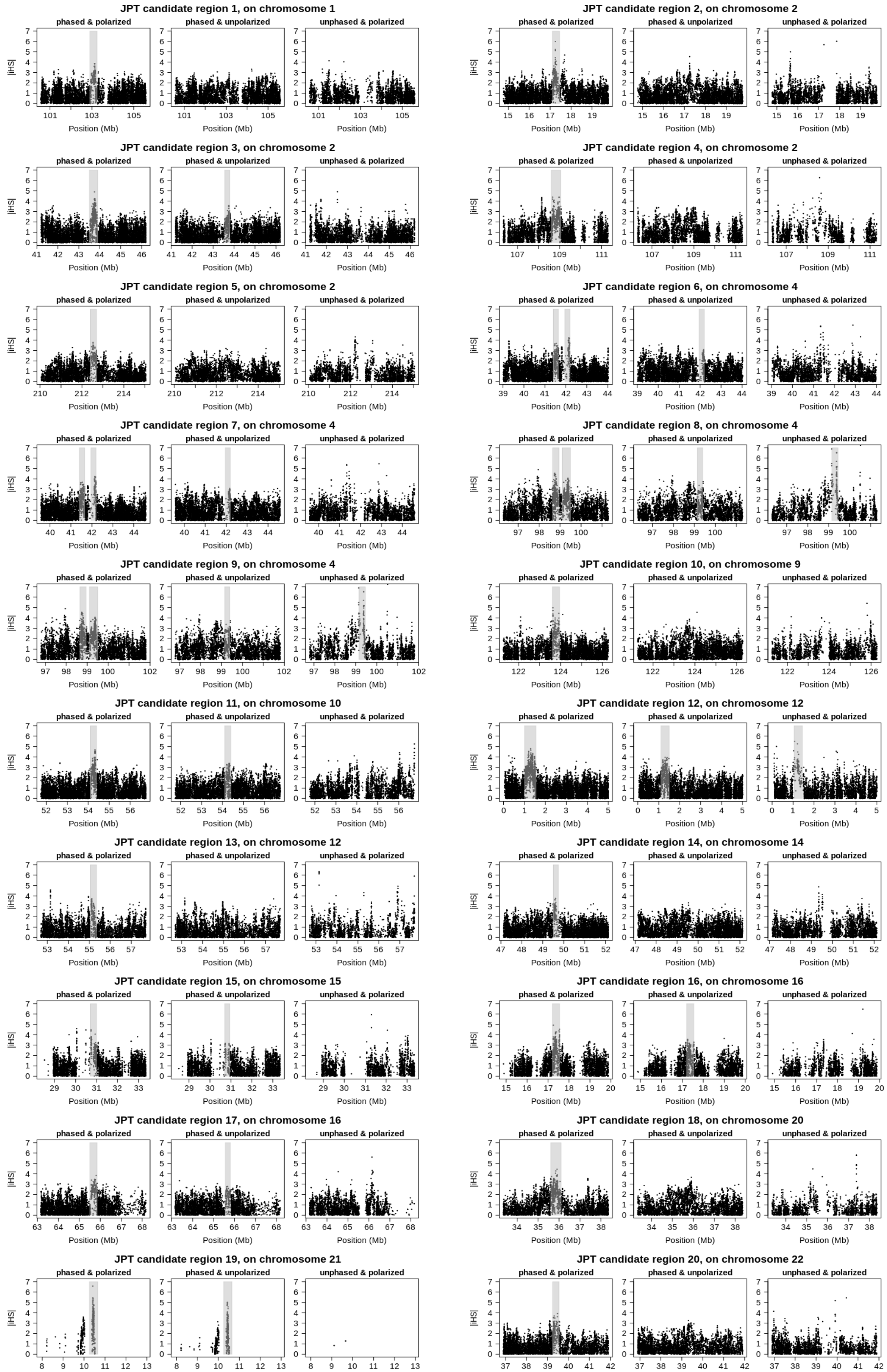

FIG 18 Candidate regions of selection for population JPT using *iHS*

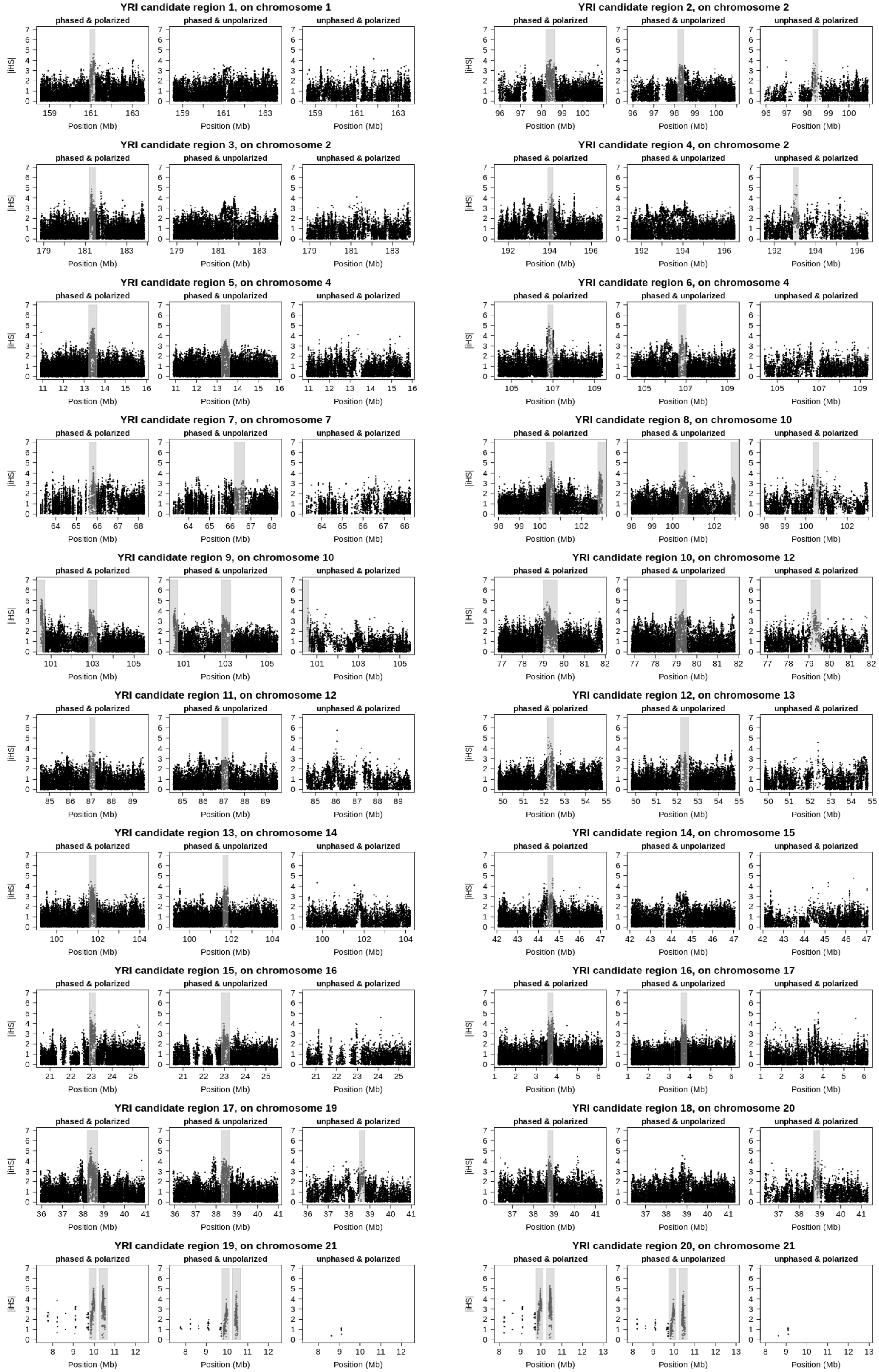

FIG 19 Candidate regions of selection for population `YRI` using *iHS*

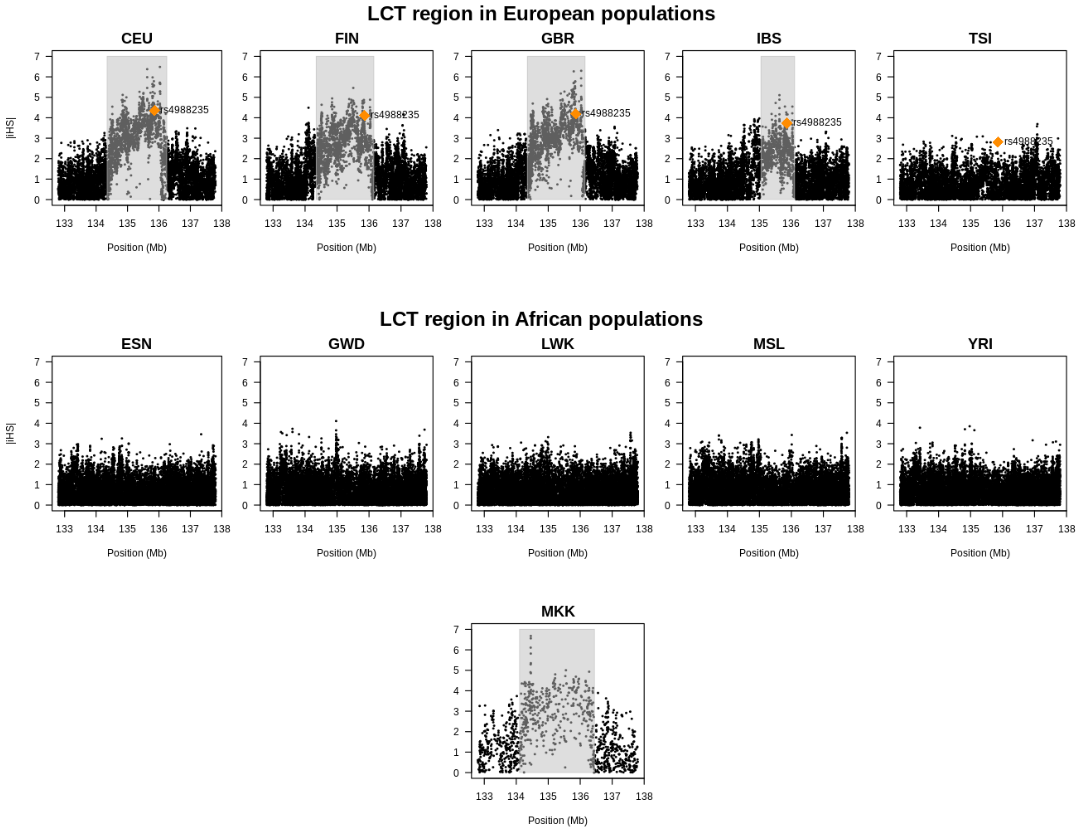

FIG 20 Absolute *iHS* values (original definition) and delineated candidate regions of selection in the *LCT* region for the European and African populations of the 1000 Genomes Project as well as for the `MKK` population of the HapMap3 project. Candidate regions have been called for each population as described in the main text; for the `MKK` sample the required minimum number of SNPs per candidate window was reduced from 150 to 20. For this sample the coordinates have been translated from Human genome assembly hg18 to hg38 before calculating *iHS*. The sharp spike is likely to be an artifact of this translation: the SNPs with most extreme values of nS_L (a score similar to *iHS*) as reported in Table 2 of (Ferrer-Admetlla et al., 2014) cover the whole region with only one located within the spike. The `MKK` sample does not contain the European lactose-persistence variant of SNP *rs4988235* and the HapMap SNPs do not comprise other variants associated with lactose-persistence, since these were identified later. See main text for references.
Abbreviations for samples not used in the main text: `FIN` Finnish in Finland; `IBS` Iberian Population in Spain; `TSI` Toscani in Italia; `ESN` Esan in Nigeria; `GWD` Gambian in Western Divisions in the Gambia; `LWK` Luhya in Webuye, Kenya; `MSL` Mende in Sierra Leone; `MKK` Maasai in Kinyawa, Kenya

2 | SUPPORTING TABLES

	CHR	START	END	LENGTH	#MRK	MEAN	MAX	#EXTR MRK	$\frac{\#EXTR\ MRK}{\#MRK}$ %
1	1	1.15	1.60	0.45	677	1.95	4.48	361	53
2	2	82.85	83.15	0.30	627	2.06	5.64	319	51
3	2	121.45	121.75	0.30	369	1.95	3.88	196	53
4	2	134.35	136.25	1.90	2334	2.90	6.48	1870	80
5	4	0.45	0.75	0.30	575	1.70	4.43	264	46
6	4	30.05	30.45	0.40	874	1.98	4.95	442	51
7	4	33.50	34.60	1.10	1718	2.06	4.01	1002	58
8	4	60.10	60.35	0.25	618	1.91	4.09	309	50
9	7	102.65	102.90	0.25	211	2.03	4.17	108	51
10	10	64.05	64.30	0.25	166	1.91	3.54	97	58
11	10	67.25	67.50	0.25	203	1.82	3.51	106	52
12	12	49.50	49.80	0.30	329	1.92	3.93	177	54
13	12	111.10	111.50	0.40	315	2.09	5.23	174	55
14	13	83.75	84.05	0.30	450	1.81	4.14	217	48
15	14	61.50	61.85	0.35	545	1.92	3.87	268	49
16	14	66.40	66.65	0.25	433	2.07	4.01	242	56
17	15	28.75	29.10	0.35	350	1.91	3.69	184	53
18	17	60.55	61.00	0.45	401	2.14	3.89	242	60
19	20	35.90	36.15	0.25	235	1.82	3.95	118	50
20	21	10.25	10.65	0.40	251	2.70	6.09	194	77

TABLE 1 Candidate regions for population `CEU` using *iHS* on phased & polarized data

	CHR	START	END	LENGTH	#MRK	MEAN	MAX	#EXTR MRK	$\frac{\#EXTR\ MRK}{\#MRK}$ %
1	1	46.90	47.15	0.25	446	1.55	3.43	233	52
2	1	185.50	185.80	0.30	440	1.53	3.04	215	49
3	2	21.35	21.60	0.25	197	1.44	2.62	110	56
4	2	82.85	83.10	0.25	544	1.63	3.96	284	52
5	2	134.45	136.20	1.75	2072	2.44	4.86	1657	80
6	3	95.30	95.65	0.35	791	1.43	2.63	369	47
7	3	103.45	103.70	0.25	832	1.47	2.91	418	50
8	4	0.45	0.75	0.30	575	1.46	3.19	264	46
9	4	30.15	30.40	0.25	466	1.66	3.86	237	51
10	4	33.50	34.15	0.65	675	1.65	2.93	364	54
11	4	102.70	103.10	0.40	549	1.53	2.74	283	52
12	7	102.65	103.10	0.45	353	1.52	3.66	166	47
13	7	139.20	139.45	0.25	336	1.51	2.37	172	51
14	10	64.05	64.30	0.25	166	1.54	2.84	84	51
15	10	82.25	82.50	0.25	377	1.65	3.30	195	52
16	14	66.35	66.65	0.30	506	1.61	2.86	296	59
17	15	48.15	48.40	0.25	210	1.55	3.29	107	51
18	17	43.05	43.30	0.25	297	1.48	2.98	153	52
19	17	60.55	60.95	0.40	382	1.66	2.72	187	49
20	21	10.25	10.65	0.40	251	1.87	4.53	150	60

TABLE 2 Candidate regions for population `CEU` using *iHS* on phased & unpolarized data

	CHR	START	END	LENGTH	#MRK	MEAN	MAX	#EXTR MRK	$\frac{\text{\#EXTR MRK}}{\text{\#MRK}}$ %
1	1	193.95	194.30	0.35	176	2.24	5.54	103	59
2	1	194.50	194.80	0.30	198	2.02	4.72	87	44
3	2	13.55	13.85	0.30	105	2.44	6.16	62	59
4	2	134.65	134.95	0.30	193	2.28	3.71	102	53
5	2	175.30	175.55	0.25	81	2.13	5.20	42	52
6	4	30.10	30.40	0.30	85	2.40	4.19	48	56
7	4	140.30	140.60	0.30	120	2.22	4.47	63	53
8	5	43.95	44.35	0.40	200	2.38	4.56	103	52
9	5	128.40	128.70	0.30	102	2.09	5.39	48	47
10	6	34.55	35.05	0.50	313	2.63	6.26	191	61
11	7	19.45	19.80	0.35	331	2.28	8.22	173	52
12	7	36.75	37.05	0.30	155	2.25	5.11	78	50
13	8	51.35	51.60	0.25	103	2.41	6.33	53	51
14	8	51.75	52.00	0.25	112	2.70	5.10	69	62
15	8	78.55	78.80	0.25	75	2.66	5.21	41	55
16	9	30.15	30.40	0.25	89	2.50	5.77	47	53
17	9	72.60	72.85	0.25	118	2.48	4.61	66	56
18	12	2.80	3.05	0.25	200	2.46	5.93	105	53
19	15	34.10	34.40	0.30	218	2.07	5.64	103	47
20	21	29.55	29.80	0.25	98	2.04	4.70	50	51

TABLE 3 Candidate regions for population `CEU` using *iHS* on unphased & polarized data

	CHR	START	END	LENGTH	#MRK	MEAN	MAX	#EXTR MRK	$\frac{\#EXTR\ MRK}{\#MRK}$ %
1	1	76.00	76.25	0.25	359	2.08	4.02	213	59
2	2	9.40	9.65	0.25	165	2.04	3.50	90	55
3	2	17.10	17.85	0.75	921	2.01	5.96	497	54
4	2	21.60	21.95	0.35	203	2.02	4.02	108	53
5	2	108.30	108.70	0.40	244	3.11	6.24	193	79
6	2	108.80	109.05	0.25	155	2.10	4.60	93	60
7	2	135.30	135.55	0.25	246	1.99	4.02	125	51
8	2	196.30	196.60	0.30	196	1.84	3.81	99	51
9	2	212.35	212.70	0.35	430	2.03	4.26	245	57
10	4	41.40	41.65	0.25	414	1.95	3.89	207	50
11	7	86.75	87.00	0.25	322	2.24	4.78	186	58
12	7	101.90	102.25	0.35	274	1.99	5.43	127	46
13	8	10.95	11.25	0.30	300	2.06	4.75	156	52
14	9	123.50	124.00	0.50	557	1.99	4.65	294	53
15	10	104.70	105.05	0.35	525	1.91	4.12	258	49
16	11	63.75	64.10	0.35	296	1.68	4.15	125	42
17	16	17.20	17.60	0.40	529	2.15	5.22	298	56
18	20	35.50	36.10	0.60	601	1.93	5.57	300	50
19	21	10.25	10.65	0.40	263	2.74	5.79	195	74
20	22	49.70	50.00	0.30	621	1.71	4.56	276	44

TABLE 4 Candidate regions for population `CHB` using *iHS* on phased & polarized data

	CHR	START	END	LENGTH	#MRK	MEAN	MAX	#EXTR MRK	$\frac{\#\text{EXTR MRK}}{\#\text{MRK}}$ %
1	2	17.10	17.50	0.40	553	1.54	4.78	274	50
2	2	17.60	17.85	0.25	205	1.70	3.61	111	54
3	2	21.60	21.95	0.35	203	1.51	2.87	101	50
4	2	108.25	108.70	0.45	325	2.22	4.60	215	66
5	2	108.80	109.05	0.25	155	1.75	3.13	89	57
6	3	157.85	158.10	0.25	375	1.42	2.96	194	52
7	4	33.60	33.95	0.35	420	1.61	2.40	191	45
8	4	102.80	103.05	0.25	376	1.56	2.86	196	52
9	6	31.95	32.20	0.25	314	1.76	3.83	161	51
10	7	86.75	87.10	0.35	503	1.57	3.58	241	48
11	7	101.95	102.25	0.30	227	1.78	3.86	111	49
12	7	142.75	143.00	0.25	229	1.54	2.37	124	54
13	8	11.00	11.25	0.25	217	1.72	4.01	113	52
14	11	63.75	64.05	0.30	233	1.68	3.75	120	52
15	12	1.20	1.45	0.25	531	1.56	3.39	275	52
16	16	17.20	17.60	0.40	529	1.76	3.86	269	51
17	20	20.00	20.30	0.30	642	1.44	3.27	316	49
18	20	35.60	35.95	0.35	351	1.81	3.96	213	61
19	21	10.25	10.65	0.40	263	1.93	4.98	146	56
20	22	40.05	40.30	0.25	239	1.48	2.61	127	53

TABLE 5 Candidate regions for population `CHB` using *iHS* on phased & unpolarized data

	CHR	START	END	LENGTH	#MRK	MEAN	MAX	#EXTR MRK	$\frac{\#\text{EXTR MRK}}{\#\text{MRK}}$ %
1	1	169.10	169.55	0.45	264	2.22	5.55	137	52
2	2	121.45	121.70	0.25	174	2.48	4.92	89	51
3	2	153.10	153.50	0.40	265	2.38	7.07	144	54
4	2	177.70	177.95	0.25	148	2.54	4.69	74	50
5	3	12.55	12.80	0.25	90	2.65	4.58	50	56
6	3	158.30	158.85	0.55	456	2.49	6.72	263	58
7	4	34.70	35.15	0.45	221	2.47	5.60	129	58
8	4	60.20	60.50	0.30	338	2.33	5.38	178	53
9	4	137.65	138.00	0.35	146	2.53	4.78	93	64
10	5	65.45	65.75	0.30	330	2.42	4.42	173	52
11	6	82.80	83.15	0.35	157	2.28	5.19	75	48
12	6	92.70	93.00	0.30	203	2.11	5.03	96	47
13	6	137.60	137.85	0.25	131	2.12	4.11	73	56
14	7	86.75	87.05	0.30	209	2.95	7.28	138	66
15	8	57.00	57.30	0.30	148	2.31	6.12	81	55
16	11	37.45	37.90	0.45	434	2.36	5.86	241	56
17	13	111.00	111.40	0.40	206	2.33	6.15	107	52
18	17	19.85	20.40	0.55	265	2.60	6.35	170	64
19	19	36.95	37.25	0.30	303	2.28	7.88	135	45
20	19	37.50	38.10	0.60	314	2.40	6.39	186	59

TABLE 6 Candidate regions for population `CHB` using *iHS* on unphased & polarized data

	CHR	START	END	LENGTH	#MRK	MEAN	MAX	#EXTR MRK	$\frac{\#EXTR\ MRK}{\#MRK}$ %
1	1	102.90	103.25	0.35	453	1.77	3.86	211	47
2	2	17.10	17.45	0.35	528	2.01	5.98	248	47
3	2	43.50	43.90	0.40	673	1.90	4.90	339	50
4	2	108.60	109.05	0.45	551	2.07	4.39	299	54
5	2	212.40	212.70	0.30	314	2.03	3.78	169	54
6	4	41.40	41.65	0.25	359	2.00	3.68	183	51
7	4	41.95	42.20	0.25	168	2.35	4.23	115	68
8	4	98.65	98.95	0.30	572	2.06	4.53	285	50
9	4	99.10	99.50	0.40	762	2.02	4.07	420	55
10	9	123.60	123.95	0.35	366	2.03	4.98	202	55
11	10	54.10	54.40	0.30	599	2.24	4.69	343	57
12	12	1.00	1.55	0.55	1144	2.18	4.79	655	57
13	12	55.05	55.35	0.30	350	1.94	3.74	179	51
14	14	49.50	49.75	0.25	218	2.08	3.78	119	55
15	15	30.70	31.00	0.30	247	2.30	4.49	164	66
16	16	17.20	17.55	0.35	464	2.12	4.93	266	57
17	16	65.50	65.85	0.35	266	2.00	3.83	158	59
18	20	35.60	36.10	0.50	688	1.98	4.44	384	56
19	21	10.25	10.65	0.40	262	2.86	6.58	195	74
20	22	39.25	39.55	0.30	297	1.99	3.92	145	49

TABLE 7 Candidate regions for population `JPT` using *iHS* on phased & polarized data

	CHR	START	END	LENGTH	#MRK	MEAN	MAX	#EXTR MRK	$\frac{\text{\#EXTR MRK}}{\text{\#MRK}}$ %
1	2	43.55	43.80	0.25	450	1.70	3.57	238	53
2	4	33.55	33.95	0.40	446	1.57	2.41	198	44
3	4	41.95	42.20	0.25	168	1.65	3.09	88	52
4	4	99.15	99.40	0.25	520	1.70	3.38	262	50
5	4	102.85	103.10	0.25	334	1.51	2.75	167	50
6	5	102.35	102.60	0.25	505	1.32	2.78	257	51
7	6	29.40	29.65	0.25	815	1.70	3.69	410	50
8	6	31.15	31.45	0.30	3922	1.66	5.85	1897	48
9	7	133.45	133.70	0.25	243	1.77	3.52	124	51
10	7	133.75	134.00	0.25	210	1.42	2.53	109	52
11	10	54.10	54.40	0.30	599	1.63	3.39	297	50
12	10	64.05	64.30	0.25	161	1.48	3.28	89	55
13	12	1.10	1.50	0.40	729	1.94	3.97	439	60
14	14	106.05	106.30	0.25	195	1.47	2.69	103	53
15	15	30.70	30.95	0.25	171	1.73	3.18	106	62
16	15	43.15	43.55	0.40	452	1.60	3.27	219	48
17	16	17.20	17.55	0.35	464	1.62	3.56	233	50
18	16	65.55	65.80	0.25	192	1.63	2.78	101	53
19	17	43.10	43.35	0.25	294	1.64	3.01	150	51
20	21	10.25	10.65	0.40	262	2.00	5.01	151	58

TABLE 8 Candidate regions for population `JPT` using *iHS* on phased & unpolarized data

	CHR	START	END	LENGTH	#MRK	MEAN	MAX	#EXTR MRK	$\frac{\text{\#EXTR MRK}}{\text{\#MRK}}$ %
1	2	39.20	39.45	0.25	116	2.20	4.46	62	53
2	3	125.50	125.75	0.25	83	2.45	5.20	45	54
3	3	147.40	147.65	0.25	111	2.57	5.50	73	66
4	4	99.15	99.45	0.30	157	2.59	6.53	94	60
5	4	156.70	156.95	0.25	161	2.27	5.34	87	54
6	6	54.85	55.15	0.30	157	2.42	6.07	92	59
7	7	56.40	56.65	0.25	78	2.77	5.09	52	67
8	7	113.25	113.50	0.25	97	2.56	8.00	52	54
9	9	30.15	30.40	0.25	149	2.47	5.77	75	50
10	9	103.95	104.20	0.25	87	2.66	6.05	46	53
11	10	63.70	64.15	0.45	320	2.58	6.57	197	62
12	11	37.65	37.90	0.25	103	2.42	4.60	63	61
13	12	1.05	1.45	0.40	130	2.51	5.51	76	58
14	12	73.25	73.65	0.40	318	2.42	5.99	167	53
15	12	98.90	99.20	0.30	142	1.93	6.75	60	42
16	15	46.90	47.20	0.30	249	2.25	6.72	124	50
17	15	67.65	67.90	0.25	158	2.23	4.52	84	53
18	18	31.20	31.45	0.25	109	2.55	5.41	60	55
19	19	22.55	22.90	0.35	493	2.39	7.25	263	53
20	19	37.50	38.05	0.55	291	2.44	5.03	174	60

TABLE 9 Candidate regions for population `JPT` using *iHS* on unphased & polarized data

	CHR	START	END	LENGTH	#MRK	MEAN	MAX	#EXTR MRK	$\frac{\text{\#EXTR MRK}}{\text{\#MRK}}$ %
1	1	160.95	161.20	0.25	377	2.11	4.60	208	55
2	2	98.20	98.65	0.45	786	2.05	4.05	446	57
3	2	181.20	181.50	0.30	716	1.88	4.85	333	47
4	2	193.90	194.15	0.25	390	1.85	4.46	197	51
5	4	13.20	13.60	0.40	947	1.90	4.71	427	45
6	4	106.75	107.00	0.25	238	2.10	5.18	128	54
7	7	65.60	65.95	0.35	336	1.92	4.61	163	49
8	10	100.30	100.70	0.40	883	2.03	5.11	432	49
9	10	102.80	103.20	0.40	690	2.02	4.08	379	55
10	12	79.00	79.70	0.70	1121	2.17	4.82	677	60
11	12	86.95	87.20	0.25	268	1.93	3.72	138	51
12	13	52.15	52.45	0.30	262	2.01	5.08	136	52
13	14	101.55	101.90	0.35	873	1.79	4.41	419	48
14	15	44.45	44.70	0.25	275	1.91	4.75	138	50
15	16	22.90	23.20	0.30	634	2.15	5.21	383	60
16	17	3.55	3.80	0.25	767	2.19	5.17	424	55
17	19	38.20	38.70	0.50	1062	2.02	5.26	553	52
18	20	38.70	38.95	0.25	563	2.07	4.47	314	56
19	21	9.75	10.10	0.35	208	2.84	5.05	169	81
20	21	10.25	10.65	0.40	262	3.39	5.27	240	92

TABLE 10 Candidate regions for population `YRI` using *iHS* on phased & polarized data

	CHR	START	END	LENGTH	#MRK	MEAN	MAX	#EXTR MRK	$\frac{\text{#EXTR MRK}}{\text{#MRK}}$ %
1	1	32.30	32.60	0.30	505	1.40	3.63	248	49
2	2	98.15	98.45	0.30	535	1.50	3.61	270	50
3	3	87.10	87.40	0.30	921	1.51	3.79	490	53
4	3	136.70	137.00	0.30	442	1.28	2.43	219	50
5	4	13.20	13.60	0.40	947	1.49	3.59	468	49
6	4	33.95	34.35	0.40	781	1.51	3.21	384	49
7	4	106.65	107.00	0.35	385	1.53	4.02	194	50
8	7	66.20	66.70	0.50	446	1.34	3.23	227	51
9	10	100.30	100.70	0.40	883	1.52	4.20	420	48
10	10	102.80	103.25	0.45	772	1.54	3.55	403	52
11	12	79.00	79.50	0.50	876	1.62	4.13	467	53
12	12	86.90	87.20	0.30	392	1.54	3.10	219	56
13	13	52.15	52.55	0.40	308	1.43	3.59	145	47
14	14	101.60	101.85	0.25	632	1.62	3.98	325	51
15	16	22.85	23.25	0.40	893	1.34	4.02	398	45
16	17	3.55	3.85	0.30	921	1.67	4.44	497	54
17	18	33.05	33.40	0.35	755	1.24	2.95	344	46
18	19	38.25	38.65	0.40	817	1.76	4.21	471	58
19	21	9.75	10.10	0.35	208	1.88	3.95	130	63
20	21	10.25	10.65	0.40	262	2.30	4.73	224	86

TABLE 11 Candidate regions for population `YRI` using *iHS* on phased & unpolarized data

	CHR	START	END	LENGTH	#MRK	MEAN	MAX	#EXTR MRK	$\frac{\#EXTR\ MRK}{\#MRK}\%$
1	2	82.90	83.25	0.35	450	2.01	4.09	224	50
2	2	98.25	98.50	0.25	92	1.91	3.72	48	52
3	2	192.90	193.15	0.25	172	2.16	5.19	92	53
4	4	132.20	132.45	0.25	278	2.02	3.88	149	54
5	5	15.00	15.35	0.35	340	2.05	4.46	182	54
6	6	130.20	130.45	0.25	305	2.02	3.94	158	52
7	7	54.30	54.55	0.25	341	1.97	4.38	178	52
8	7	69.65	69.95	0.30	123	2.11	3.99	72	59
9	10	37.85	38.10	0.25	117	2.10	3.56	61	52
10	10	38.15	38.50	0.35	208	1.94	3.73	105	50
11	10	100.35	100.60	0.25	80	2.44	4.23	58	73
12	11	9.90	10.30	0.40	468	1.93	4.95	236	50
13	11	38.40	38.65	0.25	190	2.01	5.83	100	53
14	12	79.10	79.55	0.45	155	2.31	4.04	105	68
15	12	82.10	82.50	0.40	181	2.06	4.30	95	52
16	12	82.55	82.85	0.30	180	2.12	4.48	94	52
17	16	48.15	48.45	0.30	241	2.02	3.52	126	52
18	18	54.15	54.45	0.30	147	1.88	3.87	74	50
19	19	38.50	38.75	0.25	174	2.00	3.90	90	52
20	20	38.70	39.00	0.30	169	2.19	4.92	97	57

TABLE 12 Candidate regions for population `YRI` using *iHS* on unphased & polarized data

	CHR	START	END	LENGTH	#MRK	MEAN	MAX	#EXTR MRK	$\frac{\#\text{EXTR MRK}}{\#\text{MRK}}\%$
1	1	1.20	1.60	0.40	558	2.08	4.38	304	54
2	2	82.85	83.10	0.25	548	2.14	5.86	282	51
3	2	121.30	121.80	0.50	569	2.05	4.26	339	60
4	2	134.35	136.20	1.85	2251	2.92	6.55	1796	80
5	4	30.10	30.40	0.30	514	1.98	4.04	258	50
6	4	33.50	34.55	1.05	1538	2.22	4.29	947	62
7	7	99.25	99.55	0.30	183	1.95	3.93	91	50
8	9	123.65	123.90	0.25	268	2.08	4.01	146	54
9	10	73.10	73.45	0.35	670	1.87	3.75	305	46
10	12	49.45	49.80	0.35	328	2.00	4.24	156	48
11	12	111.05	111.55	0.50	356	2.24	5.67	227	64
12	12	111.75	112.15	0.40	437	1.96	4.46	189	43
13	13	83.75	84.05	0.30	477	1.88	4.20	245	51
14	14	66.10	67.45	1.35	1754	2.11	4.46	1053	60
15	15	28.75	29.10	0.35	310	2.04	3.91	178	57
16	15	48.15	48.40	0.25	221	1.98	4.45	111	50
17	15	74.45	74.75	0.30	241	1.96	4.00	128	53
18	17	60.55	61.00	0.45	404	2.03	4.08	206	51
19	21	10.25	10.65	0.40	251	2.80	6.07	201	80
20	22	49.70	50.00	0.30	795	2.03	5.33	388	49

TABLE 13 Candidate regions for combined population `CEU+GBR` using *iHS* on phased & polarized data

	CHR	START	END	LENGTH	#MRK	MEAN	MAX	#EXTR MRK	$\frac{\#\text{EXTR MRK}}{\#\text{MRK}}$ %
1	1	1.25	1.60	0.35	398	1.57	3.82	196	49
2	1	185.50	185.80	0.30	467	1.50	3.04	236	51
3	2	21.35	21.60	0.25	164	1.54	2.73	98	60
4	2	134.45	136.20	1.75	2081	2.41	5.02	1595	77
5	2	188.95	189.30	0.35	526	1.43	2.90	257	49
6	3	95.35	95.65	0.30	677	1.54	2.77	359	53
7	3	103.40	103.75	0.35	1164	1.37	2.90	534	46
8	4	0.45	0.75	0.30	528	1.38	2.90	241	46
9	4	33.50	34.15	0.65	658	1.65	2.78	354	54
10	4	34.20	34.45	0.25	498	1.74	3.37	255	51
11	4	102.70	103.10	0.40	551	1.52	2.74	291	53
12	5	116.10	116.40	0.30	939	1.46	3.52	469	50
13	12	111.10	111.55	0.45	350	1.76	3.84	175	50
14	12	111.80	112.15	0.35	405	1.58	3.96	179	44
15	14	66.35	66.75	0.40	743	1.59	2.90	403	54
16	14	67.20	67.45	0.25	186	1.68	3.15	95	51
17	15	28.75	29.05	0.30	268	1.58	2.55	145	54
18	17	43.05	43.30	0.25	295	1.51	2.96	158	54
19	17	60.55	60.90	0.35	338	1.68	2.68	175	52
20	21	10.25	10.65	0.40	251	1.90	4.59	153	61

TABLE 14 Candidate regions for combined population `CEU+GBR` using *iHS* on phased & unpolarized data

	CHR	START	END	LENGTH	#MRK	MEAN	MAX	#EXTR MRK	$\frac{\#\text{EXTR MRK}}{\#\text{MRK}}$ %
1	1	64.65	64.90	0.25	118	2.28	4.96	63	53
2	2	13.50	13.85	0.35	330	2.34	6.54	174	53
3	2	134.60	135.20	0.60	423	2.45	5.15	264	62
4	2	135.50	136.10	0.60	302	2.74	5.31	229	76
5	2	174.00	174.35	0.35	380	1.72	5.12	154	41
6	4	41.85	42.20	0.35	322	2.34	5.36	178	55
7	4	80.55	80.80	0.25	75	2.49	4.45	42	56
8	4	140.25	140.55	0.30	115	2.24	6.06	65	57
9	5	21.75	22.05	0.30	170	2.31	5.26	87	51
10	5	110.60	111.05	0.45	162	2.87	6.35	127	78
11	6	34.55	35.00	0.45	362	2.24	4.65	200	55
12	6	120.90	121.25	0.35	239	2.39	4.16	142	59
13	6	144.65	145.00	0.35	284	2.21	4.99	138	49
14	8	51.75	52.05	0.30	310	2.21	6.27	156	50
15	9	72.60	72.85	0.25	145	2.25	4.86	78	54
16	10	44.05	44.30	0.25	151	1.99	5.16	77	51
17	12	71.55	71.85	0.30	103	2.12	5.26	50	49
18	13	83.75	84.00	0.25	110	2.22	4.17	57	52
19	17	65.15	65.45	0.30	202	2.15	4.14	106	52
20	22	49.70	49.95	0.25	263	2.23	4.74	144	55

TABLE 15 Candidate regions for combined population `CEU+GBR` using *iHS* on unphased & polarized data

	CHR	START	END	LENGTH	#MRK	MEAN	MAX	#EXTR MRK	$\frac{\text{\#EXTR MRK}}{\text{\#MRK}}$ %
1	2	17.10	17.50	0.40	551	2.05	6.12	261	47
2	2	21.60	22.00	0.40	211	1.97	3.73	109	52
3	2	103.20	103.45	0.25	212	1.86	3.54	106	50
4	2	108.25	109.10	0.85	796	2.11	5.06	470	59
5	2	196.30	196.60	0.30	212	1.95	4.41	121	57
6	2	212.40	212.75	0.35	389	1.90	4.44	193	50
7	2	218.65	218.95	0.30	338	1.88	3.92	163	48
8	3	104.85	105.15	0.30	553	1.85	4.53	255	46
9	3	139.30	139.55	0.25	221	1.90	4.24	117	53
10	7	86.75	87.00	0.25	351	2.10	4.59	176	50
11	7	101.95	102.25	0.30	221	2.09	4.64	112	51
12	8	10.90	11.25	0.35	311	2.10	4.67	166	53
13	9	123.55	123.85	0.30	359	1.94	4.53	174	48
14	11	63.75	64.10	0.35	284	1.72	3.91	136	48
15	12	1.15	1.40	0.25	580	2.08	4.29	303	52
16	16	17.15	17.60	0.45	591	2.10	5.16	314	53
17	17	62.85	63.30	0.45	316	1.93	4.19	154	49
18	20	35.60	36.10	0.50	601	2.12	5.04	347	58
19	21	10.25	10.65	0.40	266	2.87	5.84	200	75
20	22	32.00	32.25	0.25	263	1.83	3.97	138	52

TABLE 16 Candidate regions for combined population `CHB+CHS` using *iHS* on phased & polarized data

	CHR	START	END	LENGTH	#MRK	MEAN	MAX	#EXTR MRK	$\frac{\#\text{EXTR MRK}}{\#\text{MRK}}$ %
1	1	113.40	113.65	0.25	291	1.50	3.01	146	50
2	2	17.05	17.50	0.45	624	1.51	4.82	283	45
3	2	108.30	109.00	0.70	549	1.80	3.51	336	61
4	2	212.45	212.70	0.25	272	1.67	2.94	152	56
5	2	218.70	218.95	0.25	280	1.61	2.73	152	54
6	3	157.90	158.15	0.25	354	1.42	2.91	183	52
7	4	33.60	33.95	0.35	413	1.57	2.32	185	45
8	4	41.40	41.65	0.25	382	1.59	3.05	206	54
9	7	86.75	87.05	0.30	446	1.64	3.56	242	54
10	7	101.95	102.20	0.25	171	1.86	3.71	90	53
11	7	142.75	143.00	0.25	222	1.67	2.71	132	59
12	8	10.95	11.30	0.35	376	1.57	3.27	175	47
13	11	63.75	64.05	0.30	223	1.68	3.77	120	54
14	12	1.15	1.40	0.25	580	1.58	3.69	291	50
15	16	17.15	17.60	0.45	591	1.73	3.71	321	54
16	16	75.65	75.90	0.25	162	1.63	2.90	83	51
17	17	43.10	43.35	0.25	293	1.65	3.19	161	55
18	20	20.00	20.30	0.30	629	1.49	3.12	317	50
19	20	35.65	35.95	0.30	408	1.67	4.03	212	52
20	21	10.25	10.65	0.40	266	2.02	5.00	160	60

TABLE 17 Candidate regions for combined population `CHB+CHS` using *iHS* on phased & unpolarized data

	CHR	START	END	LENGTH	#MRK	MEAN	MAX	#EXTR MRK	$\frac{\text{\#EXTR MRK}}{\text{\#MRK}}$ %
1	2	81.40	81.65	0.25	183	2.70	6.91	116	63
2	2	108.00	108.35	0.35	185	2.36	6.93	87	47
3	2	218.40	218.90	0.50	347	2.51	8.03	179	52
4	3	158.35	158.85	0.50	567	2.32	7.18	308	54
5	5	43.30	43.70	0.40	134	2.19	6.32	68	51
6	5	65.45	65.75	0.30	354	2.40	5.20	184	52
7	6	82.90	83.20	0.30	246	2.44	5.67	127	52
8	7	5.55	5.80	0.25	98	2.61	4.45	68	69
9	10	38.15	38.50	0.35	214	2.23	4.44	113	53
10	10	92.45	92.80	0.35	140	2.33	4.59	79	56
11	11	71.65	71.90	0.25	112	2.37	4.80	57	51
12	12	1.00	1.35	0.35	228	2.22	5.82	111	49
13	12	32.95	33.45	0.50	673	2.34	6.42	349	52
14	12	44.05	44.35	0.30	81	2.57	6.11	56	69
15	12	88.50	88.75	0.25	102	2.32	5.09	55	54
16	15	22.25	22.50	0.25	93	2.17	3.98	47	51
17	15	30.85	31.10	0.25	83	2.47	6.60	48	58
18	15	63.55	63.90	0.35	104	2.85	4.92	84	81
19	17	19.85	20.40	0.55	291	2.79	6.95	201	69
20	19	31.45	31.70	0.25	107	2.32	6.56	55	51

TABLE 18 Candidate regions for combined population `CHB+CHS` using *iHS* on unphased & polarized data

	CHR	START	END	LENGTH	#MRK	MEAN	MAX	#EXTR MRK	$\frac{\#\text{EXTR MRK}}{\#\text{MRK}}$ %
1	2	17.25	17.70	0.45	942	2.72	3.98	507	54
2	2	108.45	108.70	0.25	633	2.79	4.53	371	59
3	2	134.45	136.20	1.75	2902	3.65	9.77	2356	81
4	2	136.35	136.65	0.30	863	2.48	4.35	394	46
5	2	195.30	195.55	0.25	528	2.73	4.44	302	57
6	3	107.40	107.85	0.45	460	2.94	5.00	288	63
7	4	99.05	99.55	0.50	1097	2.46	4.18	579	53
8	5	117.95	118.50	0.55	1227	2.58	4.06	826	67
9	6	68.20	68.50	0.30	1117	2.54	3.99	689	62
10	8	30.05	30.40	0.35	773	2.10	4.17	301	39
11	11	61.05	61.35	0.30	368	2.09	3.56	174	47
12	12	99.75	100.10	0.35	544	2.22	4.16	232	43
13	14	68.95	69.25	0.30	461	2.58	3.85	257	56
14	15	28.15	28.40	0.25	184	2.84	3.87	118	64
15	15	28.75	29.15	0.40	643	2.31	4.34	364	57
16	15	36.10	36.35	0.25	655	2.34	4.02	354	54
17	15	47.85	48.40	0.55	740	3.18	6.79	404	55
18	15	74.60	74.90	0.30	382	2.54	3.93	177	46
19	16	65.55	65.85	0.30	510	2.44	4.25	258	51
20	18	7.45	7.75	0.30	679	2.30	3.98	314	46

TABLE 19 Candidate regions for population `CEU` vs `CHB` using *XP-EHH* on phased data

	CHR	START	END	LENGTH	#MRK	MEAN	MAX	#EXTR MRK	$\frac{\#EXTR\ MRK}{\#MRK}$ %
1	1	113.40	113.80	0.40	555	2.06	3.86	247	45
2	2	17.35	17.85	0.50	1108	2.38	4.73	640	58
3	2	106.40	106.65	0.25	371	2.17	4.56	187	50
4	2	108.45	108.85	0.40	832	2.26	4.94	415	50
5	2	134.30	136.30	2.00	3417	3.43	9.32	2828	83
6	2	136.35	136.65	0.30	863	2.23	4.69	413	48
7	2	195.30	195.65	0.35	751	2.29	4.69	367	49
8	3	107.40	107.85	0.45	460	2.36	5.01	248	54
9	3	129.90	130.15	0.25	287	2.09	3.92	151	53
10	4	99.10	99.55	0.45	1057	2.43	5.33	587	56
11	5	103.90	104.30	0.40	719	1.97	5.21	317	44
12	5	118.20	118.50	0.30	672	2.25	4.23	361	54
13	6	68.20	68.65	0.45	1468	2.22	4.80	752	51
14	6	92.60	92.95	0.35	1253	1.92	5.10	512	41
15	12	41.70	42.05	0.35	833	1.76	4.25	347	42
16	13	21.20	21.50	0.30	494	2.04	4.01	231	47
17	13	62.75	63.05	0.30	870	2.16	4.00	411	47
18	15	47.85	48.45	0.60	833	2.70	6.97	465	56
19	15	74.60	74.95	0.35	448	2.03	4.27	201	45
20	18	7.45	7.75	0.30	679	1.97	3.92	306	45

TABLE 20 Candidate regions for population `CEU` vs `CHB` using *Rsb* on phased data

	CHR	START	END	LENGTH	#MRK	MEAN	MAX	#EXTR MRK	$\frac{\#EXTR\ MRK}{\#MRK}$ %
1	2	17.35	17.75	0.40	763	2.22	4.73	389	51
2	2	108.40	108.80	0.40	920	2.43	5.98	514	56
3	2	134.35	136.25	1.90	3206	2.87	9.15	2168	68
4	2	136.35	136.60	0.25	779	2.18	4.97	394	51
5	3	107.50	107.85	0.35	281	2.05	4.36	124	44
6	3	197.00	197.35	0.35	678	2.03	5.18	316	47
7	4	99.15	99.45	0.30	787	2.31	5.18	396	50
8	6	68.20	68.70	0.50	1530	2.21	5.55	804	53
9	6	92.65	93.00	0.35	1310	2.00	4.84	601	46
10	7	86.20	86.55	0.35	795	2.35	5.11	430	54
11	9	91.25	91.50	0.25	675	2.29	5.34	340	50
12	12	19.10	19.40	0.30	714	2.24	5.17	374	52
13	12	41.75	42.00	0.25	609	2.16	4.36	308	51
14	13	62.75	63.10	0.35	954	2.14	4.41	437	46
15	14	68.95	69.20	0.25	400	2.29	5.79	208	52
16	14	105.80	106.25	0.45	763	2.05	5.47	353	46
17	15	28.15	28.40	0.25	184	2.30	5.51	96	52
18	15	36.05	36.40	0.35	946	2.16	5.57	486	51
19	15	47.90	48.40	0.50	597	2.46	6.81	322	54
20	22	43.55	43.80	0.25	600	2.28	5.26	305	51

TABLE 21 Candidate regions for population `CEU` vs `CHB` using *XP-EHH*/*Rsb* on unphased data

	CHR	START	END	LENGTH	#MRK	MEAN	MAX	#EXTR MRK	$\frac{\#EXTR\ MRK}{\#MRK}$ %
1	1	35.05	35.35	0.30	447	2.58	3.84	209	47
2	1	198.25	198.55	0.30	681	2.64	3.63	386	57
3	2	17.15	17.65	0.50	1031	2.60	3.91	569	55
4	2	134.35	136.15	1.80	3066	3.60	9.53	2543	83
5	2	176.60	176.90	0.30	426	2.53	3.79	220	52
6	3	107.50	107.75	0.25	293	2.93	4.49	153	52
7	4	99.05	99.55	0.50	1104	2.80	4.50	725	66
8	5	117.95	118.40	0.45	1055	2.49	3.86	612	58
9	5	142.65	143.05	0.40	914	2.51	5.32	395	43
10	6	68.20	68.50	0.30	1086	2.53	3.92	655	60
11	6	69.00	69.35	0.35	481	2.66	4.40	215	45
12	7	112.40	112.80	0.40	697	2.49	4.28	326	47
13	8	30.05	30.30	0.25	510	2.49	4.58	263	52
14	10	54.10	54.45	0.35	1368	2.73	4.52	767	56
15	12	99.75	100.10	0.35	432	2.17	3.97	179	41
16	14	68.95	69.20	0.25	315	2.62	3.57	183	58
17	15	28.15	28.40	0.25	182	2.83	4.21	122	67
18	15	36.05	36.35	0.30	706	2.41	4.26	381	54
19	15	47.85	48.70	0.85	1128	2.93	6.21	646	57
20	18	7.45	7.75	0.30	639	2.25	3.90	306	48

TABLE 22 Candidate regions for population `CEU` vs `JPT` using *XP-EHH* on phased data

	CHR	START	END	LENGTH	#MRK	MEAN	MAX	#EXTR MRK	$\frac{\#EXTR\ MRK}{\#MRK}$ %
1	2	17.25	17.80	0.55	1078	2.21	4.47	551	51
2	2	134.30	136.60	2.30	4428	3.19	8.81	3419	77
3	2	176.60	176.90	0.30	426	2.02	3.90	204	48
4	3	107.45	107.75	0.30	378	2.27	5.02	189	50
5	4	99.05	99.60	0.55	1225	2.46	4.84	747	61
6	5	103.90	104.35	0.45	739	1.98	5.08	322	44
7	5	142.65	143.10	0.45	993	2.17	5.18	488	49
8	6	68.20	68.65	0.45	1422	2.24	4.94	744	52
9	6	92.60	92.95	0.35	1174	1.86	4.11	513	44
10	7	112.40	112.75	0.35	643	2.03	4.50	290	45
11	8	103.60	103.90	0.30	487	2.16	3.88	244	50
12	9	91.30	91.55	0.25	739	2.30	4.65	392	53
13	10	54.15	54.40	0.25	971	2.40	4.93	519	53
14	11	71.20	71.55	0.35	995	2.06	5.27	433	44
15	11	101.05	101.30	0.25	343	2.08	4.06	177	52
16	12	41.70	42.05	0.35	811	1.81	4.02	349	43
17	13	21.15	21.55	0.40	603	1.83	3.72	249	41
18	13	43.65	44.05	0.40	984	2.04	5.40	415	42
19	15	36.00	36.35	0.35	860	1.98	4.22	413	48
20	15	47.85	48.70	0.85	1128	2.61	6.65	672	60

TABLE 23 Candidate regions for population `CEU` vs `JPT` using *Rsb* on phased data

	CHR	START	END	LENGTH	#MRK	MEAN	MAX	#EXTR MRK	$\frac{\text{\#EXTR MRK}}{\text{\#MRK}}$ %
1	1	198.25	198.50	0.25	606	2.23	4.97	304	50
2	2	17.35	17.75	0.40	655	2.23	4.38	339	52
3	2	134.30	136.25	1.95	3382	2.76	11.40	2294	68
4	4	99.05	99.45	0.40	895	2.36	5.21	479	54
5	5	142.70	143.10	0.40	885	2.31	6.21	454	51
6	6	68.25	68.70	0.45	1360	2.12	5.46	669	49
7	6	92.60	92.85	0.25	709	2.16	4.28	356	50
8	7	86.20	86.45	0.25	536	2.42	4.84	294	55
9	7	112.45	112.70	0.25	509	2.27	4.55	255	50
10	8	32.35	32.60	0.25	719	2.28	5.87	380	53
11	8	103.55	103.80	0.25	400	2.20	4.85	202	51
12	9	91.30	91.55	0.25	739	2.30	5.43	370	50
13	11	71.20	71.55	0.35	995	2.08	6.91	430	43
14	12	41.70	42.05	0.35	811	2.14	6.05	388	48
15	13	43.65	44.05	0.40	984	1.83	5.59	373	38
16	14	68.95	69.20	0.25	315	2.46	5.55	180	57
17	15	36.00	36.40	0.40	975	2.10	5.55	468	48
18	15	47.85	48.50	0.65	893	2.43	6.42	484	54
19	15	74.65	74.95	0.30	396	2.18	4.25	203	51
20	22	15.95	16.20	0.25	322	2.36	4.25	165	51

TABLE 24 Candidate regions for population `CEU` vs `JPT` using *XP-EHH*/*Rsb* on unphased data

	CHR	START	END	LENGTH	#MRK	MEAN	MAX	#EXTR MRK	$\frac{\#EXTR\ MRK}{\#MRK}$ %
1	1	34.90	35.20	0.30	425	2.50	4.24	223	52
2	1	40.95	41.35	0.40	674	2.46	4.70	327	49
3	1	113.60	113.85	0.25	283	2.54	3.58	143	51
4	1	160.90	161.20	0.30	569	2.56	4.31	323	57
5	1	206.10	206.35	0.25	224	2.73	4.86	128	57
6	2	134.55	136.25	1.70	2797	3.41	6.57	2246	80
7	3	110.75	111.10	0.35	551	2.34	3.93	288	52
8	5	110.20	110.60	0.40	1034	2.49	4.73	511	49
9	6	33.10	33.35	0.25	1190	2.18	3.89	719	60
10	6	94.65	95.05	0.40	583	2.57	4.79	306	52
11	9	97.75	98.05	0.30	548	2.25	3.55	253	46
12	10	64.00	64.30	0.30	524	2.36	4.12	264	50
13	10	101.35	101.60	0.25	442	2.47	3.89	235	53
14	11	78.40	78.65	0.25	558	2.58	4.11	285	51
15	13	56.85	57.30	0.45	839	2.25	4.93	398	47
16	15	28.10	28.40	0.30	328	2.70	4.37	173	53
17	15	48.00	48.40	0.40	439	2.87	5.80	252	57
18	16	22.85	23.15	0.30	850	2.07	4.03	405	48
19	17	3.55	3.80	0.25	757	2.70	4.70	389	51
20	17	60.40	60.65	0.25	182	2.59	4.91	91	50

TABLE 25 Candidate regions for population `CEU` vs `YRI` using *XP-EHH* on phased data

	CHR	START	END	LENGTH	#MRK	MEAN	MAX	#EXTR MRK	$\frac{\text{\#EXTR MRK}}{\text{\#MRK}}$ %
1	1	34.90	35.20	0.30	425	2.08	4.00	219	52
2	1	40.95	41.30	0.35	525	2.22	4.38	292	56
3	1	160.90	161.20	0.30	569	2.07	4.19	286	50
4	1	206.05	206.35	0.30	280	2.01	4.88	145	52
5	2	134.45	136.25	1.80	3163	3.13	6.87	2536	80
6	2	136.35	136.60	0.25	809	2.22	4.48	425	53
7	2	237.35	237.60	0.25	849	2.10	5.50	452	53
8	3	110.80	111.10	0.30	440	2.26	3.98	250	57
9	4	80.35	80.65	0.30	685	1.97	4.15	334	49
10	5	75.70	76.00	0.30	1010	1.98	4.87	498	49
11	5	110.20	110.60	0.40	1034	2.21	4.60	562	54
12	6	94.70	95.05	0.35	418	2.28	4.41	242	58
13	10	37.65	37.90	0.25	459	2.11	3.76	251	55
14	10	64.05	64.30	0.25	389	2.15	4.41	207	53
15	12	111.10	111.55	0.45	612	2.23	4.08	335	55
16	14	48.45	48.80	0.35	990	2.09	4.66	460	46
17	15	48.00	48.40	0.40	439	2.63	5.25	296	67
18	17	3.50	3.85	0.35	1132	1.97	5.64	518	46
19	21	9.75	10.10	0.35	237	2.27	4.47	145	61
20	22	49.70	50.00	0.30	857	1.87	4.80	370	43

TABLE 26 Candidate regions for population `CEU` vs `YRI` using *Rsb* on phased data

	CHR	START	END	LENGTH	#MRK	MEAN	MAX	#EXTR MRK	$\frac{\text{\#EXTR MRK}}{\text{\#MRK}}$ %
1	1	40.95	41.30	0.35	525	2.11	4.29	286	54
2	2	134.50	136.20	1.70	2866	2.42	5.84	1891	66
3	2	237.30	237.60	0.30	1053	1.80	6.08	498	47
4	3	110.80	111.05	0.25	389	2.13	4.24	215	55
5	3	163.90	164.40	0.50	1773	2.19	4.34	1067	60
6	4	19.30	19.75	0.45	1508	2.16	4.93	861	57
7	4	80.40	80.70	0.30	716	1.90	3.88	342	48
8	6	68.40	68.65	0.25	792	1.91	4.43	426	54
9	6	71.95	72.30	0.35	736	1.88	4.54	336	46
10	6	94.65	95.05	0.40	583	2.07	4.80	294	50
11	8	83.05	83.40	0.35	1178	1.92	4.17	553	47
12	10	9.55	9.90	0.35	684	1.90	4.65	329	48
13	10	37.65	37.90	0.25	459	2.02	3.44	255	56
14	11	89.00	89.40	0.40	950	1.93	4.61	431	45
15	12	111.30	111.55	0.25	262	2.05	3.86	149	57
16	13	57.05	57.30	0.25	470	2.00	4.79	237	50
17	14	48.40	48.80	0.40	1107	1.93	4.57	532	48
18	15	48.00	48.40	0.40	439	2.03	5.09	247	56
19	16	14.50	14.75	0.25	176	2.08	4.24	104	59
20	19	37.70	38.00	0.30	560	1.96	4.26	279	50

TABLE 27 Candidate regions for population `CEU` vs `YRI` using *XP-EHH*/*Rsb* on unphased data

	CHR	START	END	LENGTH	#MRK	MEAN	MAX	#EXTR MRK	$\frac{\#EXTR\ MRK}{\#MRK}$ %
1	1	198.00	198.45	0.45	1267	2.37	4.89	656	52
2	2	86.30	86.60	0.30	767	2.45	3.32	371	48
3	3	84.10	84.35	0.25	568	2.47	3.87	301	53
4	3	147.40	147.70	0.30	649	2.38	5.80	282	43
5	4	34.80	35.05	0.25	918	2.47	4.21	529	58
6	4	97.80	98.10	0.30	1018	2.79	5.28	576	57
7	4	98.65	99.10	0.45	935	2.72	5.66	491	53
8	4	126.70	127.00	0.30	935	2.50	4.08	467	50
9	4	177.00	177.25	0.25	833	2.15	3.77	417	50
10	6	31.85	32.10	0.25	502	3.12	6.22	338	67
11	7	63.00	63.50	0.50	817	2.65	5.81	450	55
12	8	31.65	31.95	0.30	881	2.25	3.82	385	44
13	8	93.40	93.85	0.45	757	2.50	6.03	336	44
14	10	45.70	46.15	0.45	431	2.52	5.32	210	49
15	10	92.70	93.15	0.45	885	2.63	4.77	595	67
16	12	56.75	57.30	0.55	1047	2.65	5.30	565	54
17	12	70.15	70.45	0.30	877	2.32	3.76	432	49
18	13	99.45	99.70	0.25	745	2.69	4.72	408	55
19	14	43.20	43.50	0.30	1002	2.18	4.75	444	44
20	20	48.90	49.35	0.45	1353	2.55	4.43	716	53

TABLE 28 Candidate regions for population `CHB` vs `JPT` using *XP-EHH* on phased data

	CHR	START	END	LENGTH	#MRK	MEAN	MAX	#EXTR MRK	$\frac{\text{\#EXTR MRK}}{\text{\#MRK}}$ %
1	1	198.00	198.45	0.45	1267	2.20	5.09	652	51
2	2	135.70	136.00	0.30	799	2.07	5.01	389	49
3	3	69.35	69.60	0.25	1073	2.52	7.66	558	52
4	3	147.40	147.70	0.30	649	2.24	6.82	286	44
5	4	97.25	97.55	0.30	661	2.32	5.32	322	49
6	4	97.65	98.20	0.55	1805	2.29	6.36	864	48
7	4	98.60	99.00	0.40	985	2.62	5.68	519	53
8	4	120.65	121.05	0.40	1795	2.25	4.22	918	51
9	6	31.15	31.40	0.25	4738	2.41	9.07	2369	50
10	6	31.75	32.15	0.40	829	2.63	7.38	452	55
11	7	55.60	55.90	0.30	525	2.28	4.31	290	55
12	7	63.00	63.50	0.50	817	2.43	6.33	476	58
13	8	93.40	93.85	0.45	757	2.50	6.30	376	50
14	10	45.75	46.20	0.45	329	2.65	5.34	215	65
15	10	92.80	93.10	0.30	539	2.69	5.21	327	61
16	12	9.40	9.65	0.25	175	2.25	4.08	99	57
17	12	56.80	57.30	0.50	968	2.52	6.00	524	54
18	13	99.45	99.70	0.25	745	2.33	5.36	400	54
19	14	43.15	43.50	0.35	1178	1.92	5.15	478	41
20	20	48.90	49.35	0.45	1353	2.32	5.17	736	54

TABLE 29 Candidate regions for population `CHB` vs `JPT` using *Rsb* on phased data

	CHR	START	END	LENGTH	#MRK	MEAN	MAX	#EXTR MRK	$\frac{\#\text{EXTR MRK}}{\#\text{MRK}}$ %
1	1	86.25	86.60	0.35	1097	1.97	5.87	475	43
2	1	198.10	198.35	0.25	676	2.37	4.85	343	51
3	4	98.70	99.00	0.30	717	2.67	6.70	351	49
4	4	126.60	127.00	0.40	1184	2.20	4.97	576	49
5	4	146.75	147.00	0.25	647	2.30	4.64	329	51
6	5	63.25	63.50	0.25	702	2.34	5.29	371	53
7	5	142.80	143.10	0.30	933	2.22	6.88	452	48
8	6	31.65	32.00	0.35	815	2.14	4.71	365	45
9	7	149.40	149.70	0.30	875	2.13	5.84	412	47
10	8	58.10	58.55	0.45	1624	2.17	6.13	779	48
11	10	92.00	92.30	0.30	421	2.40	5.53	203	48
12	11	56.75	57.10	0.35	749	2.56	5.64	457	61
13	12	37.95	38.20	0.25	293	2.32	4.23	152	52
14	12	38.30	38.65	0.35	854	2.23	5.01	395	46
15	12	46.70	46.95	0.25	774	2.51	5.45	449	58
16	12	104.70	105.05	0.35	1163	2.44	6.50	624	54
17	13	82.75	83.15	0.40	1412	2.28	5.91	742	53
18	14	34.90	35.15	0.25	768	2.42	6.83	428	56
19	18	42.90	43.15	0.25	506	2.26	4.96	268	53
20	21	14.10	14.55	0.45	2036	2.26	8.40	996	49

TABLE 30 Candidate regions for population `CHB` vs `JPT` using *XP-EHH*/*Rsb* on unphased data

	CHR	START	END	LENGTH	#MRK	MEAN	MAX	#EXTR MRK	$\frac{\#EXTR\ MRK}{\#MRK}$ %
1	1	41.05	41.35	0.30	462	2.55	4.19	253	55
2	1	160.90	161.20	0.30	555	2.36	3.56	265	48
3	2	108.25	108.55	0.30	685	2.37	4.43	337	49
4	2	108.60	109.05	0.45	851	2.65	4.86	467	55
5	2	200.05	200.45	0.40	541	2.37	4.90	259	48
6	3	26.05	26.40	0.35	425	2.29	4.20	191	45
7	4	105.80	106.05	0.25	310	2.02	3.40	155	50
8	5	118.15	118.40	0.25	679	2.50	3.74	341	50
9	6	33.10	33.35	0.25	1161	2.71	4.62	752	65
10	6	128.80	129.05	0.25	381	2.62	4.04	214	56
11	7	3.70	4.00	0.30	477	2.35	4.07	229	48
12	7	66.40	67.00	0.60	624	2.54	4.32	325	52
13	8	11.00	11.25	0.25	765	2.49	4.70	386	50
14	10	54.10	54.55	0.45	1644	2.37	4.50	791	48
15	10	102.75	103.25	0.50	988	2.51	6.02	608	62
16	12	126.35	126.60	0.25	730	2.48	5.03	369	51
17	13	56.85	57.65	0.80	1387	2.63	3.81	937	68
18	16	65.55	65.80	0.25	428	2.66	4.38	226	53
19	19	38.15	38.50	0.35	842	1.96	3.98	411	49
20	22	46.20	46.45	0.25	281	2.93	5.59	166	59

TABLE 31 Candidate regions for population `CHB` vs `YRI` using *XP-EHH* on phased data

	CHR	START	END	LENGTH	#MRK	MEAN	MAX	#EXTR MRK	$\frac{\#\text{EXTR MRK}}{\#\text{MRK}}$ %
1	2	108.60	109.10	0.50	959	2.19	4.52	518	54
2	2	199.75	200.00	0.25	275	2.15	3.88	140	51
3	2	200.05	200.45	0.40	541	2.12	4.52	298	55
4	2	218.50	218.75	0.25	357	2.21	3.96	182	51
5	3	26.00	26.40	0.40	502	1.87	3.95	216	43
6	3	87.10	87.45	0.35	1163	2.04	4.41	645	55
7	4	46.90	47.15	0.25	507	2.16	3.64	263	52
8	6	31.30	31.55	0.25	3133	2.19	5.65	1575	50
9	6	32.40	32.85	0.45	11 838	2.29	5.95	6736	57
10	6	33.10	33.35	0.25	1161	2.47	6.02	734	63
11	10	54.15	54.40	0.25	993	2.25	4.60	523	53
12	10	102.80	103.30	0.50	968	2.31	5.68	598	62
13	10	104.75	105.10	0.35	704	2.02	4.28	335	48
14	12	126.30	126.65	0.35	1030	1.93	4.85	471	46
15	13	57.05	57.50	0.45	894	2.40	4.76	507	57
16	16	65.55	65.85	0.30	524	2.19	4.44	262	50
17	17	64.85	65.10	0.25	236	2.29	4.51	132	56
18	19	38.15	38.50	0.35	842	1.81	5.04	381	45
19	21	9.80	10.10	0.30	230	2.24	4.01	117	51
20	22	46.15	46.45	0.30	353	2.12	5.08	178	50

TABLE 32 Candidate regions for population `CHB` vs `YRI` using *Rsb* on phased data

	CHR	START	END	LENGTH	#MRK	MEAN	MAX	#EXTR MRK	$\frac{\#EXTR\ MRK}{\#MRK}$ %
1	2	27.05	27.35	0.30	258	1.99	3.40	130	50
2	2	108.60	109.05	0.45	851	2.04	4.60	441	52
3	2	199.75	200.45	0.70	877	2.01	5.38	453	52
4	3	26.10	26.35	0.25	270	1.91	3.59	138	51
5	4	39.45	39.70	0.25	608	1.99	3.98	310	51
6	4	46.75	47.15	0.40	722	2.03	3.69	354	49
7	4	99.05	99.30	0.25	307	1.94	3.73	155	50
8	6	31.15	31.55	0.40	5785	2.09	7.93	2861	49
9	6	62.65	62.95	0.30	969	2.12	4.46	513	53
10	6	65.50	65.95	0.45	1633	2.01	4.93	856	52
11	6	74.45	74.80	0.35	948	1.96	4.33	480	51
12	7	66.70	66.95	0.25	283	2.04	3.74	144	51
13	8	84.90	85.20	0.30	506	1.78	4.15	234	46
14	9	123.65	124.00	0.35	672	1.98	4.55	337	50
15	10	102.75	103.30	0.55	1086	2.06	5.84	622	57
16	10	104.80	105.05	0.25	492	2.20	4.10	287	58
17	10	105.50	105.75	0.25	343	2.18	4.15	192	56
18	13	57.05	57.50	0.45	894	2.22	5.11	513	57
19	13	82.85	83.10	0.25	748	2.12	4.46	374	50
20	19	38.15	38.45	0.30	704	1.81	5.41	344	49

TABLE 33 Candidate regions for population `CHB` vs `YRI` using *XP-EHH*/*Rsb* on unphased data

	CHR	START	END	LENGTH	#MRK	MEAN	MAX	#EXTR MRK	$\frac{\#EXTR\ MRK}{\#MRK}$ %
1	1	41.00	41.35	0.35	499	2.73	4.34	299	60
2	1	50.15	50.45	0.30	360	2.37	3.97	174	48
3	1	103.00	103.35	0.35	942	2.21	4.40	415	44
4	1	160.90	161.20	0.30	536	2.33	3.41	288	54
5	2	108.70	108.95	0.25	391	2.55	4.20	204	52
6	2	176.60	176.85	0.25	367	2.61	4.42	192	52
7	2	200.05	200.45	0.40	539	2.53	4.68	329	61
8	3	175.50	175.75	0.25	667	2.61	4.88	372	56
9	6	33.10	33.35	0.25	1088	2.20	3.78	600	55
10	7	3.70	4.05	0.35	656	2.42	4.03	332	51
11	7	66.05	66.35	0.30	502	2.53	3.49	237	47
12	7	66.40	67.05	0.65	730	2.91	5.17	487	67
13	10	54.10	54.55	0.45	1615	2.69	4.87	1086	67
14	10	102.85	103.10	0.25	518	2.62	5.22	262	51
15	12	123.40	123.85	0.45	756	2.45	5.01	400	53
16	13	56.80	57.65	0.85	1411	2.65	3.75	1021	72
17	15	30.65	30.90	0.25	161	2.83	5.79	96	60
18	16	65.55	65.80	0.25	389	2.71	4.32	211	54
19	19	38.10	38.50	0.40	990	2.09	4.72	433	44
20	22	46.20	46.45	0.25	231	2.69	5.96	122	53

TABLE 34 Candidate regions for population `JPT` vs `YRI` using *XP-EHH* on phased data

	CHR	START	END	LENGTH	#MRK	MEAN	MAX	#EXTR MRK	$\frac{\text{\#EXTR MRK}}{\text{\#MRK}}$ %
1	1	103.05	103.30	0.25	643	2.25	4.52	356	55
2	1	189.35	189.65	0.30	308	2.22	4.00	159	52
3	2	96.10	96.35	0.25	194	2.05	3.31	98	51
4	2	176.60	176.85	0.25	367	2.26	3.87	200	55
5	2	200.00	200.45	0.45	598	2.16	4.36	317	53
6	3	26.10	26.35	0.25	278	2.13	3.60	151	54
7	3	87.10	87.40	0.30	1020	2.02	4.57	510	50
8	3	175.50	175.75	0.25	667	2.10	4.23	363	54
9	4	46.90	47.15	0.25	506	2.22	3.77	255	50
10	7	66.40	67.00	0.60	615	2.43	5.26	390	63
11	7	80.65	80.90	0.25	431	2.11	5.39	224	52
12	10	54.10	54.55	0.45	1615	2.16	4.71	857	53
13	10	102.80	103.25	0.45	867	2.15	4.62	439	51
14	12	1.15	1.50	0.35	1076	2.26	6.14	521	48
15	13	56.85	57.55	0.70	1263	2.24	4.66	711	56
16	15	30.65	30.90	0.25	161	2.26	4.18	86	53
17	16	72.35	72.70	0.35	336	2.11	3.70	169	50
18	17	64.85	65.10	0.25	221	2.24	4.55	119	54
19	19	38.15	38.50	0.35	862	2.12	5.38	435	50
20	22	46.15	46.45	0.30	286	1.91	5.12	132	46

TABLE 35 Candidate regions for population `JPT` vs `YRI` using *Rsb* on phased data

	CHR	START	END	LENGTH	#MRK	MEAN	MAX	#EXTR MRK	$\frac{\text{\#EXTR MRK}}{\text{\#MRK}}$ %
1	1	102.95	103.30	0.35	894	2.03	5.11	423	47
2	1	160.95	161.20	0.25	452	2.08	4.04	226	50
3	1	189.40	189.65	0.25	276	2.03	3.74	141	51
4	2	199.75	200.45	0.70	873	2.25	5.24	536	61
5	3	87.10	87.35	0.25	857	2.00	4.64	434	51
6	3	165.60	165.95	0.35	802	1.85	4.44	361	45
7	3	175.50	175.75	0.25	667	1.90	4.31	335	50
8	4	0.65	1.05	0.40	1096	1.84	5.72	433	40
9	4	46.80	47.20	0.40	721	2.09	3.87	343	48
10	4	98.65	99.30	0.65	1049	2.19	4.82	592	56
11	6	62.60	62.95	0.35	1083	2.12	4.65	577	53
12	6	65.55	65.90	0.35	1240	2.09	5.28	639	52
13	7	66.40	67.00	0.60	615	2.23	7.96	347	56
14	8	84.85	85.20	0.35	588	1.71	4.52	263	45
15	10	102.80	103.20	0.40	786	2.05	4.47	410	52
16	13	56.85	57.65	0.80	1353	2.26	5.25	890	66
17	16	72.30	72.60	0.30	313	2.09	3.19	163	52
18	17	30.60	30.85	0.25	358	1.98	3.67	182	51
19	19	34.55	34.90	0.35	932	1.90	4.55	440	47
20	19	38.15	38.50	0.35	862	1.88	6.03	421	49

TABLE 36 Candidate regions for population `JPT` vs `YRI` using *XP-EHH*/*Rsb* on unphased data

	CHR	START	END	LENGTH	#MRK	MEAN	MAX	#EXTR MRK	$\frac{\text{\#EXTR MRK}}{\text{\#MRK}}$ %
1	1	35.00	35.40	0.40	690	2.53	3.59	322	47
2	2	17.20	17.65	0.45	1023	2.76	3.83	590	58
3	2	108.40	108.75	0.35	886	2.79	4.47	497	56
4	2	134.35	136.20	1.85	3353	3.74	9.92	2630	78
5	2	136.35	136.65	0.30	937	2.68	4.41	478	51
6	3	49.80	50.05	0.25	336	2.72	3.76	178	53
7	3	107.40	107.85	0.45	617	3.06	5.21	405	66
8	4	99.05	99.50	0.45	1008	2.68	4.31	532	53
9	5	118.00	118.50	0.50	1203	2.72	4.14	646	54
10	6	68.20	68.55	0.35	1321	2.62	4.18	762	58
11	6	69.05	69.30	0.25	455	2.80	4.09	253	56
12	8	30.05	30.30	0.25	576	2.53	4.11	290	50
13	12	79.70	80.05	0.35	471	2.67	3.93	248	53
14	12	99.75	100.10	0.35	596	2.31	4.27	262	44
15	13	62.95	63.20	0.25	536	2.66	4.18	285	53
16	15	28.10	28.40	0.30	288	2.63	3.90	145	50
17	15	28.80	29.05	0.25	430	2.80	4.24	262	61
18	15	47.85	48.45	0.60	1065	3.22	7.11	627	59
19	15	74.60	74.90	0.30	427	2.57	3.88	189	44
20	18	7.40	7.75	0.35	895	2.01	4.12	376	42

TABLE 37 Candidate regions for combined population `CEU+GBR` vs `CHB+CHS` using *XP-EHH* on phased data

	CHR	START	END	LENGTH	#MRK	MEAN	MAX	#EXTR MRK	$\frac{\text{\#EXTR MRK}}{\text{\#MRK}}$ %
1	2	17.25	17.75	0.50	1095	2.28	4.23	561	51
2	2	108.40	108.80	0.40	935	2.37	4.89	505	54
3	2	134.30	136.65	2.35	4802	3.36	9.51	3739	78
4	2	176.60	176.90	0.30	589	1.92	4.24	279	47
5	3	107.40	107.85	0.45	617	2.42	5.30	326	53
6	4	99.05	99.55	0.50	1132	2.34	5.11	587	52
7	5	29.20	29.45	0.25	859	2.35	4.91	461	54
8	5	103.95	104.25	0.30	541	2.20	5.38	252	47
9	5	108.85	109.15	0.30	728	2.14	4.52	344	47
10	5	118.25	118.50	0.25	559	2.24	4.21	285	51
11	6	68.20	68.65	0.45	1499	2.41	5.13	814	54
12	6	92.60	93.00	0.40	1485	1.88	4.91	596	40
13	8	32.35	32.60	0.25	986	2.33	5.44	533	54
14	13	21.20	21.50	0.30	609	2.09	3.82	275	45
15	13	62.75	63.15	0.40	1055	2.20	4.16	511	48
16	15	28.15	28.40	0.25	192	2.41	3.93	102	53
17	15	36.05	36.30	0.25	770	2.28	4.07	402	52
18	15	47.80	48.50	0.70	1332	2.65	7.01	801	60
19	15	74.60	74.95	0.35	493	2.12	4.25	224	45
20	18	7.45	7.75	0.30	700	2.05	4.14	330	47

TABLE 38 Candidate regions for combined population `CEU+GBR` vs `CHB+CHS` using *Rsb* on phased data

	CHR	START	END	LENGTH	#MRK	MEAN	MAX	#EXTR MRK	$\frac{\#\text{EXTR MRK}}{\#\text{MRK}}$ %
1	1	35.25	35.60	0.35	501	2.00	4.66	252	50
2	2	17.40	17.70	0.30	626	2.16	3.93	307	49
3	2	108.40	108.75	0.35	886	2.36	5.89	484	55
4	2	134.35	136.25	1.90	3457	3.29	9.24	2680	78
5	3	107.45	107.80	0.35	475	2.21	4.73	228	48
6	3	129.45	129.70	0.25	538	2.14	3.98	269	50
7	4	99.05	99.50	0.45	1008	2.15	4.87	476	47
8	5	108.90	109.15	0.25	644	2.26	4.22	345	54
9	6	28.10	28.35	0.25	747	2.18	3.70	393	53
10	6	68.20	68.70	0.50	1564	2.54	5.72	975	62
11	6	69.05	69.30	0.25	455	2.30	4.96	245	54
12	7	112.45	112.75	0.30	645	2.17	4.63	307	48
13	11	71.20	71.55	0.35	1076	2.05	6.20	445	41
14	13	43.65	44.00	0.35	916	2.05	5.61	394	43
15	13	62.85	63.10	0.25	647	2.24	4.28	330	51
16	14	68.95	69.20	0.25	440	2.39	5.70	246	56
17	15	28.15	28.40	0.25	192	2.44	5.75	107	56
18	15	36.05	36.35	0.30	883	2.18	5.41	457	52
19	15	47.85	48.45	0.60	1065	2.43	7.48	583	55
20	15	74.60	74.90	0.30	427	2.13	4.48	204	48

TABLE 39 Candidate regions for combined population `CEU+GBR` vs `CHB+CHS` using *XP-EHH*/*Rsb* on unphased data
