# Supplementary material for: Detecting selection using extended haplotype homozygosity (EHH)-based statistics in unphased or unpolarized data
Source: PLoS One. 2022 Jan 18;17(1):e0262024. doi: 10.1371/journal.pone.0262024 (PMC8765611; doi:10.1371/journal.pone.0262024)
Supplement: S1 Protocol — (PDF) [file pone.0262024.s003.pdf]

S2 protocol. Supporting Information on software and technical details

for

Detecting selection using extended haplotype homozygosity (EHH)-based statistics in unphased or unpolarized data

A. Klassmann and M. Gautier

1 | SIMULATED DATA

We performed coalescent simulations using msms [1]. We assumed a population-scaled mutation and a recombination rate of $\theta = \rho = 0.001$ per base and set the chromosome length to 50 Mb. We set the output format for site positions to the highest possible precision in order to avoid different segregating sites being printed with the same (rounded) position. The commands have been (with appropriate modifications of sample sizes):

- for a sample of size 100 from a single, neutrally evolving population:

```
msms 100 1 -N 10000 -t 50000 -r 50000 50000000 -oformat "#.################"
```

- for a sample of size 200 containing half of chromosomes from each of two neutrally evolving populations which split symmetrically from an ancestral population $4N_e \cdot 0.05$ generations ago, without subsequent migration (this time corresponds roughly to $50,000$ years in humans):

```
msms 200 1 -N 10000 -t 50000 -r 50000 50000000 -I 2 100 100 -ej 0.05 1 2 -oformat "#.################"
```

- for a sample of size 200 from a population experiencing a single on-going selective sweep while otherwise evolving neutrally. The selected allele was set as dominant with a population-scaled selection coefficient of $2N_e s = 500$, having reached at sampling time a population frequency of 50% (70%, 90%, respectively) or was sampled 0.01 population-scaled time units after fixation. The site was located exactly at the center of the simulated chromosome (position 0.5). The selected site itself was included into the output:

```
msms 200 1 -N 10000 -t 50000 -r 50000 50000000 -SAA 500 -SaA 500 -SF 0 0.5 -Sp 0.5 -Smark
-oformat "#.################"
```

Since the polymorphic sites simulated by msms have positions in the interval (0,1], they were multiplied by $5 \cdot 10^7$ in order to yield statistics comparable to human population genetic data.

We repeated the neutral simulations using msprime [2] with settings:

- the single neutrally evolving population

```
Ne = 1e4
length = 5e7
theta = 5e4
rho = 5e4
sample_size = 100
msprime.simulate(sample_size = sample_size,
                 Ne = Ne,
                 length = length,
                 recombination_rate = rho / (4 * Ne * length),
                 mutation_rate = theta / (4 * Ne * length),
                 num_replicates = 100,
                 model = "dtwf")
```

- two neutrally evolving populations resulting from a symmetric split

```
split_time = 0.05
msprime.simulate(Ne = Ne,
                 length = length,
                 recombination_rate = rho / (4 * Ne * length),
                 mutation_rate = theta / (4 * Ne * length),
                 num_replicates = 100,
                 model = "dtwf",
                 population_configurations=[
                    msprime.PopulationConfiguration(sample_size = int(sample_size / 2)),
                    msprime.PopulationConfiguration(sample_size = int(sample_size / 2))],
                 demographic_events=[
                    msprime.MassMigration(split_time * Ne * 4, source=1, dest=0, proportion=1)])
```

The calculation of the original and modified *EHH*-based statistics *iHS*, *XP-EHH* and *Rsb* as stated in the main text, were calculated by rehh version 3.1 (which can read ms output). In the function `scan_hh()` the parameters `limehh`/`limehhs` (integration cutoff with respect to the EHH/EHHS value) and `limhomohaplo` (integration cutoff with respect to the number of remaining homozygote sequences) were set according to the information in the main text. Whether sequences could be assumed as phased and variants as polarized was marked by the eponymous options of that function. Furthermore, option `discard_integration_at_border` was set to `FALSE` which implies that integration is stopped at gaps and sequence borders, but the value computed up to that position is reported and not discarded. The calculation of *iHS* from *iHH* on unpolarized data was performed by setting in function `ihh2ihs()` option `freqbin` to 1 (a single frequency-bin) while leaving the default option of 0.025 (even spaced frequency-bins of width 0.025) for polarized data. All other parameters, including those of the functions for the calculation of *XP-EHH* and *Rsb* were left at their default values.

For the manipulation of files in ms format we used and supplemented command line tools of the C++ package coatli[1] (version 1.0.6) [3]:

---

[1] Downloadable from `https://sourceforge.net/projects/coatli/`

- in order to generate subsamples of size *n* (extract the first *n* chromosomes) from ms format with output in the same format

  ```
  mssub -precision 16 [n]
  ```

  (we could not find any bias due to this kind of subsetting in previous studies and we checked that a selected allele is neither over- nor underrepresented in subsamples)

- to transform ms output into fastPHASE input

  ```
  ms2fastphase -r -n
  ```

  this command assigns each two consecutive chromosomes to a diploid individual and switches at each segregating site with probability of 50% the alleles between the two chromosomes. The second option implies that no id number for individuals is generated.

- to calculate from ms output the allele counts needed for SweepFinder / SweeD

  ```
  ms2SF -length 50000000 -precision 16
  ```

  ```
  ms2SF -length 50000000 -precision 16 -folded
  ```

  The latter option merely marks each variant as unpolarized, specified by a 1 instead of a zero in the last column of the format.

The phase information was re-constructed from genotypes using fastPHASE version 1.4.8 [4], with 10 random starts instead of the default 20, as recommended by the manual to save time without much loss of accuracy:

```
fastPHASE -T10 -n -o[output-prefix] [inputfile]
```

The output files of fastPHASE were read in by rehh and the (original) statistics computed with parameters as stated above.

SweepFinder2 [5] and SweeD [6] provide different computational implementations of the same statistic, a Composite Likelihood Ratio (CLR). Because of its better performance, we used SweeD version 4.0.0 for the frequency spectra marked as polarized. Unfortunately, we found a bug concerning spectra which were marked as folded (we tested also the previous version 3.2.1) and hence we used SweepFinder2 version 1.0 for the latter. We let the programs calculate the statistic at 25,000 positions uniformly spread between the position of the first and the last polymorphism, hence consecutive positions were separated by slightly less than 2 kb. The commands were:

- for data marked as polarized

```
SweeD-P -grid 25000 -input [inputfile] -name [output-suffix] -threads [nthreads]
```

- for data marked as unpolarized

```
SweepFinder2 -s 25000 [inputfile] [outputfile]
```

With these commands, the observed empirical spectrum of each simulated chromosome served as its own "background spectrum". We opted for this solution, because the individual chromosomes were simulated with the same parameters and long enough to yield a reliable background. In a realistic scan on empirical data, though, the background spectrum is recommended to derive from the whole genome.

Tajima's D [7] and Fay & Wu's H [8] have been computed using rehh version 3.2 with a sliding window of size 50 kb in steps of 25 kb.

2 | EMPIRICAL DATA

We used release `20181203_biallelic_SNV` of variants called by [9] on reads from phase 3 of the 1000 Genomes Project [10], realigned to Human reference assembly hg38. The data comprise phased bi-allelic SNPs of 2548 individuals from 26 populations. Population specific files were extracted using vcftools [11], version 0.1.17.

Phased variants for unrelated individuals of population `MKK` (used for Figure S18) were obtained from HapMap3 project [12], release 2. The files were transformed into Variant Call Format (VCF) [11] by costum shell scripts and coordinates translated from Human genome assembly hg18 to hg38 using the Genome Analysis Toolkit (GATK) [13] version 4.1.8.0 and the appropriate UCSC Chain file [14].

For the polarization of variants, we used the inferred Human ancestral sequence of ENSEMBL release 91 [15]. The ancestral variants were added as key `AA` to the `INFO` field in the VCF files by custom shell scripts.

For the calculation of the *EHH*-based statistics on the population samples, we used rehh version 3.1 with options identical to those for the simulated data stated above, except an additional setting of `maxgap = 100000` in function `scan_hh()` to stop integration of *EHH*/*EHHS* at gaps between consecutive markers larger than 100 kb (in simulated data no such gaps occur).

References

[1] Ewing G, Hermisson J. MSMS: a coalescent simulation program including recombination, demographic structure and selection at a single locus. Bioinformatics. 2010;26(16):2064–5. doi:10.1093/bioinformatics/btq322.

[2] Kelleher J, Etheridge AM, McVean G. Efficient Coalescent Simulation and Genealogical Analysis for Large Sample Sizes. PLoS Computational Biology. 2016;12(5):1–22. doi:10.1371/journal.pcbi.1004842.

[3] Klassmann A, Ferretti L. The third moments of the site frequency spectrum. Theoretical Population Biology. 2018;120:16–28. doi:10.1016/j.tpb.2017.12.002.

[4] Scheet P, Stephens M. A fast and flexible statistical model for large-scale population genotype data: Applications to inferring missing genotypes and haplotypic phase. American Journal of Human Genetics. 2006;78(4):629–644. doi:10.1086/502802.

[5] DeGiorgio M, Huber CD, Hubisz MJ, Hellmann I, Nielsen R. SweepFinder2: increased sensitivity, robustness and flexibility. Bioinformatics. 2016;32(12):1895–1897. doi:10.1093/bioinformatics/btw051.

[6] Pavlidis P, Živković D, Stamatakis A, Alachiotis N. SweeD: likelihood-based detection of selective sweeps in thousands of genomes. Molecular Biology and Evolution. 2013;30(9):2224–34. doi:10.1093/molbev/mst112.

[7] Tajima F. Statistical method for testing the neutral mutation hypothesis by DNA polymorphism. Genetics. 1989;123(3):585–95.

[8] Fay JC, Wu CI. Hitchhiking under positive Darwinian selection. Genetics. 2000;155(3):1405–13.

[9] Lowy-Gallego E, Fairley S, Zheng-Bradley X, Ruffier M, Clarke L, Flicek P, et al. Variant calling on the grch38 assembly with the data from phase three of the 1000 genomes project [version 2; peer review: 2 approved]. Wellcome Open Research. 2019;4:1–41. doi:10.12688/wellcomeopenres.15126.1.

[10] The 1000 Genomes Project Consortium. A global reference for human genetic variation. Nature. 2015;526(7571):68–74. doi:10.1038/nature15393.

[11] Danecek P, Auton A, Abecasis G, Albers CA, Banks E, DePristo MA, et al. The variant call format and VCFtools. Bioinformatics. 2011;27(15):2156–2158. doi:10.1093/bioinformatics/btr330.

[12] The international HapMap Consortium. Integrating common and rare genetic variation in diverse human populations. Nature. 2010;467(7311):52–8. doi:10.1038/nature09298.

[13] DePristo Ma, Banks E, Poplin R, Garimella KV, Maguire JR, Hartl C, et al. A framework for variation discovery and genotyping using next-generation DNA sequencing data. Nature Genetics. 2011;43(5). doi:10.1038/ng.806.

[14] Kuhn RM, Haussler D, James Kent W. The UCSC genome browser and associated tools. Briefings in Bioinformatics. 2013;14(2):144–161. doi:10.1093/bib/bbs038.

[15] Zerbino DR, Achuthan P, Akanni W, Amode MR, Barrell D, Bhai J, et al. Ensembl 2018. Nucleic Acids Research. 2018;46(D1):D754–D761. doi:10.1093/nar/gkx1098.
